# Culture-Dependent Microbiome of the *Ciona intestinalis* Tunic: Isolation, Bioactivity Profiling and Untargeted Metabolomics

**DOI:** 10.3390/microorganisms8111732

**Published:** 2020-11-05

**Authors:** Caroline Utermann, Vivien A. Echelmeyer, Martina Blümel, Deniz Tasdemir

**Affiliations:** 1 GEOMAR Centre for Marine Biotechnology (GEOMAR-Biotech), Research Unit Marine Natural Products Chemistry, GEOMAR Helmholtz Centre for Ocean Research Kiel, Am Kiel-Kanal 44, 24106 Kiel, Germany; cutermann@geomar.de (C.U.); vivienechelmeyer@web.de (V.A.E.); mbluemel@geomar.de (M.B.); 2 Faculty of Mathematics and Natural Sciences, Kiel University, Christian-Albrechts-Platz 4, 24118 Kiel, Germany

**Keywords:** *Ciona intestinalis*, tunic, marine microorganisms, antimicrobial activity, anticancer activity, metabolomics, feature-based molecular networking

## Abstract

Ascidians and their associated microbiota are prolific producers of bioactive marine natural products. Recent culture-independent studies have revealed that the tunic of the solitary ascidian *Ciona* *intestinalis* (sea vase) is colonized by a diverse bacterial community, however, the biotechnological potential of this community has remained largely unexplored. In this study, we aimed at isolating the culturable microbiota associated with the tunic of *C.* *intestinalis* collected from the North and Baltic Seas, to investigate their antimicrobial and anticancer activities, and to gain first insights into their metabolite repertoire. The tunic of the sea vase was found to harbor a rich microbial community, from which 89 bacterial and 22 fungal strains were isolated. The diversity of the tunic-associated microbiota differed from that of the ambient seawater samples, but also between sampling sites. Fungi were isolated for the first time from the tunic of *Ciona*. The proportion of bioactive extracts was high, since 45% of the microbial extracts inhibited the growth of human pathogenic bacteria, fungi or cancer cell lines. In a subsequent bioactivity- and metabolite profiling-based approach, seven microbial extracts were prioritized for in-depth chemical investigations. Untargeted metabolomics analyses of the selected extracts by a UPLC-MS/MS-based molecular networking approach revealed a vast chemical diversity with compounds assigned to 22 natural product families, plus many metabolites that remained unidentified. This initial study indicates that bacteria and fungi associated with the tunic of *C.* *intestinalis* represent an untapped source of putatively new marine natural products with pharmacological relevance.

## 1. Introduction

Marine organisms are highly valuable sources for bioactive natural products (NPs) [1,2] and have yielded about 30,000 compounds so far [3]. With over 1000 described marine natural products (MNPs), ascidians (phylum Chordata, subphylum Tunicata) range among the most prolific producers of MNPs [4,5,6]. Being soft-bodied, sessile organisms, ascidians rely on chemical defense strategies that involve secondary metabolites for repelling predators, pathogens, and fouling organisms [6,7,8]. The tunic, the outermost tissue of ascidians, represents the initial defense barrier [9,10]. Similar to other marine living surfaces, the tunic is the site of various chemical communications and of particular interest for discovery of bioactive MNPs [11,12]. Ascidians are holobionts [6,13] that are hosts to a diverse, stable and species-specific microbial community [14,15]. Accordingly, many secondary metabolites originally isolated from ascidians are nowadays believed to be produced by symbiotic microorganisms [4,6,7]. Strikingly, the majority of MNPs derived from ascidian-associated microbes show potent bioactivities, in particular cytotoxicity and antimicrobial activities [6,16]. The most prominent examples of anticancer MNPs of bacterial origin include the alkaloid trabectedin, the source of the approved anticancer drug Yondelis^®^, and the cyclic peptide didemnin B that once progressed to phase II clinical trial as anticancer drug candidate [1,16,17,18]. Trabectedin is produced by the *Ecteinascidia turbinata* symbiont *Candidatus* Endoecteinascidia frumentensis [1,16,17] and didemnin B was suggested to originate from culturable bacteria affiliated to *Tistrella* spp. rather than from its original source, the ascidian *Trididemnum solidum* [16,18]. Moreover, the polyketide arenimycin that inhibits multidrug-resistant *Staphylococcus aureus* [19] is only one out of several antibiotics produced by actinobacteria associated with *E. turbinata* [16,20,21]. Another example is trichodermamide B, an antimicrobial and cytotoxic dipeptide that was isolated from the *Didemnum molle*–associated fungus *Trichoderma virens* [22].

*Ciona intestinalis* (family Cionidae; formerly *C. intestinalis* type B), also known as sea vase, is a solitary tunicate distributed in the North Atlantic Ocean as well as Baltic, North, Bohai and Yellow Seas [23,24,25]. The sea vase is one of the most notorious invasive species with cross-continental expansion in the northern hemisphere causing significant ecological and economic problems [23,25,26]. Due to its vertebrate-like larvae, rapid embryogenesis, translucent body, short life cycle, and its fully sequenced genome, it is also a popular model organism for developmental biology [23,27]. Little is known about the chemical inventory of *Ciona* spp. [5], but a few compounds with promising biological activities have been reported, such as the cytotoxic metabolite iodocionin [28] and the antimicrobial peptide Ci-MAM-A24 [29]. Previous culture-independent microbiome studies have demonstrated a broad bacterial diversity associated with the tunic of *Ciona* spp. [9,30], however, only a few reports are available on the isolation of bacterial strains from the tunic of *Ciona* spp. [9,16,31]. Indeed, the gammaproteobacterium *Pseudoalteromonas tunicata* represents the only example of a tunic-associated bacterial isolate from *C. intestinalis* producing metabolites with antibacterial and antifouling activities [31,32].

In order to fill this gap, this study investigated the culture-dependent microbial diversity associated with the tunic of the solitary ascidian *C. intestinalis* and gained first insights into the biotechnological potential of the culturable tunic-associated microbiota. Taking the large adaptive capacity of *C. intestinalis* and its microbiome to a broad range of environmental conditions into account, we selected two collection sites: a location in the North Sea (Helgoland) with marine salinity (~30 psu) and another collection site in the Baltic Sea (Kiel Fjord) characterized as brackish (~18 psu). A culture-dependent approach yielded overall 111 tunic-associated isolates, of which 89 were bacterial and 22 were fungal strains. In addition, microbes were isolated from seawater samples (bacteria: 92 isolates, fungi: 9 isolates), which served as reference for comparison of the microbial diversity of the ascidian’s tunic. As ascidian-associated microbes have previously yielded novel metabolites with promising antibiotic and anticancer activities, the organic extracts of tunic-derived strains were tested against a panel of human pathogens (bacteria and fungi) and cancer cell lines. The most bioactive and promising extracts were selected and subjected to an UPLC-MS/MS-based untargeted metabolomics study. The putative annotation of known MNPs was aided by automated dereplication tools such as feature-based molecular networking (FBMN; [33]) and the *in-silico* MS/MS database-based (ISDB) dereplication pipeline [34]. By employing the bioactivity and chemical diversity as main filters, several promising extracts were prioritized for in-depth chemical studies in future.

## 2. Materials and Methods

### 2.1. Sampling

Specimens of *C. intestinalis* were sampled in September 2017 in Helgoland (Germany, North Sea; 54.177102, 7.893053) and Kiel Fjord (Germany, Baltic Sea; 54.382062, 10.162059). In Helgoland, samples were collected from below a pontoon by scuba diving (<1 m) and in Kiel from an overgrown mussel-cultivation basket at approximately 3 m depth. Seawater reference samples were collected aseptically at the same sites. Ascidian and water samples were immediately transported to the local laboratory and processed on the same day.

### 2.2. Isolation of Microorganisms

To isolate a broad diversity of bacteria and fungi from *C. intestinalis*, we used six different agar media (1.8% agar each). Two of the media were designed to mimic the original habitat of the microorganisms, namely *C. intestinalis* media adjusted to Baltic (CB) or North Sea (CN) salinity. Therefore, *C. intestinalis* was freeze-dried (Alpha 2-4 LSC, Martin Christ Gefriertrocknungsanlagen, Osterode, Germany) at 0.52 mbar and −80 °C. The freeze-dried material was processed into a semi-coarse powder using a pulverisette (Pulverisette 14, sieve ring p-14, 1 mm pore size, trapezoidal perforation; Fritsch, Idar-Oberstein, Germany). 1.5% of *C. intestinalis* powder was added and the salt concentration was adjusted to the salinity of the Baltic Sea (1.8% Instant Ocean (Blacksburg, VA, USA)) or of the North Sea (3% Instant Ocean).

The other four solid media used were MB (3.74% Marine Broth 2216), PDA (potato dextrose agar) [35], TSB (0.3% trypticase soy broth, 1% sodium chloride) and modified WSP (Wickerham medium) [36]. Ingredients were purchased from AppliChem (Darmstadt, Germany; agar bacteriology grade, sodium chloride), Becton Dickinson (Sparks, MD, USA; Marine Broth 2216, malt extract, trypticase soy broth), Merck (Darmstadt, Germany; D (+)-glucose monohydrate, peptone from soymeal, yeast extract granulated) and Sigma Aldrich (Steinheim, Germany; potato infusion powder). In order to remove planktonic bacteria loosely attached to the tunic, the tunic was thoroughly rinsed with sterile artificial seawater (3% and 1.8% Instant Ocean for Helgoland and Kiel samples, respectively) prior to dissection. Four individuals per sampling site were selected and their tunic was removed using sterilized scissors. Two different strategies, i.e., tissue homogenization and imprinting, were applied for isolation of tunic-associated microbes. For homogenization, the tunic tissue was placed into a sterile 15 mL reaction tube, which was filled to a final ratio of 1:1:1 with glass beads (0.5–2 mm diameter) and sterile artificial seawater (*n* = 2). The mixture was homogenized for 2 min at 2000 rpm on a Vortex mixer (HS120212, Heathrow Scientific, IL, USA). Homogenates (original concentration) and their 1:10 and 1:100 dilutions were plated as 100 µL aliquots onto the different agar media. For imprinting, tunic samples were imprinted on the respective agar plates (*n* = 2). Additionally, 100 µL and 500 µL aliquots of seawater reference samples were plated in duplicate. Inoculated petri dishes were kept for three weeks in the dark at 22 °C. After one week and after three weeks, all plates were evaluated and different colony morphotypes were selected for purification. The selected isolates were transferred to fresh medium until pure cultures were obtained. Purified strains were cryopreserved at −80 °C until further analyses by using the ready-to-use Microbank^TM^ system (Pro Lab Diagnostics, Richmond Hill, ON, Canada).

### 2.3. Identification of Bacterial and Fungal Strains

DNA extraction of bacteria and fungi was performed as described previously [37]. A slight modification in the respective protocols was applied by repeating the centrifugation step. When the DNA extraction was not successful, the extraction process was repeated by using the DNeasy Plant Mini Kit (Qiagen, Hilden, Germany). For this, bacterial strains were cultivated for 2 days in liquid MB medium and fungal strains for 5 days in liquid PDA. A 2 mL subsample of the culture was centrifuged for 10 min at 5000 *g* and the supernatant was discarded. The cell pellet was incubated with 400 µL AP1 buffer, 4 µL RNase A, and 4 µL Proteinase K (20 mg/mL, Analytik Jena, Jena, Germany) for approximately 2 h at 65 °C and rotation at 700 rpm (TMix 220, Analytik Jena). Afterwards, DNA extraction was performed according to the manufacturer’s instructions from step 9 onwards. DNA was eluted using 50 µL AE buffer and step 19 was skipped. PCR amplification of bacterial and fungal DNA was realized by using universal primers amplifying the 16S rRNA gene or the ITS1-5.8S-ITS2 region as described before [37]. Those fungal specimens, for which ITS1-2 sequencing did not allow identification at genus level, were additionally amplified with primers spanning the small (18S) and large (28S) subunit of the rRNA gene [38,39]. The protocol for amplification of the 28S rRNA gene [39] was modified as follows: initial denaturation at 94 °C for 3 min, 35 cycles of denaturation (94 °C, 1 min), annealing (55 °C, 30 s), and elongation (72 °C, 2 min), as well as a final elongation step at 72 °C for 5 min. PCR products were Sanger sequenced [40] at LGC Genomics GmbH (Berlin, Germany). Sequences were trimmed and transformed to FASTA format with ChromasPro V1.33 (Technelysium Pty. Ltd., South Brisbane, Australia). FASTA files were submitted to BLAST (Basic Local Alignment Search Tool, [41]) at NCBI (National Center for Biotechnology Information). Whenever BLAST comparison did not allow identification of bacteria to genus level, FASTA sequences were additionally submitted to the Naive Bayesian rRNA Classifier v2.11 of the Ribosomal Database Project (RDP, [42]). The taxonomical hierarchy was inferred at a 95% confidence threshold with the RDP 16S rRNA training set. DNA sequences of all microbial isolates are available in GenBank under the accession numbers MW012283-371 (tunic-associated bacteria), MW012374-78 (tunic-associated fungi, 18S), MW012380-87 (seawater-derived fungi, ITS), MW013337-428 (seawater-derived bacteria), MW014884-87 (seawater-derived fungi, 18S), MW017476-94 (tunic-associated fungi, ITS), MW017496-97 (tunic-associated fungi, 28S), and MW017498-99 (seawater-derived fungi, 28S).

### 2.4. Cultivation of Tunic-Associated Microbial Strains

In total, 111 microbial strains were isolated from the tunic of *C. intestinalis*. Safety level determination in accordance with the German safety guidelines TRBA 460 (Technical Rules for Biological Agents, July 2016) and TRBA 466 (August 2015) excluded 19 strains from further analyses. When ≥2 strains belonged to the same species, only one representative strain was selected, leading to the exclusion of another 23 strains. Hence, 69 tunic-associated strains were cultivated on 2 different media: bacteria were grown on glucose-yeast-malt (GYM) [43] and MB media while the fungal isolates were grown on casamino-acids-glucose (CAG) [44] and PDA media. If not stated otherwise, ingredients for CAG and GYM were purchased at Carl Roth (Karlsruhe, Germany). CAG, GYM, and PDA media were selected as culture media, since they proved in previous studies as particularly suitable for production of a variety of novel bioactive compounds (e.g., [35,36,45]). The commonly used MB medium was selected in addition to ensure sufficient growth of all bacterial isolates for chemical investigations. Precultures were inoculated by streaking a bead from the cryo-preservation tube onto the respective solid agar media, which was then grown in the dark at 22 °C until the agar was completely covered by microbial colonies. For main cultures, 5 (fungi) or 10 (bacteria) agar plates per strain were inoculated on each medium in duplicate (i.e., 20 or 40 plates per strain) by gentle streaking with an inoculation loop. For colonies that could not be transferred by an inoculation loop, a small piece of overgrown agar was cut and streaked onto the main culture plates. Main cultures were incubated in the dark at 22 °C for 7 (bacteria) or 21 days (fungi). Notably, 61% of the bacterial strains did not grow on GYM and were hence only cultivated on MB. 

### 2.5. Solvent Extraction

The agar was cut into pieces with a flat spatula and transferred into a glass bottle. Following the addition of ethyl acetate (EtOAc; VWR International, Leuven, Belgium) to fungal (200 mL) and bacterial cultures (400 mL), the mixture was homogenized for 30 s at 13,000 rpm (T25 basic Ultra Turrax IKA-Werke, Staufen, Germany). Homogenization was followed by maceration overnight in the dark at 120 rpm and 22 °C. EtOAc was decanted into a separatory funnel and partitioned against the equal volume of ultra-purified water (Arium Lab water systems, Sartorius, Goettingen, Germany) to remove mainly salts and water-soluble media ingredients. The aqueous phase was discarded and the EtOAc phase was collected in a round bottom flask. Another 200 or 400 mL EtOAc was added to the agar and after 15 min sonication, a second round of extraction was performed. The EtOAc extracts were combined and evaporated to dryness using a rotary evaporator. Dried extracts were resuspended in 4 mL methanol (MeOH; ULC-MS grade, Biosolve Chimie, Dieuze, France), filtered into pre-weighed vials through a 0.2 μm PTFE filter (VWR International, Darmstadt, Germany) and re-dried under nitrogen blow. Vials were stored at −20 °C until further processing. Extracts were coded as follows: *C. intestinalis* (C), location (Helgoland = H or Kiel = K), tissue (tunic = T), strain number and medium (CAG, GYM, MB, PDA), e.g., CHT56-CAG refers to the extract of strain 56 isolated from the tunic of *C. intestinalis* sampled in Helgoland cultured on medium CAG. As a control, the four different cultivation media were extracted using the same protocol. 

### 2.6. Bioactivity Screening

Crude extracts were screened for antimicrobial and anticancer activities. For this aim, dried organic crude extracts were re-dissolved in dimethyl sulfoxide (DMSO; Carl Roth) at a concentration of 20 mg/mL. The antimicrobial test panel comprised the pathogenic yeast *Candida albicans* (Ca, DSM 1386), the yeast-like fungus *Cryptococcus neoformans* (Cn, DSM 6973), and the bacterial ESKAPE panel (*Enterococcus faecium*, Efm, DSM 20477; methicillin-resistant *Staphylococcus aureus*, MRSA, DSM 18827; *Klebsiella pneumoniae*, Kp, DSM 30104; *Acinetobacter baumannii*, Ab, DSM 30007; *Pseudomonas aeruginosa*, Psa, DSM 1128; *Escherichia coli*, Ec, DSM 1576). Since none of the tested crude extracts showed inhibition of the Gram-negative pathogens (Kp, Ab, Psa, Ec), only results from bioassays against Gram-positive test strains (MRSA, Efm) are described herein. Anticancer activities were assessed by testing inhibition of proliferation of the following cell lines: A375 (malignant melanoma cell line), A549 (lung carcinoma cell line), HCT116 (colon cancer cell line), and MB231 (human breast cancer line MDA-MB231). Test organisms and cell lines were ordered either at Leibniz Institute DSMZ-German Collection of Microorganisms and Cell Cultures (Braunschweig, Germany) or at CLS-Cell Lines Service (Eppelheim, Germany). All bioassays were performed in 96-well microplates at a final extract concentration of 100 µg/mL, as described previously [46]. The following chemicals were used as positive controls: chloramphenicol (MRSA), ampicillin (Efm), nystatin (Ca), amphotericin (Cn), and doxorubicin (cancer cell lines). The half maximal inhibitory concentration (IC_50_) was determined as previously described [46] for extracts selected for in-depth metabolomic analyses (for selection see results Section 3.3.).

### 2.7. UPLC–QToF–MS/MS Analyses

Chemical diversity of the selected bioactive crude extracts was explored via an untargeted UPLC-QToF-MS/MS-based metabolomics approach. ULC-MS grade solvents were purchased from Biosolve Chimie or from LGC Standards (Wesel, Germany). LC-MS/MS analyses were performed on an Acquity UPLC I-Class system coupled to a Xevo G2-XS QToF mass spectrometer (Waters, Milford, MA, USA), equipped with an Acquity UPLC HSS T3 column (High Strength Silica C18, 1.8 μm, 2.1 × 100 mm, Waters) operating at 40 °C. Crude extracts were dissolved in MeOH at a concentration of 1.0 mg/mL and the injection volume was 0.3 µL. A binary mobile phase system (A: 0.1% formic acid in ultra-purified water, B: 0.1% formic acid in acetonitrile) was pumped at a flow rate of 0.6 mL/min by applying a linear gradient (% of A given): initial, 99%; 11.5 min, 1%; 14.5 min, 1%; washing and reconditioning of the column until 16 min. Acquisition of MS and MS/MS spectra was performed as previously described [47], despite the following modifications: spectra were recorded in positive mode and the acquisition range was set to *m/z* 50–1200. The capillary voltage was kept at 3 kV. Solvent (MeOH) and media controls (CAG, GYM, MB, PDA) were analyzed using the same conditions.

### 2.8. Bioinformatic Processing and Dereplication Workflow

Acquired LC-MS/MS data were converted to the mzXML format using the ProteoWizard tool msconvert 3.0.20010 [48]. The publicly available software MZmine 2 [49] was used to denoise data and for automatic generation of peak lists (for parameters see Table S1). Compounds also detected in MeOH or media blanks were removed from the peak lists. Comparative analysis of the metabolite profiles based on generated peak lists was performed by PCoA plotting (Euclidean distance) in Past v3.12 [50] and sample clustering was statistically tested using ANOSIM (Euclidean distance).

Metabolite profiling led to selection of seven crude extracts for in-depth dereplication (CHT56-CAG, CHT58-PDA, CKT35-PDA, CKT43-GYM and -MB, CKT91-CAG and -PDA; for selection see results Section 3.3.). Pre-processed MS/MS data were exported in MGF format, uploaded to the Global Natural Products Social Molecular Networking (GNPS) online platform [51] and submitted to the FBMN workflow [33]. Consensus spectra were constructed with a parent mass tolerance and a MS/MS fragment ion tolerance of 0.02 Da. Edges of the MN were filtered to have a cosine score >0.7 (CHT58: 0.8) and more than 6 matched peaks. Cytoscape v3.7.1 [52] was used for visualization of the computed FBMN.

For identification of known chemical scaffolds, LC-MS/MS chromatograms were inspected manually and putative molecular formulae were predicted by MassLynx v4.1 (Waters). The obtained molecular formulae were compared against common natural products databases (Dictionary of Natural Products (DNP): http://dnp.chemnetbase.com, MarinLit: http://pubs.rsc.org/marinlit/, The Natural Products Atlas (NP Atlas) [53] and Reaxys: https://www.reaxys.com). In addition, automated dereplication of detected metabolites was realized via the GNPS dereplication workflow and the ISDB dereplication pipeline [34]. Putative hits were validated based on the criteria biological origin, retention time and -if detected- their fragmentation pattern, which was aided by the in-silico fragmentation prediction tool CFM-ID [54].

## 3. Results

### 3.1. The Culture-Dependent Microbial Diversity of C. intestinalis

**Figure 1:**
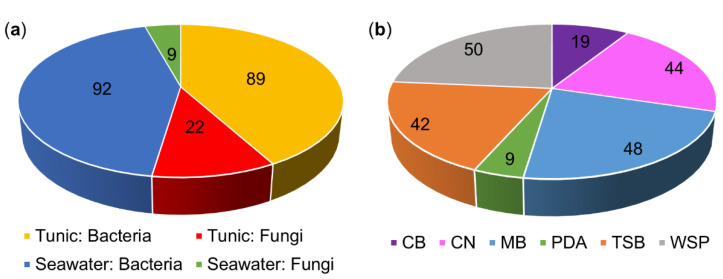
Number of microbial strains isolated from the tunic of *C. intestinalis* and seawater reference. Numbers are given separately for bacteria and fungi from tunic and seawater samples (**a**) and for the six different cultivation media (**b**). CB: *C. intestinalis* medium adjusted to the salinity of the Baltic Sea, CN: *C. intestinalis* medium adjusted to the salinity of the North Sea, MB: marine broth, PDA: potato dextrose agar, TSB: trypticase soy broth and WSP: modified Wickerham medium.

In total, 111 bacterial and fungal isolates were obtained from the tunic of *C. intestinalis* and 101 from seawater references sampled at two sites (Figure 1a, Table S2). Bacteria clearly dominated the strain collection (85%). Both tunic and seawater samples collected in the Baltic Sea (Kiel Fjord) yielded a higher number of isolates (tunic: 53 bacteria and 14 fungi; seawater: 63 bacteria and 4 fungi) than the Helgoland samples (tunic: 36 bacteria and 8 fungi, seawater: 29 bacteria and 5 fungi; Figure S1). Application of different isolation media revealed considerable differences with respect to the number of isolates obtained (Figure 1b). Most strains were isolated on WSP (24%) and MB (23%) media, whereas PDA medium yielded the least number of isolates (4%). One third of isolates was derived from the *C. intestinalis* media adjusted to Baltic Sea (CB) and North Sea salinity (CN). Moreover, 19 strains, e.g., the tunic-associated microbes *Kiloniella laminariae* (CKT60, Alphaproteobacteria) and *Pithomyces chartarum* (CKT81, Dothideomycetes; Table S2) were only retrieved from the isolation media CB and CN. Hence, the in-house designed *C. intestinalis* media CB and CN proved successful, highlighting the importance of mimicking the environmental conditions for isolation of a diverse microbial community [55,56].

Phylogenetic analyses assigned the tunic- and seawater-derived isolates to six different microbial phyla (bacteria: Actinobacteria, Bacteroidetes, Firmicutes, Proteobacteria; fungi: Ascomycota, Basidiomycota), which can be further split up into 30 orders and 82 genera (Table S2). Seven isolates were identified only to a higher taxonomic rank (family or order level).

**Figure 2:**
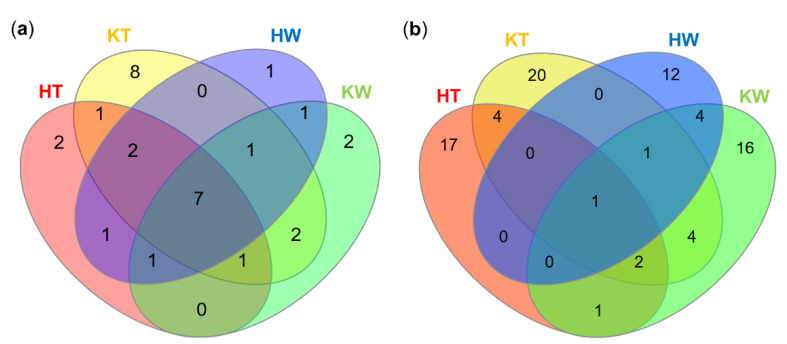
Venn diagrams showing the number of shared and exclusive microbial taxa across sample types and sampling locations. The distribution of taxa is given for microbial orders (**a**) and genera (**b**). H, Helgoland; K, Kiel; T, Tunic; W, Seawater.

Seven bacterial orders (Alteromonadales, Bacillales, Corynebacteriales, Flavobacteriales, Micrococcales, Rhodobacterales, Vibrionales) were detected in all samples (Figure 2a, Figures S2 and S3). Vibrionales was the most abundant order across the four different samples. The tunics of *C. intestinalis* sampled in Kiel showed by far the highest microbial diversity with isolates being affiliated to 22 different microbial orders, of which eight were exclusive to this sample type (bacteria: Burkholderiales, Enterobacterales, Kiloniellales, Xanthomonadales, Streptomycetales; fungi: Glomerellales, Helotiales, Microascales). Tunic samples from Helgoland specimens contained only two exclusive orders, i.e., Caulobacterales and Leotiomycetes *incertae sedis*. Tunic samples from Helgoland and Kiel Fjord yielded a higher microbial diversity than seawater reference samples, where microbial orders exclusive to the tunic (HT = 2, KT = 8, shared = 1) exceeded those exclusive to seawater samples (HW = 1, KW = 2, shared = 1; Figure 2a). The exclusive microbial orders of the ambient seawater samples were affiliated to the fungal orders Chaetosphaeriales (KW), Filobasidiales (HW), Sakaguchiales (KW), and the bacterial order Sphingomonadales (HW and KW; Figures S2 and S3).

**Figure 3:**
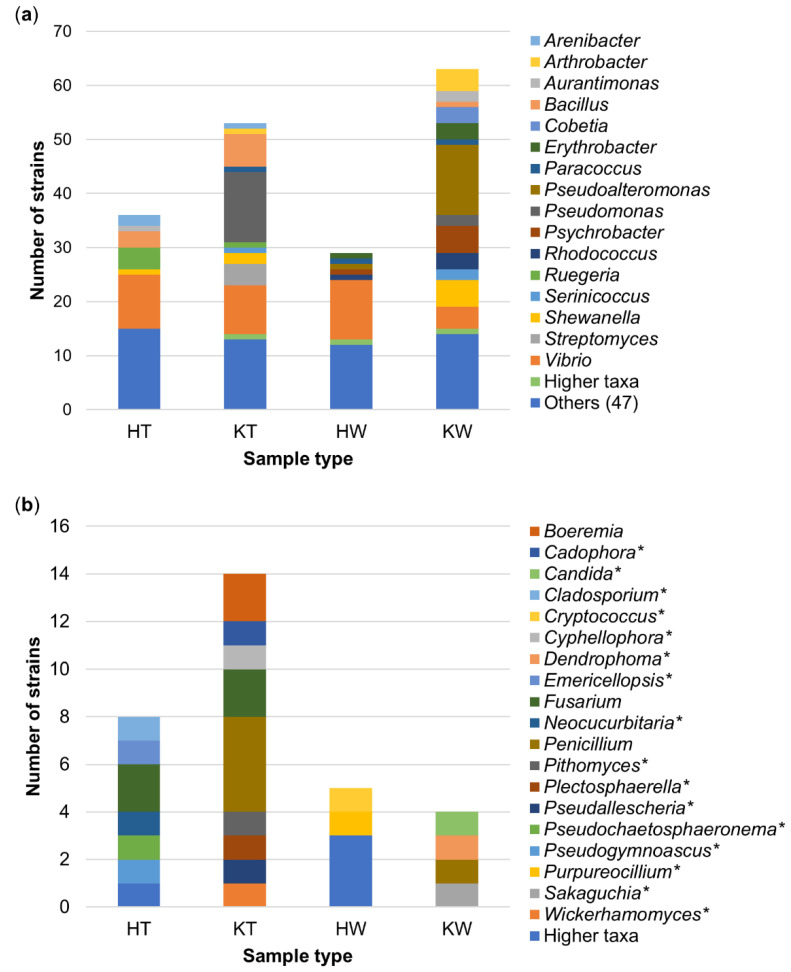
Taxonomic distribution of microorganism genera associated with the tunic of *C. intestinalis* and in seawater samples. Abundances of bacterial (**a**) and fungal (**b**) genera are given. Higher taxa: strain was not identified to a genus but to family or order level. Others: bacterial genera comprising ≤2 strains. * = fungal genera that were exclusive to one sample group. H, Helgoland; K, Kiel; T, Tunic; W, Seawater.

At genus level, only little overlap of tunic and seawater samples was observed (Figure 2b), since *Vibrio* was the only microbial genus that was identified in all samples (Figure 3). Tunic isolates showed 41 specific microbial genera (Figure 2b), among them the abundant bacterial genera *Arenibacter* (Flavobacteria; 3 isolates), *Ruegeria* (Alphaproteobacteria; 5 isolates), *Streptomyces* (Actinobacteria; 4 isolates), and the Sordariomycete fungus *Fusarium* sp. (4 isolates; Figure 3). Most bacterial tunic isolates were affiliated to the genera *Bacillus* (9 isolates), *Pseudomonas* (13 isolates; only detected in Kiel samples), and *Vibrio* (19 isolates; Figure 3a). *Vibrio* sp. was the predominant genus in seawater samples (15 isolates) along with the genera *Pseudoalteromonas* (14 isolates) and *Psychrobacter* (6 isolates). Seawater samples yielded also several exclusive bacterial genera, such as *Erythrobacter* (Alphaproteobacteria; 4 isolates), the gammaproteobacterial genera *Pseudoalteromonas* (14 isolates) and *Psychrobacter* (6 isolates) as well as the actinobacterial genus *Rhodococcus* (4 isolates). Fungi were much lesser abundant (22 isolates) in the tunic than bacteria (89 isolates). The isolated tunic-associated fungal community was dominated by *Fusarium* sp. (4 isolates) and *Penicillium* sp. (4 isolates; Figure 3b). Seawater samples yielded only nine fungal isolates of which five (*Candida* sp., *Cryptococcus* sp., *Dendrophoma* sp., *Purpureocillium* sp., and *Sakaguchia* sp.) were exclusive to the seawater samples.

### 3.2. Anticancer and Antimicrobial Activities of Bacterial and Fungal Extracts

**Figure 4:**
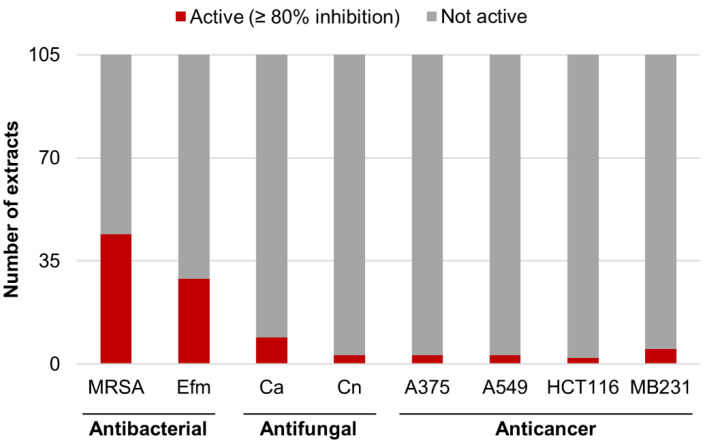
Bioactivities of microbial extracts (*n* = 105). Number of active extracts (≥80% inhibition at 100 µg/mL test concentration) in the categories antibacterial (MRSA: Methicillin-resistant *Staphylococcus aureus*, Efm: *Enterococcus faecium*), antifungal (Ca: *Candida albicans*, Cn: *Cryptococcus neoformans*) or anticancer activity (A375: Malignant melanoma cell line, A549: Lung carcinoma cell line, HCT116: Colon cancer cell line, MB231: Human breast cancer cell line).

In total, 105 microbial extracts from tunic-derived isolates were screened for their in vitro antimicrobial and anticancer activities. Nearly half of these extracts (45%) exhibited considerable bioactivity (≥80% inhibition at 100 µg/mL test concentration) in at least one bioassay (Table S3). Most of the microbial crude extracts showed antimicrobial activity (44%; Figure 4). None of the tested extracts had an inhibitory effect towards Gram-negative pathogens but many extracts were active against the Gram-positive bacteria methicillin-resistant *S. aureus* (MRSA) (*n* = 44) and *E. faecium* (*n* = 29). Of these, only nine extracts inhibited additionally the growth of *C. albicans* (inhibition between 88 and 100% at 100 µg/mL test concentration), i.e., crude extracts of the fungi *Fusarium* sp. (CKT84-CAG and CKT84-PDA), *Penicillium* sp. (CKT35-CAG and CKT35-PDA), *Penicillium brasilianum* (CKT49-PDA), the crude extract of the *Pithomyces chartarum* (CKT81-CAG and CKT81-PDA), and *Pyrenochaeta* sp. (CHT58-PDA) as well as the extract from the bacterium *Streptomyces* sp. (CKT43-GYM). Antifungal activity against *C. neoformans* was only detected in the *Pyrenochaeta* sp. extract CHT58-PDA and in the extracts of *Streptomyces* sp. strain CKT43 (CKT43-GYM and CKT43-MB; inhibition between 87 and 100% at 100 µg/mL test concentration). Only six extracts inhibited the proliferation of cancer cell lines (inhibition between 81 and 98% at 100 µg/mL test concentration). They belonged to *Boeremia exigua* (CKT91-CAG and CKT91-PDA), *Cadophora luteo-olivacea* (CKT85-CAG), *Emericellopsis maritima* (CHT37-PDA), *Pseudogymnoascus destructans* (CHT56-CAG), and *Streptomyces* sp. (CKT43-GYM). Notably, the fungal extract CHT56-CAG (*P. destructans*) showed selective activity against the breast cancer cell line MB231 (81% inhibition at 100 µg/mL test concentration; other tested cancer cell lines ≤20% inhibition at 100 µg/mL test concentration).

### 3.3. Extract Selection for Metabolomic Analyses and IC_50_ Determinations 

Due to the high number of extracts with bioactivity (*n* = 47), further prioritization steps were necessary to select the most promising candidates for in-depth chemical analyses. The first criterion we applied was a high bioactivity threshold (≥80% inhibitory activity at 100 μg/mL) selecting extracts with (1) antimicrobial (combined antibacterial and antifungal) or (2) anticancer or (3) both antimicrobial (antibacterial plus antifungal) and anticancer activity (Table S4). Hence, extracts showing (1) high antibacterial activity against the human pathogens MRSA and *E. faecium* plus high antifungal activity against at least one of the pathogenic yeasts (*C. albicans*, *C. neoformans*) or (2) high activity against at least one of the cancer cell lines (A375, A549, HCT116, MB231) or (3) a combination of both high antimicrobial and anticancer activity, were selected (Tables S3 and S4). This approach led to the selection of 12 extracts deriving from the fungi *E. maritima* (CHT37-PDA), *P. destructans* (CHT56-CAG), *Pyrenochaeta* sp. (CHT58-PDA), *Penicillium* sp. (CKT35-PDA), *P. brasilianum* (CKT49-PDA), *P. chartarum* (CKT81-CAG and CKT81-PDA), *Fusarium* sp. (CKT84-CAG and CKT84-PDA), *C. luteo-olivacea* (CKT85-CAG), *B. exigua* (CKT91-CAG and CKT91-PDA), and two bacterial extracts from *Streptomyces* sp. (CKT43-GYM and CKT43-MB).

**Figure 5:**
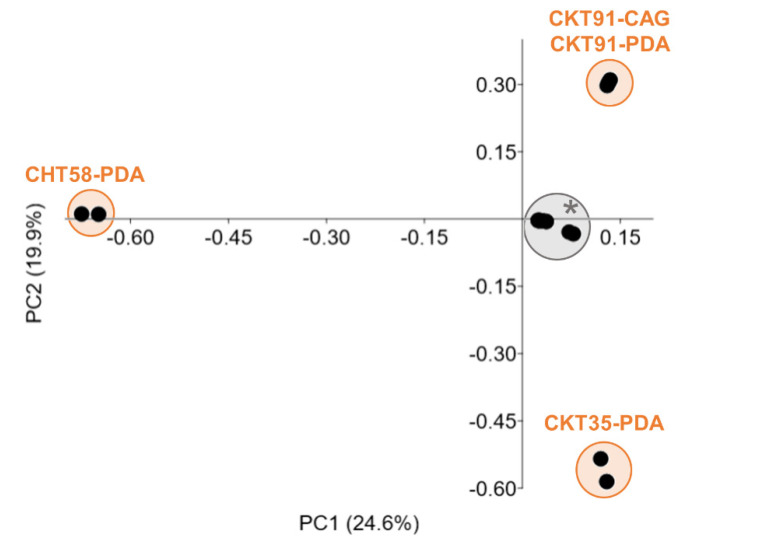
UPLC-MS/MS-based metabolite profiling of 12 pre-selected bioactive tunic-derived fungal extracts. The PCoA plot (Euclidean distance) was calculated using a pre-processed peak list based on UPLC-MS/MS data. *: cluster comprises the following extracts: CHT37-PDA, CHT56-CAG, CKT49-PDA, CKT81-CAG, CKT81-PDA, CKT84-CAG, CKT84-PDA, CKT85-CAG.

The second criterion for prioritization relied on the chemical distinctiveness of the fungal extracts, which was judged by a comparative LC-MS/MS-based metabolite profiling strategy. UPLC-MS/MS data of the 12 fungal extracts were pre-processed with MZmine 2. The automatically generated peak lists were statistically compared with regard to chemical diversity (number and intensity of peaks, *m/z* value and retention time of detected compounds) resulting in a PCoA plot (Figure 5). Four extracts, deriving from *Pyrenochaeta* sp. strain CHT58 (PDA medium), *Penicillium* sp. strain CKT35 (PDA medium), and the *B. exigua* isolate CKT91 (CAG and PDA media), showed a statistically different clustering from the remaining samples, indicating considerable chemical differences in their metabolomes (Figure 5; R: 0.78, *p*: 0.0001; Table S5). Notably, the crude extract of the fungus *Pyrenochaeta* sp. CHT58 (PDA medium) had the most different metabolome (R: 0.98, *p*: 0.007), reflected by the exceptionally high number of detected peaks (284 peaks, compared to 74–187 peaks in all other extracts; Table S1). These four extracts were prioritized and subjected to in-depth chemical investigations. 

Since *Streptomyces* sp. strain CKG43 (GYM and MB media) was the only bacterial extract showing considerable bioactivities (Table S3), it was also selected for in-depth metabolomic analyses. In addition, the extract of the ascomycete-type fungus *P. destructans* CHT56 grown on CAG medium was chosen for chemical analyses due to its selective anticancer activity against breast cancer cell line MB231 (Table S3). In summary, the bioactivity- and chemical diversity-based selection approach led to the prioritization of five fungal and two bacterial extracts for in-depth untargeted metabolomics analyses. 

Half maximal inhibitory concentrations (IC_50_) against the tested microbial pathogens and cancer cell lines were determined for all seven prioritized extracts (Table 1). *B. exigua* strain CKT91 grown on media CAG and PDA showed the lowest IC_50_ values against the tested cancer cell lines, e.g., lung cancer cell line A549 was inhibited with IC_50_ values of 4.3 and 5.4 µg/mL. Considerable antimicrobial activity was detected in all extracts (IC_50_ values between 1.4 and 74.8 µg/mL), except for the extract *B. exigua* CKT91-CAG. In particular, the *P. destructans* extract CHT56-CAG and the *Streptomyces* sp. extracts CKT43-GYM and CKT43-MB showed remarkable activity against MRSA with IC_50_ values between 6.1 µg/mL and 12 µg/mL. Moreover, *Streptomyces* sp. extract CKT43-GYM showed the strongest activity against *E. faecium* with an IC_50_ value of 5.1 µg/mL. The lowest IC_50_ values against the tested pathogenic yeasts *C. albicans* and *C. neoformans* were exhibited by the *Streptomyces* extracts CKT43-GYM and CKT43-MB, respectively, and the fungal extract CHT58-PDA (*Pyrenochaeta* sp.).

### 3.4. Metabolomic Analyses of Bioactive Tunic-Associated Microbial Strains

The metabolome of the seven prioritized crude extracts of *P. destructans* (CHT56-CAG), *Pyrenochaeta* sp. (CHT58-PDA), *Penicillium* sp. (CKT35-PDA), *Streptomyces* sp. (CKT43-GYM and CKT43-MB), and *B. exigua* (CKT91-CAG and CKT91-PDA) was investigated by state-of-the-art automated dereplication tools (FBMN, ISDB) combined with multiple databases (DNP, MarinLit, NP Atlas, Reaxys). Putative annotations of abundant compounds (i.e., compounds showing distinct peaks in the LC-MS chromatograms above the set minimum peak height) are shown in Supplementary Tables S6–S10 and in Figure S4. The respective annotated FBMNs are depicted in Supplementary Figures S5–S8. The analyzed chemical space of the seven selected microbial extracts comprised in total 22 different chemical families. Overall annotation rates varied between 24% (*Streptomyces* sp. strain CKT43) and 73% (*Penicillium* sp. strain CKT35). This highlights the strength of the dereplication strategy applied herein, as the annotation rates in untargeted metabolomics experiments are approximately 1.8% [57].

**Figure 6:**
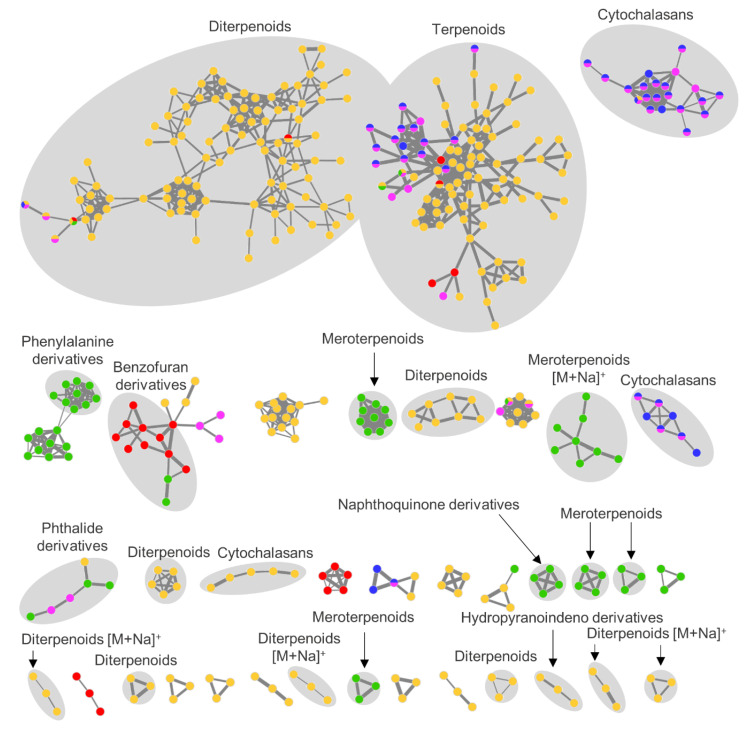
Global FBMN of five bioactive fungal extracts. The FBMN was constructed in GNPS with pre-processed MS/MS data. Molecular clusters containing at least three nodes are displayed and the width of edges corresponds to the respective cosine score. Putatively annotated clusters are highlighted in grey. Fungal extracts are color-coded as follows: red = *P. destructans* extract CHT56-CAG, yellow = *Pyrenochaeta* sp. extract CHT58-PDA, green = *Penicillium* sp. extract CKT35-PDA, blue = *B. exigua* extract CKT91-CAG, pink = CKT91-PDA. Putatively annotated compounds are listed in Tables S6–S9 in the Supplementary Materials.

The global FBMN of the five selected fungal extracts consisted of 817 nodes, of which 394 fell into 36 clusters containing at least three nodes (Figure 6). Many clusters (70%) were putatively annotated to various NP classes, such as alkaloids (cytochalasans and phenylalanine derivatives), polyketides (benzofuran, hydropyranoindeno, napthoquinone and phthalide derivatives), and terpenoids (di- and meroterpenoids). Most molecular clusters were produced by only one fungal extract, but two clusters, putatively identified as terpenoids, were represented by several nodes from all five fungal extracts.

The *Pyrenochaeta* sp. strain CHT58 (PDA medium) showed an extraordinarily high chemical diversity (284 nodes; Figure 6 and Figure S5, Table S6). Most compounds were putatively identified as diterpenoids, such as the aphidicolins (**5**,**7–10**,**12–14**,**16–18**,**20**,**24**,**27**,**29**,**32**,**34**,**41**,**46**). Furthermore, the macrolide talarodilactone B (**28**), the cytochalasan alkaloid periconiasin I (**23**), a pyrrolizidine alkaloid (**30**) and some polyketides (**2**,**6**,**47**) were putatively annotated. No match to any known compound was found for 18 abundant compounds (**1**,**3**,**4**,**11**,**21**,**22**,**25**,**33**,**35–40**,**43**,**44**,**48**,**49**; Table S6) in any of the databases used, hence they may represent compounds not described in the literature.

The *P. destructans* isolate cultivated on medium CAG (CHT56-CAG) produced compounds of polyketide (**55**,**60–62**) and terpenoid (**56**,**65**,**67**,**70**) origin (Figure 6 and Figure S6, Table S7). Accordingly, the two largest clusters in the FBMN, which consisted in total of 78 nodes, were putatively assigned to benzofuran-type polyketides and sesquiterpenoids. Dereplicated terpenoids belonged to the chemical families of di-(**70**), mero- (**65**,**67**) and sesquiterpenoids (**56**), isolated from marine-derived fungi from the orders of Eurotiales, Hypocreales, and Pleosporales. Sixteen abundant compounds (**52–54**,**57–59**,**63**,**64**,**66**,**68**,**69**,**71–75**) and two clusters in the MN could not be annotated to any known metabolite and may therefore represent new compounds.

The antimicrobial extract of *Penicillium* sp. (CKT35-PDA) contained a broad chemical diversity with six different putatively identified chemical families (Figure 6 and Figure S7, Table S8). Its FBMN profile was dominated by one large cluster, which contained the benzofuran derivatives penibenzone C (**77**), penicifuran C (**79**), and D (**82**) as well as mycophenolic acid and its methyl ester derivative (**85**, **90**). In addition, the naphthoquinone derivative flaviolin (**76**), the phenylalanine derivative asperphenamate (**96**) and its analog B (**89**), the quinolone alkaloid quinolactacin A (**81**), and several meroterpenoids (**83**,**84**,**86**,**87**,**92**,**93**) were putatively identified from extract CKT35-PDA. However, six abundant compounds (**78**,**88**,**91**,**94**,**95**,**97**) and three molecular clusters in the FBMN could not be annotated to any known NP classes.

The metabolome of the tunic-associated fungus *B. exigua* strain CKT91 (former scientific name: *Phoma exigua*) cultivated on the media CAG and PDA was dominated by the PKS-NRPS hybrid family of cytochalasans (**100–107**,**110**; Figure 6 and Figure S8, Table S9), which were detected in crude extracts from both culture media. The three largest clusters in the FBMN were putatively annotated to this chemical family, which is well known from *Phoma* spp. For instance, deoxaphomin C (**107**) and proxiphomin (**110**) were putatively identified compounds in this family. Moderate to weak antibacterial activity was observed in extract CKT91-PDA and interestingly, this extract contained more specific nodes (*n* = 25) in the FBMN than CKT91-CAG (*n* = 16). Two compounds specific to medium PDA were putatively identified as cytochalasin Z_11_ (**105**) and the ergosterol-type steroid dankasterone B (**109**).

**Figure 7:**
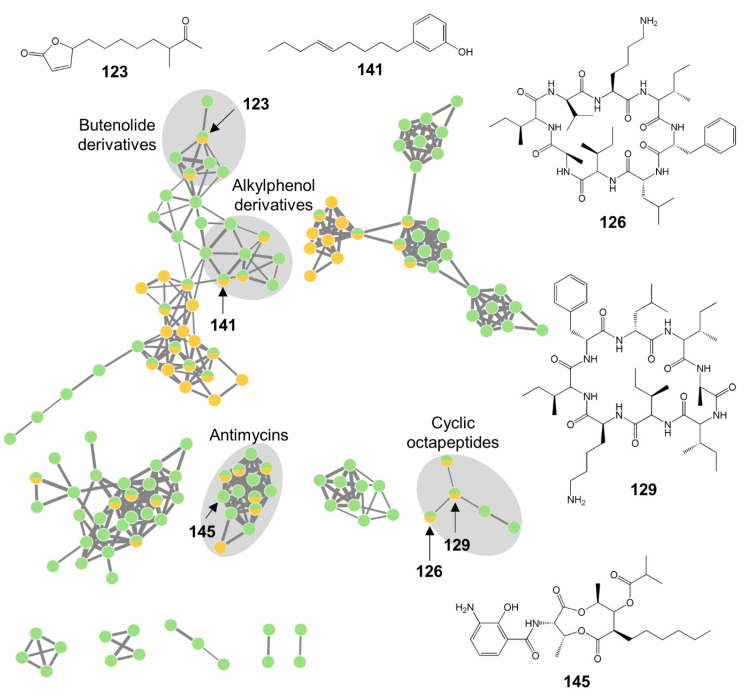
The global metabolome of *Streptomyces* sp. strain CKT43. The annotated global FBMN was constructed in GNPS with pre-processed MS/MS data. Single nodes are not displayed and the width of edges corresponds to the respective cosine score. Putatively annotated clusters are highlighted in grey and putatively identified compounds are annotated in the MN (for identification see Table S10). Nodes are color-coded by the respective cultivation medium: yellow = GYM, green = MB.

The extracts from *Streptomyces* sp. strain CKT43, CKT43-GYM, and CKT43-MB, showed a diverse metabolome, with 187 nodes in the FBMN and two different NP classes (Figure 7, Table S10). The alkylphenol anaephene A (**141**), the deformylated antimycin derivative A1a (**146**), the butenolide MKN-003A (**123**), and surugamides (**126–129**) were detected in extracts derived from both cultivation media (GYM, MB). The *Streptomyces* sp. crude extract CKT43-MB accounted for the majority of nodes in the FBMN and contained 15 unique compounds (**112**,**116**,**117**,**119**,**122**,**124**,**125**,**130–133**,**135–137**,**145**) whereas the fermentation of this strain on medium GYM yielded only seven unique compounds (**118**,**121**,**134**,**142**,**144**,**147**,**148**; Table S10). Notably, only extract CKT43-GYM showed anticancer activities, but none of its unique compounds was annotated to a known NP. Moreover, several unknown clusters in the FBMN were not assigned to any known compound and remain potentially new.

In summary, the prioritized extracts derived from diverse microorganisms showed differential and diverse metabolomes. Two to seven chemical families were putatively annotated in the seven extracts from five microbial strains. In particular, *P. destructans* strain CHT56 and *Pyrenochaeta* sp. strain CHT58 promise to be versatile MNPs producing strains, due to the high diversity of putatively annotated chemical families (Tables S6 and S7).

## 4. Discussion

This study aimed to assess the biotechnological potential of the culturable microbial community associated with the tunic of the solitary ascidian *C. intestinalis.* Therefore, ascidians were sampled at two collection sites with different salinity levels. Isolation efforts yielded 89 bacterial and 22 fungal tunic-associated strains affiliated to 51 microbial genera (Figure 1, Figure 3 and Figure S1, Table S2). As expected [9,30], tunic-derived isolates differed from the surrounding seawater (Figure 1, Figure 2 and Figure 3, Figures S2 and S3). The comparably higher abundance of tunic-associated Alphaproteobacteria, such as the tunic-specific Rhodobacteraceae *Ruegeria, Leisingera* and *Litoreibacter*, Flavobacteria (e.g., *Arenibacter*) and Firmicutes (mainly *Bacillus*) is in accordance with reports on the culture-dependent [58,59] or -independent [9,30,60] microbiome of *C. intestinalis* and other ascidian species. In particular, Rhodobacteraceae and the ubiquitous *Bacillus* sp. are common associates of marine invertebrates [60,61]. Notably, several bacterial taxa isolated from the tunic, e.g., *Bacillus* sp., are mobile. Although mobility is not necessary when being associated with the ascidian’s tunic, one way of active movement described from *Bacillus* sp. is swarming on solid surfaces as a response to various environmental cues [62,63]. Moreover, it is likely that several tunic-associated bacteria were recruited from the seawater [60,64], where flagella enable their movement in the water column [62,63]. Tunic- and seawater-derived isolates also differed between the sampling sites. This finding can be attributed to the different environmental conditions of the Baltic and North Sea, as Kiel Fjord in the Baltic is characterized by brackish water (~18 psu), whereas seawater around Helgoland island (which is in ca. 50 km distance to mainland Germany) in the North Sea has oceanic salinity (~30 psu). The high abundance of some bacterial genera such as *Pseudomonas* in Baltic samples may be attributed to the lower salt tolerance described for some strains affiliated to these genera (e.g., [65]). In addition, the higher microbial diversity of Kiel Fjord samples may be attributed to the fact that Kiel Fjord is an area with high anthropogenic impact featuring several harbors, the highly frequented Kiel Canal (ship traffic) and industry, while Helgoland is an offshore island with substantially lower anthropogenic input. Despite the observed differences between the sampling sites and sample types, culture-dependent studies usually capture only 0.001–1% of the actual microbial diversity of a habitat, a phenomenon well-known as the “great plate count anomaly” [66,67]. Moreover, isolation of microorganisms is usually biased towards easily culturable, fast growing microorganisms and therefore, does not necessarily reflect the complete microbial diversity of the explored environmental sample [66,67]. Out of 37 bacterial genera isolated from the tunic of *C. intestinalis*, only *Arenibacter* and *Kiloniella* were previously isolated from the same source [9]. Previous culture-independent studies [9,30] on the bacterial diversity of the tunic of *C. intestinalis* identified sequences affiliated to the four bacterial phyla that were also detected in this study (Actinobacteria, Bacteroidetes, Firmicutes, Proteobacteria). Although fungi were reported from other ascidians [68], we provide here the first evidence for fungi associated with the tunic of *C. intestinalis*. In combination, these results indicate that *C. intestinalis* hosts a diverse and specific culture-dependent microbiota associated with its tunic. This is in line with previous results presenting a further evidence that ascidians are a rich source of microorganisms [6,16,68]. 

The in vitro screening effort performed in this study revealed a high number of bioactive crude extracts (45%), pointing out the exceptional potential of the tunic-associated microbiota of *C. intestinalis* for pharmaceutical applications (Figure 4, Table S3,). Most of the active extracts inhibited the Gram-positive pathogens MRSA (94%) and *E. faecium* (62%), but only few showed anticancer activity (13%). This contrasts with a recent review that analyzed bioactive ascidian-derived microbial compounds that showed higher rates of cytotoxicity (47%) than antimicrobial activity (31%) [16]. However, the vast potential of marine-derived microorganisms for discovery of novel antibiotics from ascidians and other marine invertebrates is known [69,70,71]. The fact that pathogenic Gram-negative bacteria were not inhibited by any of our tunic-associated microorganisms is in line with their general lesser susceptibility towards antibiotics due to their additional outer membrane [70,72]. 

The only microorganism isolated from the tunic of European *C. intestinalis* so far, *Pseudoalteromonas tunicata*, shows a variety of bioactivities such as antibacterial and larval toxicity preventing micro- and macrofouling on the ascidian’s tunic [32,73]. For *C. intestinalis,* the allocation of defensive compounds on the tunic is crucial, since the tunic lacks other physical defense strategies, such as spicules or accumulation of acid or vanadium [74,75]. Although potential chemical defense functions of the screened tunic-associated microbiota cannot be clarified within the scope of this study, the high number of bioactive extracts detected in this study is in line with numerous reports of ascidian-derived microorganisms producing novel MNPs with various pharmaceutical properties [1,6,16].

In order to prioritize the most promising extracts for in-depth metabolomic analyses out of the 47 bioactive extracts in total, two selection criteria were applied, (i) an 80% bioactivity threshold (at 100 µg/mL test concentration) for anticancer or antimicrobial (antibacterial and antifungal) activity, and (ii) chemical distinctiveness based on a statistical comparison of metabolite profiles. Application of these selection criteria resulted in the prioritization of four extracts derived from three tunic-associated fungal strains (*Pyrenochaeta* sp. extract CHT58-PDA, *Penicillium* sp. extract CKT35-PDA, *B. exigua* extracts CKT91-CAG and -PDA), and two extracts from a *Streptomyces* sp. bacterium (CKT43-GYM and -MB). Additionally, one fungal extract (*P. destructans* extract CHT56-CAG) was selected for further chemical investigations because of its selective anticancer activity.

The fungal genera *Pseudogymnoascus* and *Pyrenochaeta* are relatively rare in marine habitats but were previously isolated from a few marine invertebrates [76,77]. Diterpenoids dominated the metabolome of the crude extract of *Pyrenochaeta* sp. (CHT58-PDA) with two big molecular clusters in the FBMN (Figure S5, Table S6). Diterpenoids frequently form sodium adducts during ionization (e.g., [78]) explaining the appearance of a large cluster of sodiated diterpenoids in the network (Figure S5). These diterpenoids were previously reported from other members of the fungal class Dothideomycetes (*Pyrenochaeta* is affiliated to this class) but also from various other fungal taxa (Eurotiomycetes, Leotiomycetes, Sordariomycetes) underlining their ubiquitous distribution in terrestrial and marine fungi [79]. Surprisingly, the PDA extract of the underexplored *Pyrenochaeta* sp. showed by far the most diverse metabolome (Figure 6 and Figure S5, Tables S1 and S7) reflected by the highest number of detected metabolites. None of the putatively annotated compounds in this extract has reported activities against *C. albicans* or *C. neoformans*, although the extract showed strong antifungal activity (Table 1). This may suggest that one or several of the putatively novel metabolites (**1**,**3**,**4**,**11**,**21**,**22**,**25**,**33**,**35–40**,**43**,**44**,**48**,**49**; Table S6) are responsible for the detected antifungal activity. The moderate antibacterial activity of this extract may be due to the putatively identified anthraquinone derivative 10-deoxybostrycin (**6**) as well as the pyrrolizidine alkaloid CJ-16,264 (**30**), both of which have been reported to display antibacterial activities against *S. aureus* [20,80].

Among the putatively identified compounds detected in the CAG extract of *P. destructans* strain CHT56, the polyketides phialofurone (**60**) and 3,4-dihydro-6-methoxy-8-hydroxy-3,4,5-trimethyl-isocoumarin-7-carboxylic acid methyl ester (**61**) and the diterpenoid (9ξ,13α)-6,9-dihydroxypimara-5,8(14),15-trien-7-one (**70**) are reportedly cytotoxic against several human cancer cell lines [81,82,83]. Hence, these putatively identified compounds may be responsible for the detected moderate anticancer activity, which is being observed for the first time in a *Pseudogymnoascus* sp. extract. However, none of the putatively identified compounds of the CHT56-CGA extract is known for activity against MRSA, *E. faecium* or the cancer cell line MB231.

The largely unexplored fungal strains CHT56 (*P. destructans*) and CHT58 (*Pyrenochaeta* sp.) thus emerge as a promising source for the discovery of (novel) bioactive MNPs. Observed antibacterial or antifungal activities of extracts from both strains were not explained by the putatively identified compounds. Finally, both extracts contained several compounds and molecular clusters that could not be linked to any known compounds neither by automated nor manual dereplication using multiple pipelines and databases. These extracts deserve attention in future chemical isolation studies, as the unidentified metabolites may represent new bioactive MNPs.

The remaining five selected bioactive extracts were derived from strains affiliated to extensively studied microbial taxa, *B. exigua* (formerly *P. exigua*, strain CKT91), *Penicillium* (strain CKT35) and *Streptomyces* (strain CKT43), all of which are prolific producers of MNPs [79,84,85,86] with hundreds (*Phoma*: 378) to thousands of described NPs (*Penicillium*: 2634, *Streptomyces*: 8769; number of isolated NPs retrieved from DNP on 04.09.2020). *Penicillium, Phoma* (former taxonomic classification of strain CKT91, *B. exigua*) and *Streptomyces* spp. are facultative marine microorganisms frequently isolated from various marine environments [16,85,87], including Kiel Fjord habitats [88,89].

*Penicillium* is among the best studied and richest fungal genera with enormous metabolic capacity to produce diverse types of pharmaceutically relevant metabolites with antibiotic, anticancer and anti-inflammatory activities (e.g., [90,91]). Interestingly, the selected *Penicillium* sp. extract CKT35-PDA showed a completely different chemical profile compared to that of strain *Penicillium brasilianum* CKT49 grown in the same medium (Figure 5). Species- and even strain-specific metabolomes have been demonstrated for *Penicillium* spp. For example, the putatively identified compounds andrastin A (**92**) and 4′-hydroxy-mycophenolic acid (**83**) were previously described as chemotaxonomic markers for *Penicillium* spp. or strains [92,93]. Notably, one third of the putatively identified compounds from the *Penicillium* sp. CKT35-PDA extract were also detected in three sea foam-derived *Penicillium* spp. strains analyzed in our previous study [93]. The dereplicated polyketides penicifuran C and D (**77**,**79**), the meroterpenoid mycophenolic acid (**85**), and the phenylalanine derivative asperphenamate (**96**) reportedly inhibit the growth of *Staphylococcus* spp. [94,95,96]. Antifungal activity against *C. albicans* and *C. neoformans* has never been reported for any putatively annotated compound in this extract. Moreover, six compounds (**78**,**88**,**91**,**94**,**95**,**97**; Table S8) and some clusters in the *Penicillium* sp. CKT35 FBMN (Figure S7) remain unannotated and may represent novel metabolites.

The *B. exigua* (formerly *P. exigua*) extracts CKT91-CAG and -PDA were clearly dominated by cytochalasans, hybrids of polyketides and amino acids (Figure 6 and Figure S8, Table S9). Cytochalasans are a large chemical family produced by several fungal taxa with various bioactivities such as antimicrobial, antiparasitic, antiviral, and cytotoxic activities [97,98]. Several of the putatively identified cytochalasans (**100**,**102–104**,**106**,**107**,**110**) have been reported to inhibit the proliferation of lung carcinoma cell line A549, which may underlie the strong anticancer bioactivities observed in both *B. exigua* extracts [97,99]. However, neither the putatively annotated cytochalasans nor the sterol dankasterone B (**110**) have reported antimicrobial activities.

Dereplication of the GYM and MB extracts of *Streptomyces* sp. (CKT43) generated the lowest annotation rate (24%; Figure 7, Table S10) suggesting a highly unexplored chemical space. *Streptomyces* spp. are a very prolific source of novel bacterial MNPs (for example marine-derived *Streptomyces* spp. yielded 167 novel metabolites in 2018 [79]), and they still remain a treasure trove for biodiscovery of new MNPs. According to our literature survey, no antimicrobial activity has been reported from the putatively annotated compounds. Notably, anticancer activity was only detected in CKT43-CAG extract but none of the specific compounds for this extract could be annotated to a known NP. Only the putatively identified deformylated antimycins (**160,161**), detected in both extracts, inhibit the proliferation of HeLa cells [100]. It remains to be proven whether the putatively novel compounds detected in CKT43-CAG (**118**,**121**,**134**,**142**,**144**,**147**,**148**; Table S10) are responsible for the anticancer activities of this extract.

The five prioritized strains isolated from the tunic of *C. intestinalis* appear to produce unknown chemical scaffolds with potential bioactivities for the discovery of novel anticancer or antimicrobial lead compounds. All extracts deserve further scientific attention with regard to isolation and characterization of their putatively novel and bioactive constituents. Particularly the *Streptomyces* sp. isolate CKT43 is promising for in-depth chemical studies, since the majority of compounds could not be matched to any known compound in multiple databases and *Streptomyces* spp. are some of the most prolific producers of antibiotics and anticancer drugs [3,79,84].

In summary, the present study identified a diverse culturable microbiome associated with the tunic of *C. intestinalis* that differed from the ambient seawater, but also between the two sampling sites. To our knowledge, this is the first report of fungi being associated with the tunic of *C. intestinalis*. The isolated tunic microbiota appeared as a highly rich reservoir of MNPs with antimicrobial and cytotoxic activities. Untargeted metabolomics studies on seven selected extracts indicated a high chemical diversity with compounds putatively assigned to alkaloids, lipids, peptides, polyketides, and terpenoids. However, many detected metabolites could not be annotated to any known NP and may therefore be new. Their chemical structure and bioactivity profiles need to be verified in future scale-up studies following their purification and structure elucidation. Hence, this study suggests that the so far unexplored tunic-associated microbiota of *C. intestinalis* from Helgoland and Kiel Fjord may be an excellent resource for replenishing the MNPs discovery pipeline with novel bioactive compounds.

## Figures and Tables

**Table 1:** Antimicrobial and anticancer activities of selected extracts. IC_50_ values are expressed in µg/mL. MRSA: Methicillin-resistant *Staphylococcus aureus*, Efm: *Enterococcus faecium*, Ca: *Candida albicans*, Cn: *Cryptococcus neoformans*, A375: Malignant melanoma, A549: Lung carcinoma, HCT116: Colon cancer, MB231: Breast cancer. Positive controls: Chloramphenicol (MRSA), ampicillin (Efm), nystatin (Ca), amphotericin (Cn), doxorubicin (A375, A549, HCT116, MB231).

Extract Code	Taxonomic Classification	MRSA	Efm	Ca	Cn	A375	A549	HCT116	MB231
CHT56-CAG	*P. destructans*	6.1	16.7	>100	>100	>100	>100	>100	51.2
CHT58-PDA	*Pyrenochaeta* sp.	35.4	17	12	14	>100	>100	>100	>100
CKT35-PDA	*Penicillium* sp.	74.8	38	21	51	>100	>100	>100	>100
CKT91-CAG	*B. exigua*	>100	>100	>100	>100	42.4	4.3	29.8	8.3
CKT91-PDA	*B. exigua*	19.8	67	>100	>100	37.6	5.4	23.0	7.8
CKT43-GYM	*Streptomyces* sp.	12	5.1	7.1	6.8	26.1	31.9	30.9	40.9
CKT43-MB	*Streptomyces* sp.	9.3	20	12	1.4	>100	>100	>100	>100
Positive control		1.2	2.4	7.2	0.1	0.4	16.3	33.1	7.9

## Supplementary Materials

The following are available online at https://www.mdpi.com/2076-2607/8/11/1732/s1, Figure S1: Number of microbial strains isolated from the tunic of *C. intestinalis* and seawater reference, Figure S2: Distribution of bacterial orders across the sample types and their geographic locations, Figure S3: Distribution of fungal orders across the sample types and their geographic locations, Figure S4: Chemical structures of putatively identified compounds in crude extracts of five selected candidate strains isolated from the tunic of *C. intestinalis*, Figure S5: FBMN of the crude extract of *Pyrenochaeta* sp. strain CHT58 cultivated on PDA medium, Figure S6: FBMN of the crude extract of *Pseudogymnoascus destructans* strain CHT56 cultivated on CAG medium, Figure S7: FBMN of the crude extract of *Penicillium* sp. strain CKT35 cultivated on PDA medium, Figure S8: FBMN of the crude extracts of *Boeremia exigua* strain CKT91 cultivated on CAG (blue nodes) and PDA (red nodes) media, Table S1: Parameters for MZmine-processing of UPLC-MS/MS data, Table S2: Identification of microbial strains isolated from *C. intestinalis* and seawater reference in Helgoland and Kiel Fjord, Table S3: Bioactivity (%) of crude extracts derived from tunic-associated microbial strains at a test concentration of 100 µg/mL, Table S4: Bioactivity-based selection criterion for the prioritization of extracts for in-depth chemical analyses, Table S5: ANOSIM comparison of chemically different extracts, Table S6: Putative annotation of metabolites detected in the crude extract of *Pyrenochaeta* sp. strain CHT58 cultivated on PDA medium, Table S7: Putative annotation of metabolites detected in the crude extract of *Pseudogymnoascus destructans* strain CHT56 cultivated on CAG medium, Table S8: Putative annotation of metabolites detected in the crude extract of *Penicillium* sp. strain CKT35 cultivated on PDA medium, Table S9: Putative annotation of metabolites detected in the crude extracts of *Boeremia exigua* strain CKT91 cultivated on CAG and PDA media, Table S10: Putative annotation of metabolites detected in the crude extracts of *Streptomyces* sp. strain CKT43 cultivated on GYM and MB media.
