# Supplementary material for: Culture-Dependent Microbiome of the Ciona intestinalis Tunic: Isolation, Bioactivity Profiling and Untargeted Metabolomics
Source: Microorganisms. 2020 Nov 5;8(11):1732. doi: 10.3390/microorganisms8111732 (PMC7694362; doi:10.3390/microorganisms8111732)
Supplement: Supplementary file 1 [file microorganisms-08-01732-s001.pdf]

*Supplementary Information*

# Culture-dependent microbiome of the *Ciona intestinalis* tunic: Isolation, bioactivity profiling and untargeted metabolomics

Caroline Utermann [1], Vivien A. Echelmeyer [1], Martina Blümel [1], Deniz Tasdemir [1,2*]

[1] GEOMAR Centre for Marine Biotechnology (GEOMAR-Biotech), Research Unit Marine Natural Products Chemistry, GEOMAR Helmholtz Centre for Ocean Research Kiel, Am Kiel-Kanal 44, 24106 Kiel, Germany

[2] Kiel University, Christian-Albrechts-Platz 4, Kiel 24118, Germany

* Corresponding author: Deniz Tasdemir

Email: dtasdemir@geomar.de

**This document includes:**

**Supplementary Figures S1-S8**

Figure S1. Number of microbial strains isolated from the tunic of *C. intestinalis* and seawater reference.

Figure S2. Distribution of bacterial orders across the sample types and their geographic locations.

Figure S3. Distribution of fungal orders across the sample types and their geographic locations.

Figure S4. Chemical structures of putatively identified compounds in the crude extracts of five selected microbial strains isolated from the tunic of *C. intestinalis*.

Figure S5. FBMN of the crude extract of *Pyrenochaeta* sp. strain CHT58 cultivated on PDA medium.

Figure S6. FBMN of the crude extract of *Pseudogymnoascus destructans* strain CHT56 cultivated on CAG medium.

Figure S7. FBMN of the crude extract of *Penicillium* sp. strain CKT35 cultivated on medium PDA.

Figure S8. FBMN of the crude extract of *Boeremia exigua* strain CKT91 cultivated on CAG (blue nodes) and PDA (red nodes) media.

**Supplementary Tables S1-S10**

Table S1. Parameters for MZmine-processing of UPLC-MS/MS data.

Table S2. Identification of microbial strains isolated from *C. intestinalis* and seawater reference in Helgoland and Kiel Fjord.

Table S3. Bioactivity (%) of crude extracts derived from tunic-associated microbial strains at a test concentration of 100 µg/mL.

Table S4. Bioactivity-based selection criterion for the prioritization of extracts for in-depth chemical analyses.

Table S5. ANOSIM comparison of chemically different extracts.

Table S6. Putative annotation of metabolites detected in the crude extract of *Pyrenochaeta* sp. strain CHT58 cultivated on PDA medium.

Table S7. Putative annotation of metabolites detected in the crude extract of *Pseudogymnoascus destructans* strain CHT56 cultivated on CAG medium.

Table S8. Putative annotation of metabolites detected in the crude extract of *Penicillium* sp. strain CKT35 cultivated on PDA medium.

Table S9. Putative annotation of metabolites detected in the crude extracts of *Boeremia exigua* strain CKT91 cultivated on CAG and PDA media.

Table S10. Putative annotation of metabolites detected in the crude extracts of *Streptomyces* sp. strain CKT43 cultivated on GYM and MB media.

**Supplementary References 1-45**

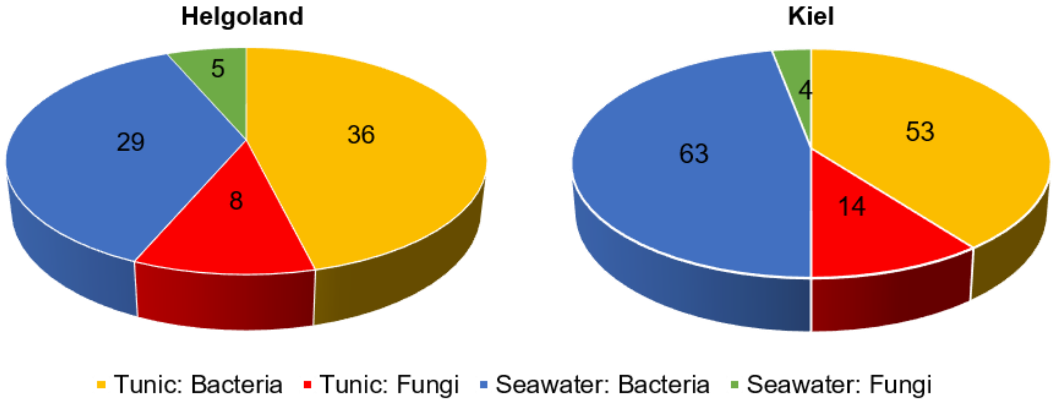


**Figure S1. Number of microbial strains isolated from the tunic of *C. intestinalis* and seawater reference.** Left: number of the isolates from Helgoland samples, right: number of the isolates from Kiel samples.

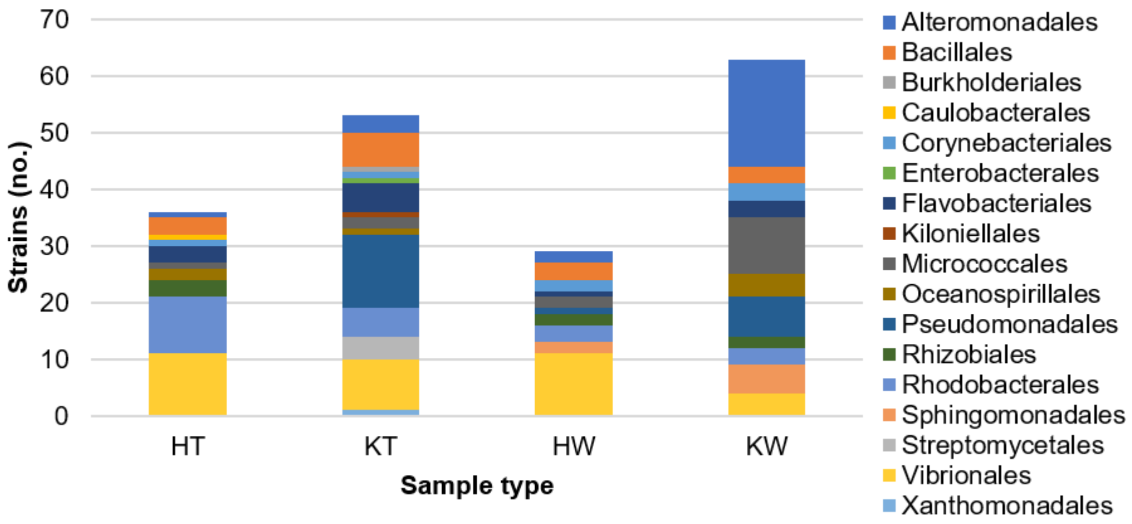


**Figure S2. Distribution of bacterial orders across the sample types and their geographic locations.** Sample types are abbreviated as: HT: Helgoland, tunic; KT: Kiel, tunic; HW: Helgoland, seawater; KW: Kiel, seawater.

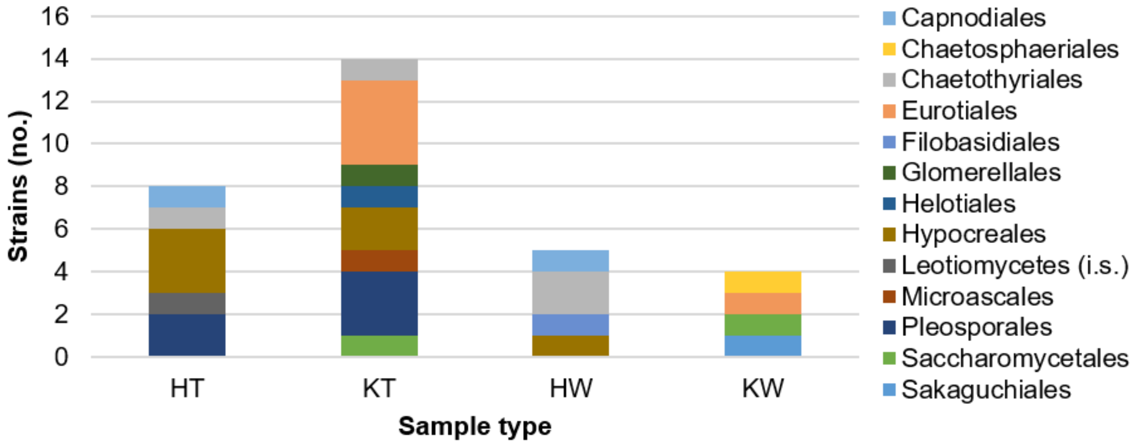


**Figure S3. Distribution of fungal orders across the sample types and their geographic locations.** Sample types are abbreviated as: HT: Helgoland, tunic; KT: Kiel, tunic; HW: Helgoland, seawater; KW: Kiel, seawater. i.s. = *incertae sedis* (taxonomic placement of order uncertain).

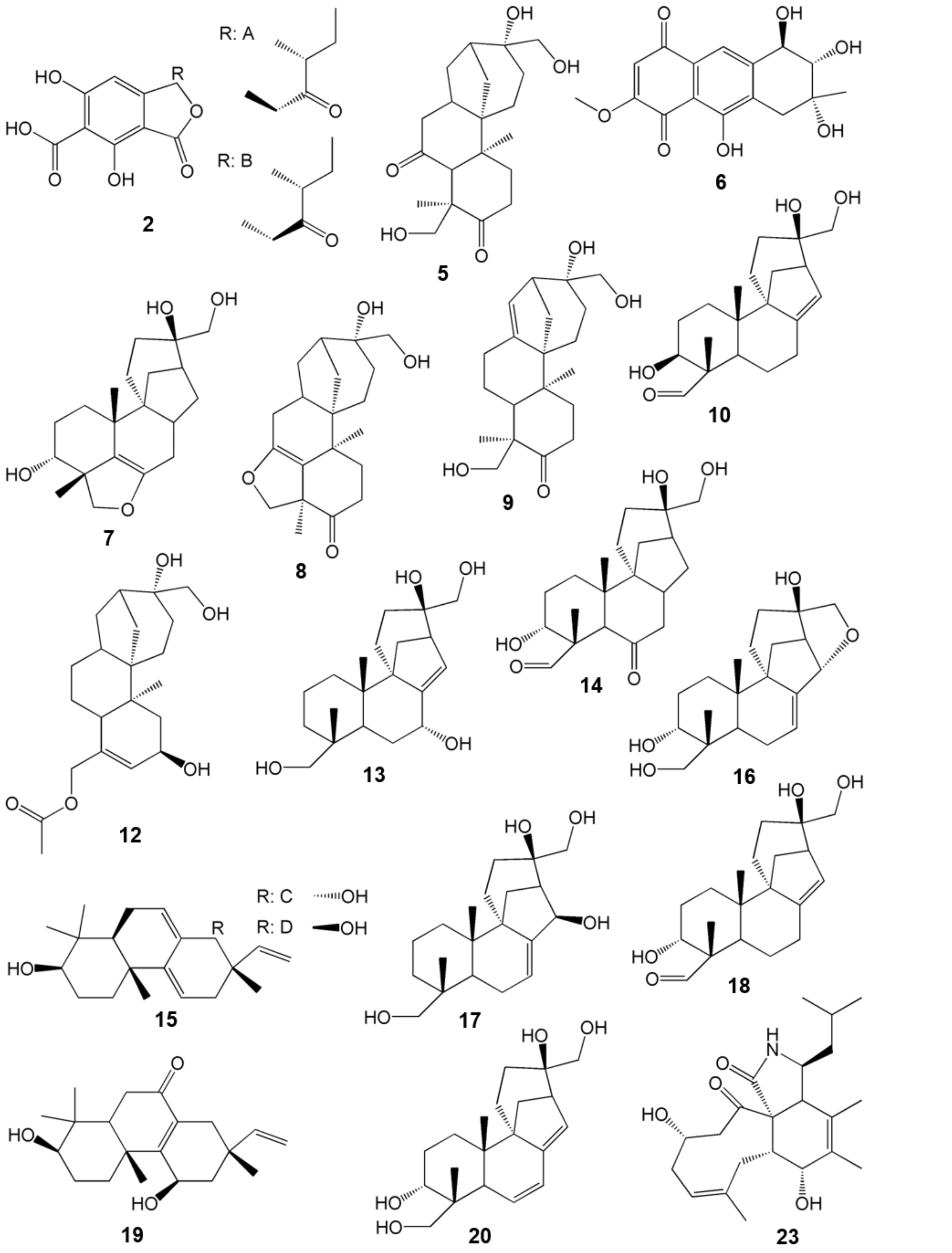


**Figure S4. Chemical structures of putatively identified compounds in the crude extracts of five selected microbial strains isolated from the tunic of *C. intestinalis*.** Structures are given with their respective peak number (see Tables S6-S10). The following compounds are shown in Figure 7 in the original publication: **123**, **126**, **129**, **141** and **145**.

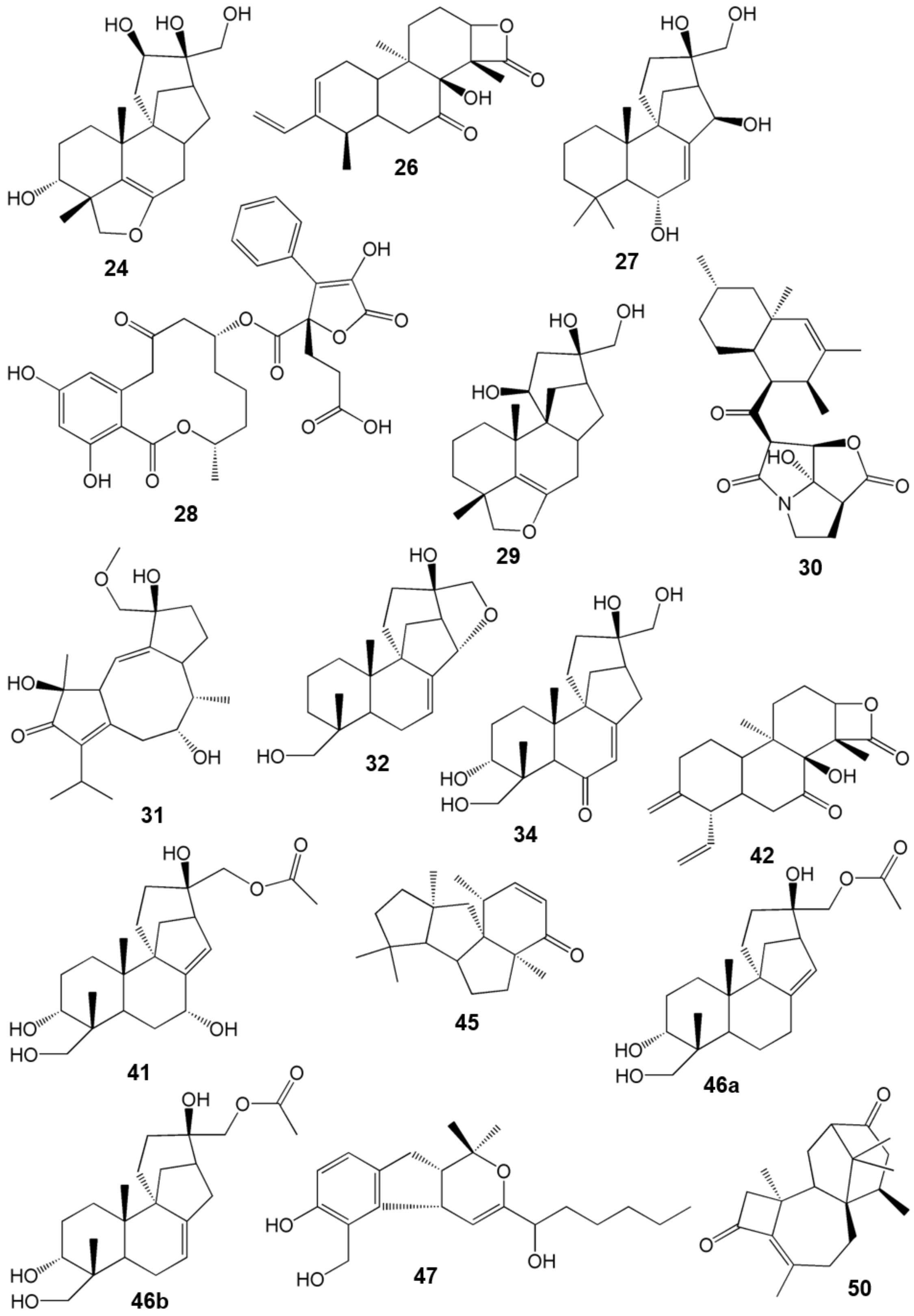


**Figure S4. (continued)**

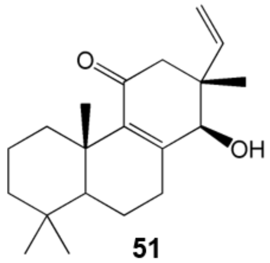

**51**

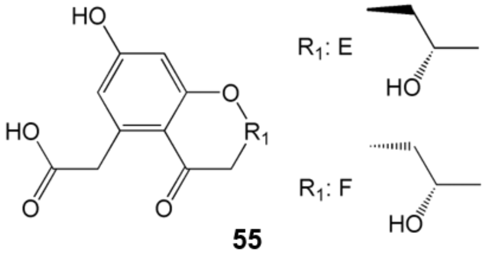

**55**

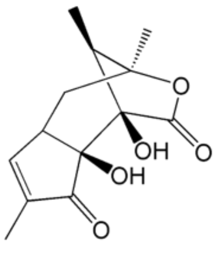

**56**

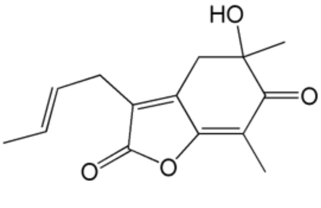

**60**

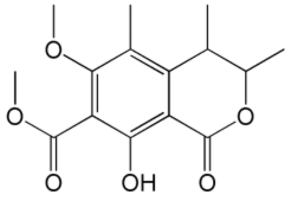

**61**

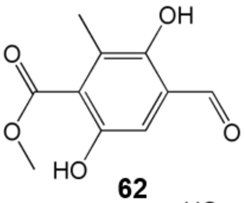

**62**

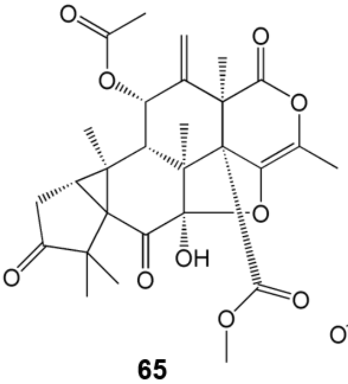

**65**

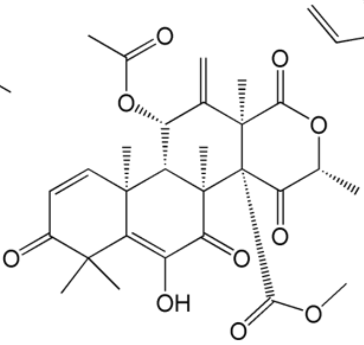

**67**

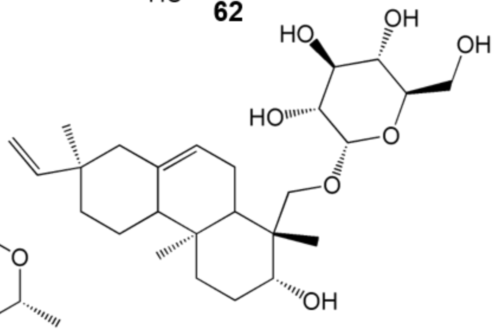

**70**

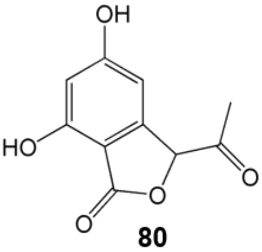

**80**

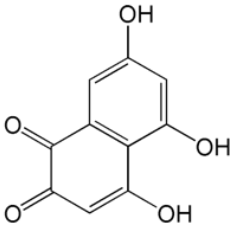

**76**

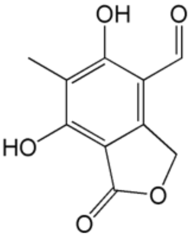

**77**

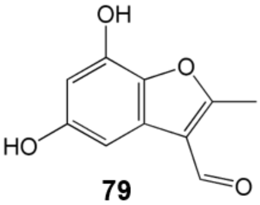

**79**

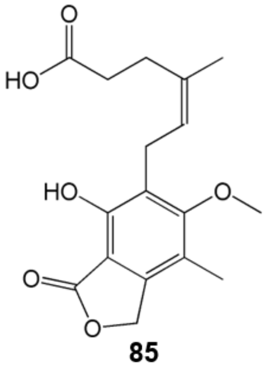

**85**

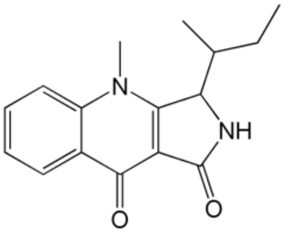

**81**

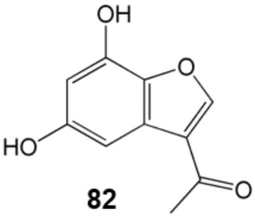

**82**

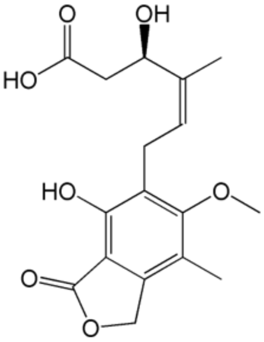

**83**

**Figure S4. (continued)**

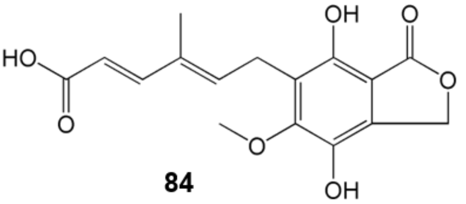

**84**

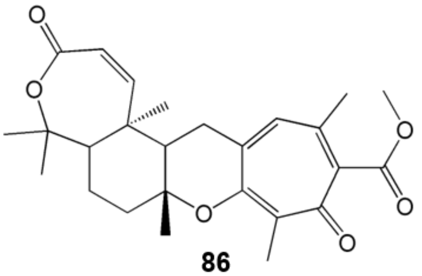

**86**

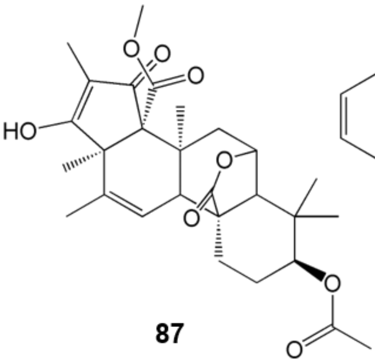

**87**

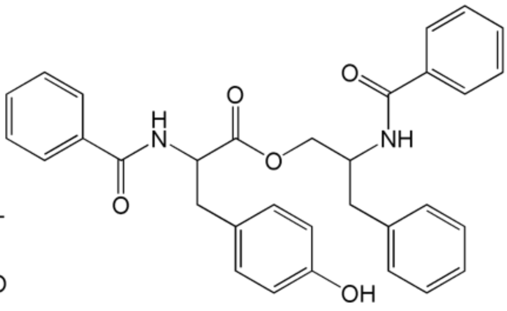

**89**

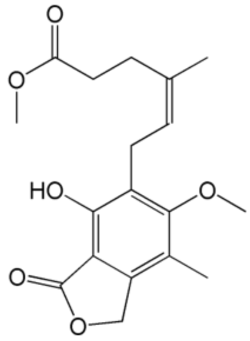

**90**

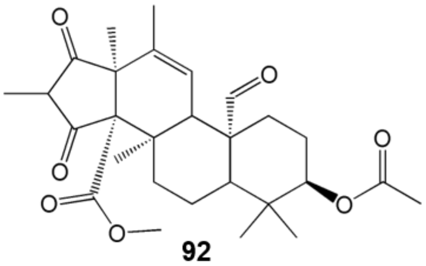

**92**

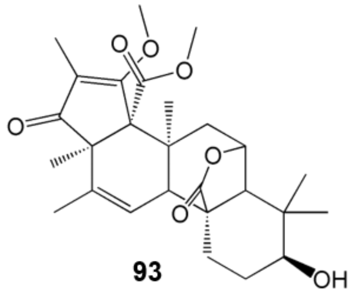

**93**

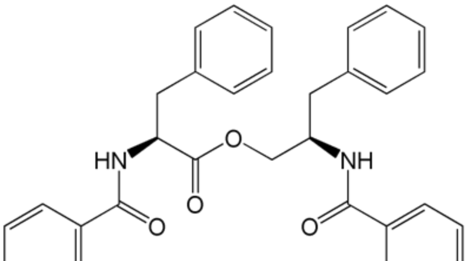

**96**

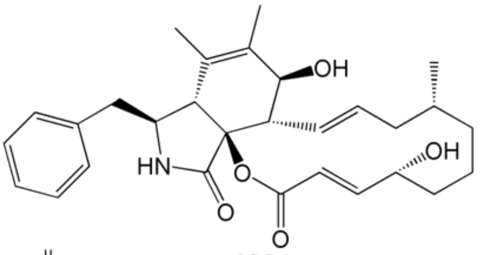

**100**

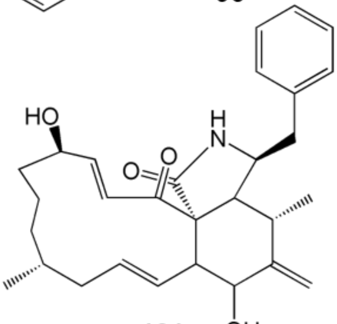

**101**

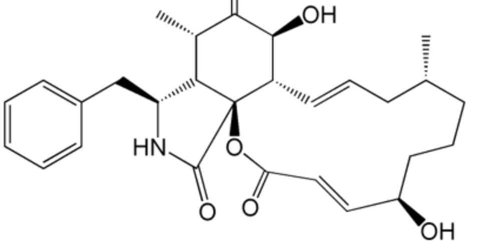

**102**

**Figure S4. (continued)**

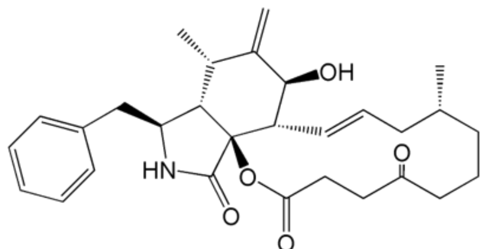

**103**

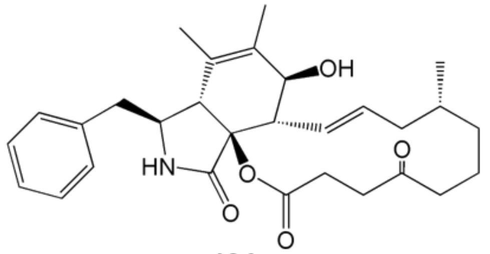

**104**

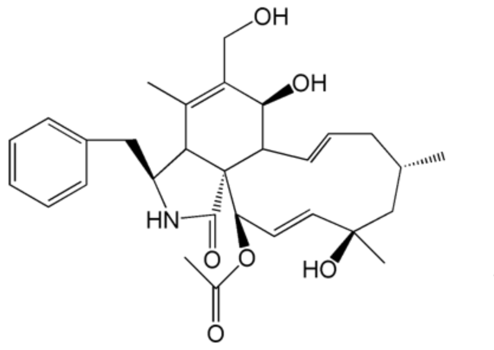

**105**

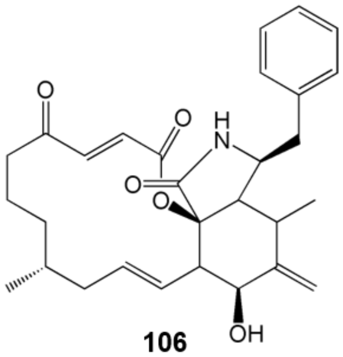

**106**

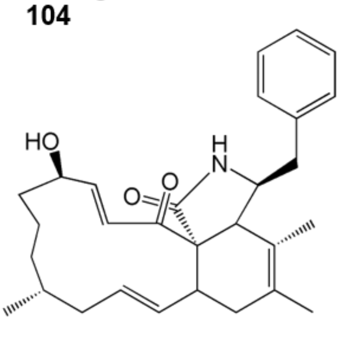

**107**

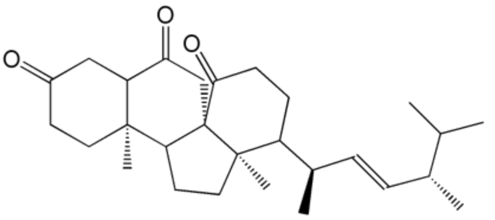

**109**

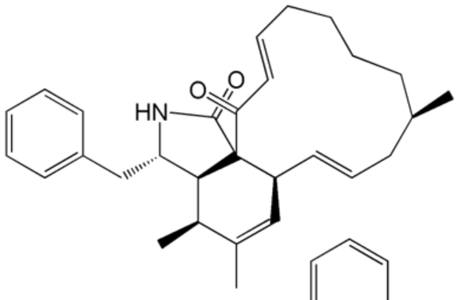

**110**

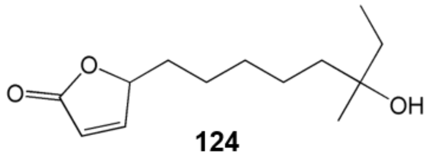

**124**

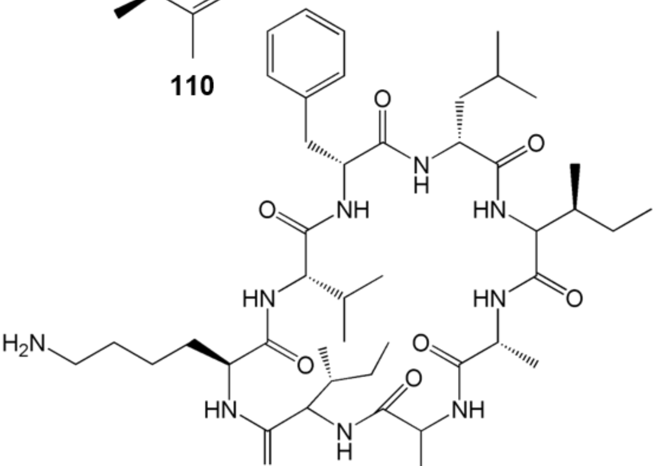

**127**

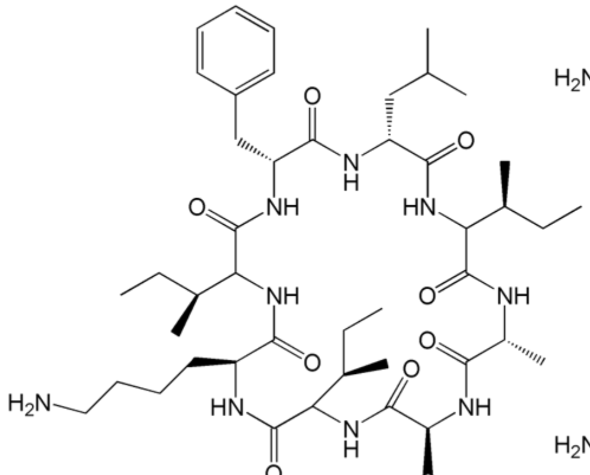

**128**

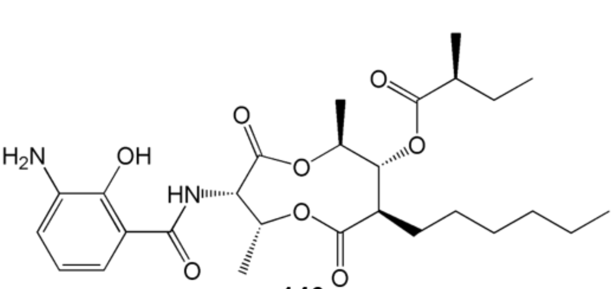

**146**

**Figure S4. (continued)**

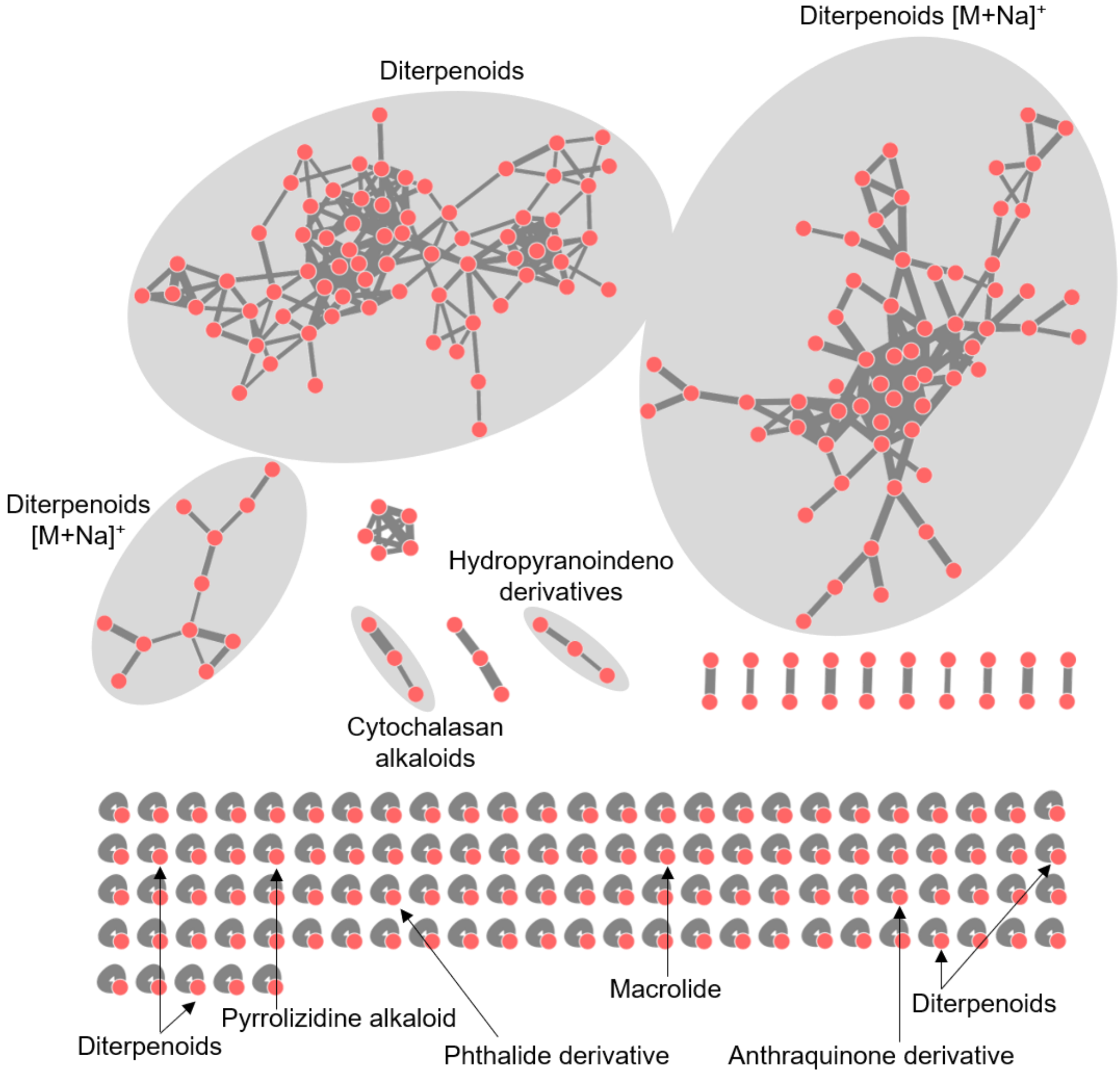


**Figure S5. FBMN of the crude extract of *Pyrenochaeta* sp. strain CHT58 cultivated on PDA medium.** Putatively annotated clusters are highlighted in grey (see Table S6 for putatively annotated compounds).

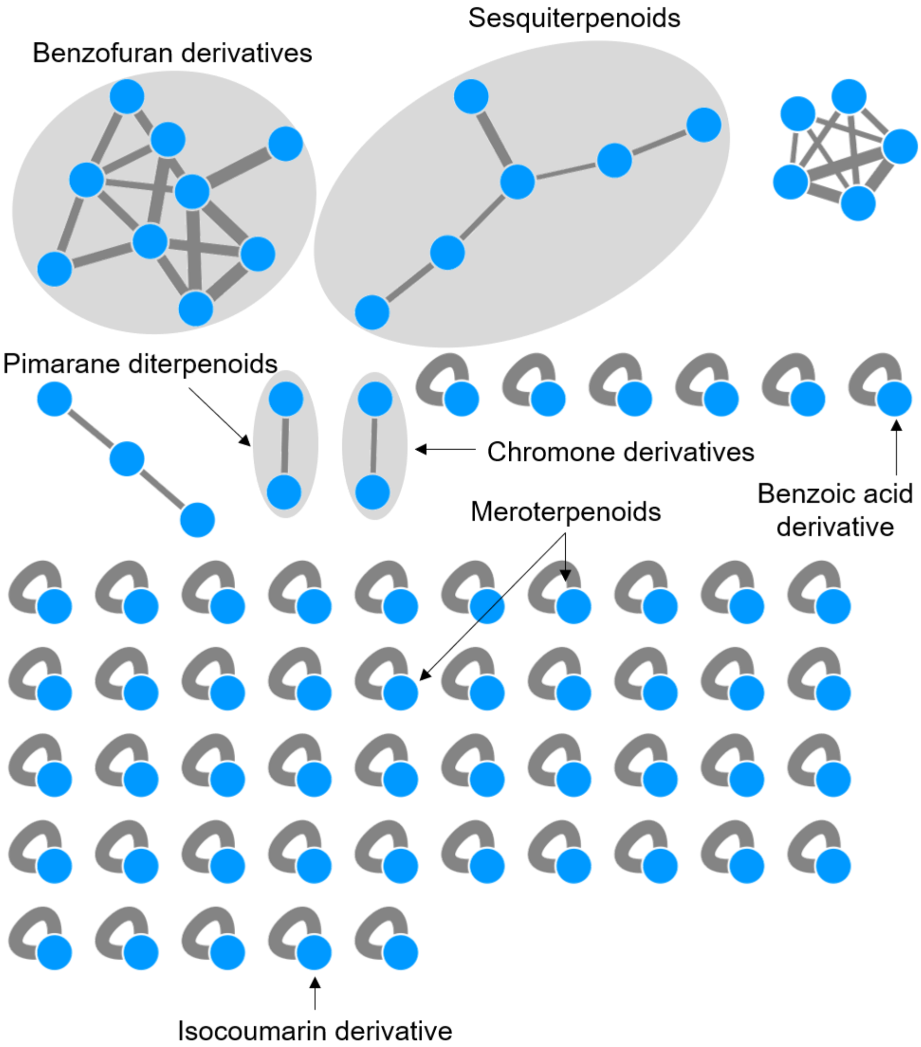


**Figure S6. FBMN of the crude extract of *Pseudogymnoascus destructans* strain CHT56 cultivated on CAG medium.** Putatively annotated clusters are highlighted in grey (see Table S7 for putatively annotated compounds).

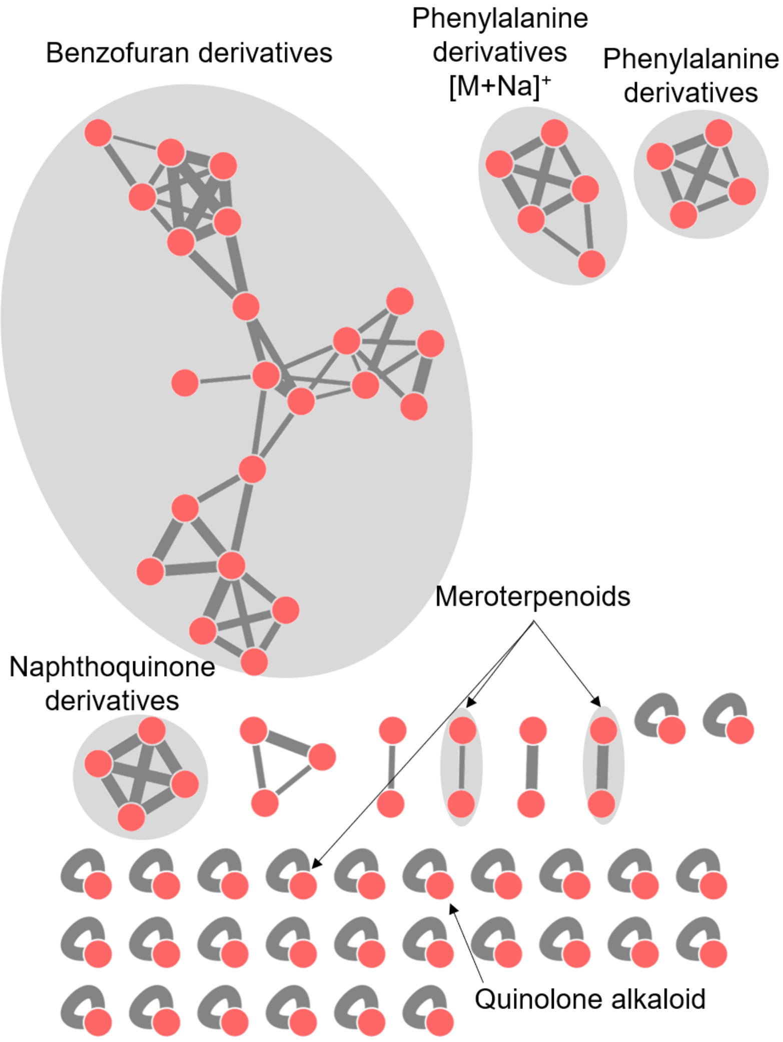


**Figure S7. FBMN of the crude extract of *Penicillium* sp. strain CKT35 cultivated on PDA medium.** Putatively annotated clusters are highlighted in grey (see Table S8 for putatively annotated compounds).

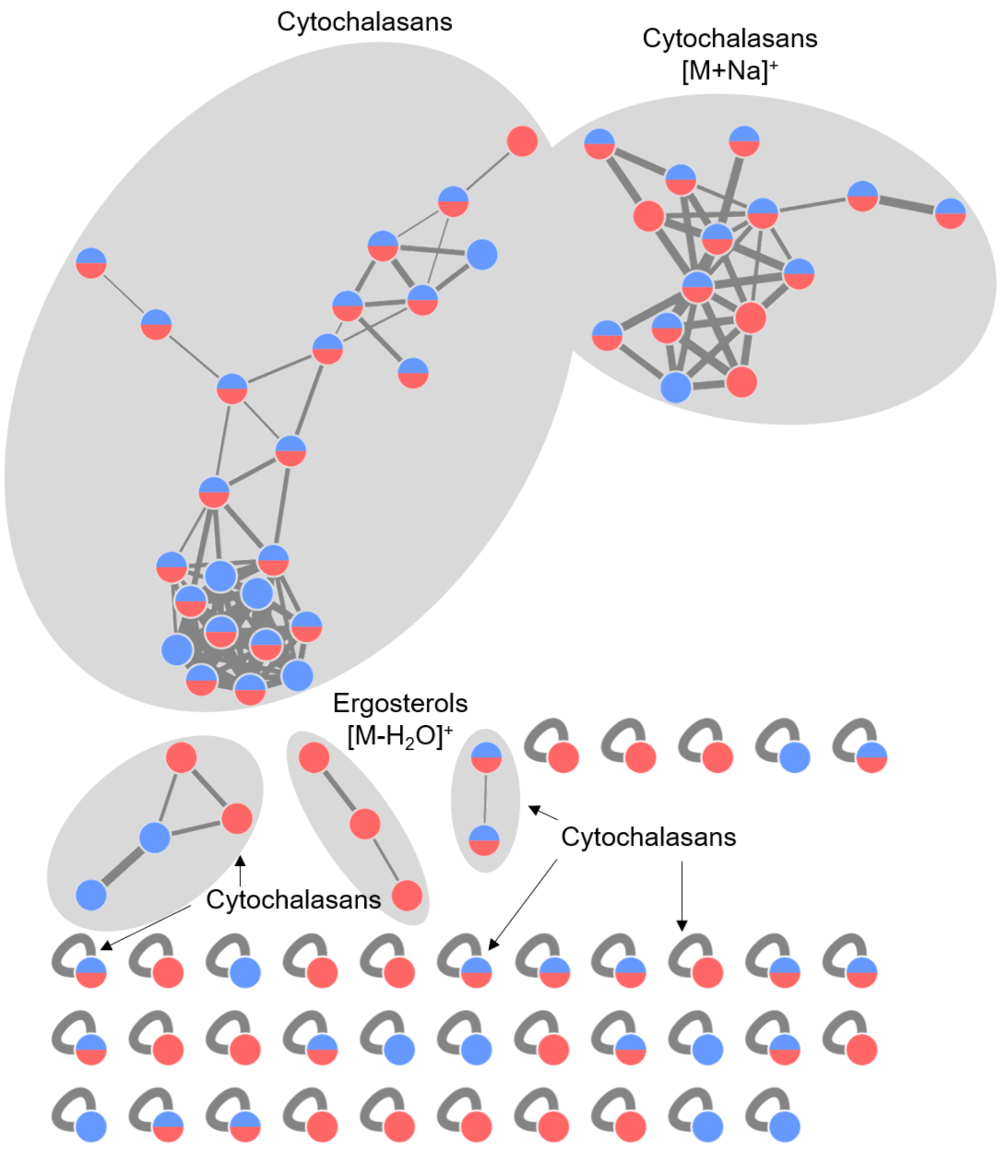


**Figure S8. FBMN of the crude extracts of *Boeremia exigua* strain CKT91 cultivated on CAG (blue nodes) and PDA (red nodes) media.** Putatively annotated clusters are highlighted in grey (see Table S9 for putatively annotated compounds).

**Table S1. Parameters for MZmine-processing of UPLC-MS/MS data.** The applied parameters are given for the global MN (four selected fungal strains) and for the selected seven crude extracts separately for each processing step. $R_t$ = retention time in minutes.

| **Processing step** | **Parameter** | **Four selected fungal strains** | **CHT56-CAG** (*Pseudogymnoascus destructans*) | **CHT58-PDA** (*Pyrenochaeta* sp.) | **CKT35-PDA** (*Penicillium* sp.) | **CKT43-GYM, CKT43-MB** (*Streptomyces* sp.) | **CKT91-CAG, CKT91-PDA** (*Boeremia exigua*) |
|---|---|---|---|---|---|---|---|
| Mass list | MS1 noise level | 1.00E+04 | 1.00E+04 | 3.00E+04 | 3.00E+04 | 3.00E+04 | 3.00E+04 |
| | MS2 noise level | 5.00E+01 | | | | | |
| | $R_t$ | 2-12 | 3-11 | 3-10 | 2-12 | 1-11 | 2-12 |
| Chromatogram building | Min. peak height | 3.00E+04 | 3.00E+04 | 6.00E+04 | 6.00E+04 | 6.00E+04 | 6.00E+04 |
| | *m/z* tolerance | 0.05 Da or 15 ppm | | | | | |
| Deconvolution | Min. peak height | 3.00E+04 | 3.00E+04 | 6.00E+04 | 6.00E+04 | 6.00E+04 | 6.00E+04 |
| | Peak duration | 0.0-0.5 min | | | | | |
| | Baseline level | 1.00E+04 | 1.00E+04 | 3.00E+04 | 3.00E+04 | 3.00E+04 | 3.00E+04 |
| Isotope grouping | *m/z* tolerance | 0.01 Da or 10 ppm | | | | | |
| | $R_t$ tolerance | 0.5 min | | | | | |
| | Maximum charge | 3 | | | | | |
| Alignment | Algorithm | Join aligner | | | | | |
| | *m/z* tolerance | 0.01 Da or 10 ppm | | | | | |
| | $R_t$ tolerance | 0.5 min | | | | | |
| | Weight *m/z*:$R_t$ | 75/25 | | | | | |
| | Detected peaks | 817 | 78 | 284 | 74 | 187 | 86 |

**Table S2. Identification of microbial strains isolated from *C. intestinalis* and seawater reference in Helgoland and Kiel Fjord.** Strains were named after their respective sample type and sampling location (CHT = *C. intestinalis* from Helgoland, tunic; CKT = *C. intestinalis* from Kiel, tunic; HW = Helgoland, seawater; KW = Kiel, seawater) and are given with their isolation medium as well as Genbank accession number (acc. no.). Closest three related strains are given according to BLAST [1] and the resulting lowest possible taxonomic classification. RG = risk group (according to TRBA 460 and TRBA 466), uncult. = uncultured, * = identification to genus by Ribosomal Database Project (RDP; [2]).

| Strain | Medium | Acc. no. | Amplicon | Closest related species (Blast) | Acc. no. closest related species | Lowest taxonomic classification (order) | RG |
|---|---|---|---|---|---|---|---|
| CHT2 | CMN | MW012283 | 16S | *Vibrio* sp.<br>*Vibrio* sp.<br>*Vibrio splendidus* | MG309537.1<br>MG309367.1<br>LS483022.1 | *Vibrio* sp. (Vibrionales) | 2 |
| CHT3 | MA | MW012284 | 16S | *Leisingera aquimarina*<br>*Leisingera aquimarina*<br>Marine alpha proteobacterium BBAT3 | KX218295.1<br>KX218294.1<br>AF365994.1 | *Leisingera aquimarina* (Rhodobacterales) | 1 |
| CHT5 | MA | MW012285 | 16S | *Shewanella pneumatophori*<br>Uncult. bacterium 6-36A<br>Uncult. bacterium 5-20A | MH169286.1<br>MG952522.1<br>MG952508.1 | *Shewanella* sp. (Alteromonadales) | 1 |
| CHT6 | MA | MW012286 | 16S | *Vibrio* sp.<br>*Vibrio* sp.<br>*Vibrio gigantis* | KF188534.1<br>KF188493.1<br>GU194170.1 | *Vibrio gigantis* (Vibrionales) | 1 |
| CHT7 | MA | MW012287 | 16S | *Ruegeria* sp.<br>Uncult. bacterium Woods-Hole_a4093<br>*Ruegeria faecimaris* | KY513434.1<br>KF799356.1<br>NR_104546.1 | *Ruegeria faecimaris* (Rhodobacterales) | 1 |
| CHT8 | MA | MW012288 | 16S | *Ruegeria* sp.<br>*Ruegeria* sp.<br>*Ruegeria atlantica* | KY363633.1<br>KX833139.1<br>JN128252.1 | *Ruegeria atlantica* (Rhodobacterales) | 1 |
| CHT9 | CMN | MW012289 | 16S | *Vibrio hemicentroti*<br>*Vibrio* sp.<br>*Vibrio* sp. | LS482994.1<br>LC416556.1<br>LC416555.1 | *Vibrio* sp. (Vibrionales) | 1 |
| CHT10 | CMN | MW012290 | 16S | *Kangiella* sp.<br>*Kangiella* sp.<br>*Kangiella sediminilitoris* | MG889588.2<br>KP795388.1<br>CP012418.1 | *Kangiella* sp. (Oceanospirillales) | 1 |
| CHT13 | WSP30 | MW012291 | 16S | *Aurantimonas coralicida*<br>*Aurantimonas litoralis*<br>*Aurantimonas manganoxydans* | MH725320.1<br>KR140222.1<br>LC066380.1 | *Aurantimonas* sp. (Rhizobiales) | 1 |

| Strain | Medium | Acc. no. | Amplicon | Closest related species (Blast) | Acc. no. closest related species | Lowest taxonomic classification (order) | RG |
|---|---|---|---|---|---|---|---|
| CHT14 | WSP30 | MW012292 | 16S | *Photobacterium damselae*<br>*Photobacterium damselae*<br>*Photobacterium damselae* | MG386399.1<br>MH368432.1<br>CP018297.1 | *Photobacterium damselae* (Vibrionales) | 2 |
| CHT15 | TSB3+10 | MW012293 | 16S | *Bacillus* sp.<br>*Bacillus* sp.<br>*Bacillus altitudinis* | MG970354.1<br>MG970353.1<br>MG970351.1 | *Bacillus* sp. (Bacillales) | 1 |
| CHT16 | MA | MW012294 | 16S | Uncult. *Vibrio* sp.<br>Uncult. *Vibrio* sp.<br>*Vibrio anguillarum* | MG554532.1<br>MG554529.1<br>CP022468.1 | *Vibrio* sp. (Vibrionales) | 2 |
| CHT17 | MA | MW012295 | 16S | Uncult. bacterium JS10_F09<br>*Vibrio chagasii*<br>*Vibrio chagasii* | KT318724.1<br>LN832958.1<br>LN832949.1 | *Vibrio* sp. (Vibrionales) | 1 |
| CHT18 | TSB3+10 | MW012296 | 16S | *Brevundimonas vesiculari*<br>*Brevundimonas vesiculari*<br>*Brevundimonas nasdae* | MG819328.1<br>MG685726.1<br>MG322225.1 | *Brevundimonas* sp. (Caulobacterales) | 2 |
| CHT22a | CMN | MW012297 | 16S | *Marixanthomonas ophiurae*<br>Uncult. bacterium denovo39636<br>Uncult. bacterium denovo37181 | MK215855.1<br>KU635267.1<br>KU633651.1 | *Marixanthomonas ophiurae* (Flavobacteriales) | 1 |
| CHT22b | MA | MW012298 | 16S | Marine bacterium I4017<br>Uncult. *Vibrio* sp. MUM_Aug34<br>*Vibrio pectenicida* | KJ469389.1<br>KC108888.1<br>NR_118241.1 | *Vibrio* sp. (Vibrionales) | 1 |
| CHT23 | MA | MW012299 | 16S | *Amphritea* sp.<br>Bacterium GAA07<br>*Amphritea spongicola* | KP843673.1<br>KP684316.1<br>NR_135881.1 | *Amphritea spongicola* (Oceanospirillales) | 1 |
| CHT25 | TSB3+10 | MW012300 | 16S | *Pseudorhodobacter aquimaris*<br>*Rhodobacter* sp.<br>*Rhodobacter* sp. | NR_108680.1<br>EU979476.1<br>EU979477.1 | *Pseudorhodobacter aquimaris* (Rhodobacterales) | 1 |
| CHT27 | WSP30 | MW012301 | 16S | *Bacillus velezensis*<br>*Bacillus velezensis*<br>*Bacillus velezensis* | MG970354.1<br>MG970353.1<br>MG970351.1 | *Bacillus* sp. (Bacillales) | 1 |
| CHT28 | WSP30 | MW012302 | 16S | *Leisingera aquimarina*<br>*Leisingera aquimarina*<br>Marine alpha proteobacterium BBAT3 | KX218295.1<br>KX218294.1<br>AF365994.1 | *Leisingera aquimarina* (Rhodobacterales) | 1 |

| Strain | Medium | Acc. no. | Amplicon | Closest related species (Blast) | Acc. no. closest related species | Lowest taxonomic classification (order) | RG |
|---|---|---|---|---|---|---|---|
| CHT29 | TSB3+10 | MW012303 | 16S | *Arenibacter* sp.<br>*Arenibacter* sp.<br>*Arenibacter troitsensis* | KY810503.1<br>HG529986.1<br>JQ898112.1 | *Arenibacter* sp. (Flavobacteriales) | 1 |
| CHT30 | WSP30 | MW012304 | 16S | *Micrococcus yunnanensis*<br>*Micrococcus aloeverae*<br>*Micrococcus aloeverae* | MG649988.1<br>MG028596.1<br>MG561895.1 | *Micrococcus* sp. (Micrococcales) | 1 |
| CHT32 | PDA | MW017476 | ITS | *Fusarium venenatum*<br>*Fusarium venenatum*<br>*Fusarium venenatum* | MH681155.1<br>MH681152.1<br>NR_156290.1 | *Fusarium* sp. (Hypocreales) | 2 |
| CHT33 | PDA | MW017477 | ITS | *Fusarium venenatum*<br>*Fusarium venenatum*<br>*Fusarium venenatum* | MH681155.1<br>MH681152.1<br>NR_156290.1 | *Fusarium* sp. (Hypocreales) | 2 |
| CHT34 | WSP30 | MW012305 | 16S | *Vibrio* sp.<br>*Vibrio* sp.<br>*Vibrio parahaemolyticus* | MK167378.1<br>MH997741.1<br>MK053885.1 | *Vibrio* sp. (Vibrionales) | 2 |
| CHT35 | WSP30 | MW017478 | ITS | *Cladosporium* sp.<br>*Cladosporium cf. cladosporioides*<br>*Cladosporium cf. cladosporioides* | MF510502.1<br>MH399546.1<br>MH399542.1 | *Cladosporium* sp. (Capnodiales) | 1 |
| CHT37 | WSP30 | MW017479,<br>MW012374,<br>MW017496 | ITS,<br>18S,<br>28S | *Emericellopsis maritima*<br>*Acremonium breve*<br>*Acremonium persicinum,*<br>*Emericellopsis pallida*<br>*Emericellopsis pallida*<br>*Emericellopsis maritima,*<br>*Acremonium* sp.<br>*Emericellopsis alkalina*<br>*Emericellopsis alkalina*<br>*Emericellopsis alkalina* | MH871998.1<br>MH859569.1<br>MG813195.1,<br>MH443384.1<br>NG_062927.1<br>NG_062926.1,<br>KC987248.1<br>KC987247.1<br>KC987234.1 | *Emericellopsis maritima* (Hypocreales) | 1 |
| CHT39 | MA | MW012306 | 16S | *Vibrio* sp.<br>*Vibrio anguillarum*<br>*Vibrio anguillarum* | MG788349.1<br>CP023433.1<br>CP023293.1 | *Vibrio* sp. (Vibrionales) | 2 |
| CHT40 | WSP30 | MW012375 | 18S | *Pseudochaetosphaeronema larense*<br>*Pseudochaetosphaeronema martinelli*<br>*Didymosphaeria variabile* | NG_061147.1<br>NG_062412.1<br>NG_064914.1 | *Pseudochaetosphaeronema larense* (Pleosporales) | 1 |

| Strain | Medium | Acc. no. | Amplicon | Closest related species (Blast) | Acc. no. closest related species | Lowest taxonomic classification (order) | RG |
|---|---|---|---|---|---|---|---|
| CHT41 | WSP30 | MW012307 | 16S | *Mycolicibacterium monacense*<br>*Mycolicibacterium doricum*<br>*Mycolicibacterium aichiense* | AP022617.1<br>AP022605.1<br>AP022561.1 | *Mycolicibacterium* sp. (Corynebacteriales) | 1 |
| CHT42 | WSP30 | MW012308 | 16S | *Ruegeria lacuscaerulensis*<br>*Ruegeria* sp.<br>*Ruegeria* sp. | MH283809.1<br>MG819700.1<br>MG996714.1 | *Ruegeria* sp. (Rhodobacterales) | 1 |
| CHT43 | WSP30 | MW012309 | 16S | *Primorskyibacter* sp.<br>*Thalassococcus* sp.<br>*Thalassococcus lentus* | KY086433.2<br>MG889583.2<br>NR_109663.1 | *Primorskyibacter* sp. (Rhodobacterales) | 1 |
| CHT46 | MA | MW012310 | 16S | *Litoreibacter albidus*<br>*Litoreibacter* sp.<br>*Litoreibacter ascidiaceicola* | KX961718.1<br>KJ786461.1<br>NR_134068.1 | *Litoreibacter* sp. (Rhodobacterales) | 1 |
| CHT47 | MA | MW012311 | 16S | *Roseovarius arcticus*<br>*Roseovarius arcticus*<br>*Sulfitobacter* sp. | MK617616.1<br>NR_169499.1<br>FJ889642.1 | *Roseovarius arcticus* (Rhodobacterales) | 1 |
| CHT48 | MA | MW012312 | 16S | *Vibrio rumoiensis*<br>*Vibrio* sp.<br>*Vibrio* sp. | AP018685.1<br>MF537054.1<br>MF537053.1 | *Vibrio* sp. (Vibrionales) | 1 |
| CHT49 | CMN | MW012313 | 16S | *Ruegeria atlantica*<br>*Ruegeria* sp.<br>Bacterium CSR-55 | HE584803.1<br>LC053425.1<br>KJ018058.1 | *Ruegeria atlantica* (Rhodobacterales) | 1 |
| CHT50 | TSB3+10 | MW017480,<br>MW012376,<br>MW017497 | ITS,<br>18S,<br>28S | Uncult. fungus C2_EH11<br>Melanized limestone ascomycete CR-2004<br>*Cladophialophora chaetospira,*<br>*Cladophialophora boppii*<br>*Cladophialophora boppii*<br>*Fonsecaea nubica,*<br>*Phialophora verrucosa*<br>*Phialophora verrucosa*<br>*Phialophora verrucosa* | JX042985.1<br>AY559331.1<br>EU035403.1,<br>NG_062637.1<br>AJ232946.1<br>GU197483.1,<br>AB550778.1<br>AB550777.1<br>AB550776.1 | Herpotrichiellaceae unclassified (Chaetothyriales) | 2 |
| CHT51 | CMN | MW012314 | 16S | *Vibrio alginolyticus*<br>*Vibrio* sp.<br>*Vibrio* sp. | CP017916.1<br>KX453258.1<br>KX453256.1 | *Vibrio* sp. (Vibrionales) | 2 |

| Strain | Medium | Acc. no. | Amplicon | Closest related species (Blast) | Acc. no. closest related species | Lowest taxonomic classification (order) | RG |
|---|---|---|---|---|---|---|---|
| CHT52 | TSB3+10 | MW012315 | 16S | *Arenibacter* sp.<br>*Arenibacter* sp.<br>*Arenibacter latericius* | KY810503.1<br>HG529986.1<br>NR_024893.1 | *Arenibacter* sp. (Flavobacteriales) | 2 |
| CHT53 | WSP30 | MW012316 | 16S | *Ochrobactrum* sp.<br>*Ochrobactrum* sp.<br>*Ochrobactrum pseudogrignonense* | KX822681.1<br>KJ777141.1<br>GU991856.1 | *Ochrobactrum pseudogrignonense* (Rhizobiales) | 1 |
| CHT54 | WSP30 | MW012317 | 16S | *Bacillus amyloliquefaciens*<br>*Bacillus amyloliquefaciens*<br>*Bacillus* sp. | MH910761.1<br>MH910713.1<br>MG309364.1 | *Bacillus* sp. (Bacillales) | 1 |
| CHT55 | WSP30 | MW012318 | 16S | *Ochrobactrum* sp.<br>*Ochrobactrum* sp.<br>*Ochrobactrum grignonense* | KX822681.1<br>KJ777141.1<br>FJ950543.1 | *Ochrobactrum grignonense* (Rhizobiales) | 1 |
| CHT56 | PDA | MW012377 | 18S | *Pseudogymnoascus destructans*<br>*Geomyces destructans*<br>*Geomyces destructans* | KF866376.1<br>GU350433.1<br>GQ489025.1 | *Pseudogymnoascus destructans* (Leotiomycetes *incertae sedis*) | 1 |
| CHT58 | PDA | MW017481 | ITS | *Pyrenochaeta unguis-hominis*<br>*Pyrenochaeta unguis-hominis*<br>*Pyrenochaeta unguis-hominis* | KP794081.1<br>KP132548.1<br>KP132547.1 | *Pyrenochaeta* sp. (Pleosporales) | 1 |
| CKT1 | MA | MW012319 | 16S | *Pseudomonas* sp.<br>*Pseudomonas* sp.<br>*Pseudomonas anguilliseptica* | NR_042607.1<br>NR_042451.1<br>NR_044569.1 | *Pseudomonas* sp. (Pseudomonadales) | 1 |
| CKT2* | MA | MW012320 | 16S | *Litoreibacter janthinus*<br>*Thalassobacter* sp.<br>*Roseovarius* sp. | NR_112983.1<br>FR821226.1<br>FJ425225.1 | *Litoreibacter* sp. (Rhodobacterales) | 1 |
| CKT3* | MA | MW012321 | 16S | *Pelagicola litoralis*<br>*Roseobacter* sp.<br>Uncult. bacterium SF-Oct-32 | NR_044158.1<br>EU195951.1<br>HQ225294.1 | *Pelagicola* sp. (Rhodobacterales) | 1 |
| CKT4 | MA | MW012322 | 16S | *Neptunomonas concharum*<br>Uncult. bacterium Q31008<br>Uncult. bacterium Stn3_Sep_26 | NR_118152.1<br>JX193435.1<br>KX014554.1 | *Neptunomonas concharum* (Oceanospirillales) | 1 |
| CKT5 | MA | MW012323 | 16S | *Pseudomonas peli*<br>*Pseudomonas* sp.<br>*Pseudomonas* sp. | MF077147.1<br>MG786374.1<br>MG758017.1 | *Pseudomonas* sp. (Pseudomonadales) | 1 |

| Strain | Medium | Acc. no. | Amplicon | Closest related species (Blast) | Acc. no. closest related species | Lowest taxonomic classification (order) | RG |
|---|---|---|---|---|---|---|---|
| CKT6 | MA | MW012324 | 16S | *Flaviramulus* sp.<br>*Flaviramulus* sp.<br>*Flaviramulus ichthyoenteri* | KC756867.1<br>JX431889.1<br>NR_118464.1 | *Flaviramulus ichthyoenteri* (Flavobacteriales) | 1 |
| CKT7 | MA | MW012325 | 16S | *Vibrio aestuarianus*<br>*Vibrio aestuarianus*<br>*Vibrio aestuarianus* | AJ845015.1<br>AJ845014.1<br>AJ845012.1 | *Vibrio aestuarianus* (Vibrionales) | 1 |
| CKT8 | CMB | MW012326 | 16S | *Pseudomonas peli*<br>*Pseudomonas* sp.<br>*Pseudomonas* sp. | MF077147.1<br>MG786374.1<br>MG758017.1 | *Pseudomonas* sp. (Pseudomonadales) | 1 |
| CKT10 | CMB | MW012327 | 16S | *Pseudomonas guineae*<br>*Pseudomonas peli*<br>*Pseudomonas cuatrocienegasensis* | MH392634.1<br>KJ643969.1<br>MF077147.1 | *Pseudomonas* sp. (Pseudomonadales) | 1 |
| CKT11 | CMB | MW012328 | 16S | *Vibrio aestuarianus*<br>*Vibrio* sp.<br>*Vibrio* sp. | NR_113780.1<br>HQ449463.1<br>HM012774.1 | *Vibrio aestuarianus* (Vibrionales) | 1 |
| CKT12 | CMB | MW012329 | 16S | *Pseudomonas peli*<br>*Pseudomonas* sp.<br>*Pseudomonas* sp. | MF077147.1<br>MG786374.1<br>MG758017.1 | *Pseudomonas* sp. (Pseudomonadales) | 1 |
| CKT14 | CMN | MW012330 | 16S | *Pseudomonas* sp.<br>Uncult. bacterium HJ-38<br>*Pseudomonas peli* | MH392634.1<br>KJ643969.1<br>MF077147.1 | *Pseudomonas* sp. (Pseudomonadales) | 1 |
| CKT15 | TSB3+10 | MW012331 | 16S | *Pseudomonas* sp.<br>Uncult. bacterium HJ-38<br>*Pseudomonas peli* | MH392634.1<br>KJ643969.1<br>MF077147.1 | *Pseudomonas* sp. (Pseudomonadales) | 1 |
| CKT16 | TSB3+10 | MW012332 | 16S | *Hydrogenophaga* sp.<br>*Hydrogenophaga crassostreae*<br>Uncult. *Hydrogenophaga* sp. TST2N32 | KU198320.2<br>CP017476.1<br>KX119551.1 | *Hydrogenophaga crassostreae* (Burkholderiales) | 1 |
| CKT17 | TSB3+10 | MW012333 | 16S | Bacterium BW3PhG33<br>*Lysobacter* sp.<br>*Lysobacter spongiicola* | KC012871.1<br>GU217698.1<br>NR_041587.1 | *Lysobacter spongiicola* (Xanthomonadales) | 1 |
| CKT18 | TSB3+10 | MW012334 | 16S | *Vibrio aestuarianus*<br>*Vibrio* sp.<br>*Vibrio* sp. | NR_113780.1<br>HQ449463.1<br>HM012774.1 | *Vibrio aestuarianus* (Vibrionales) | 1 |

| Strain | Medium | Acc. no. | Amplicon | Closest related species (Blast) | Acc. no. closest related species | Lowest taxonomic classification (order) | RG |
|---|---|---|---|---|---|---|---|
| CKT19 | TSB3+10 | MW012335 | 16S | *Vibrio anguillarum*<br>*Vibrio anguillarum*<br>*Vibrio anguillarum* | MG264177.1<br>CP023310.1<br>CP023054.1 | Vibrio *anguillarum* (Vibrionales) | 2 |
| CKT20 | WSP30 | MW012336 | 16S | *Bacillus pumilus*<br>*Bacillus pumilus*<br>*Bacillus pumilus* | MF077157.1<br>MH045994.1<br>MH045860.1 | *Bacillus* sp. (Bacillales) | 1 |
| CKT21 | CMN | MW012337 | 16S | *Pseudomonas guineae*<br>*Pseudomonas peli*<br>*Pseudomonas glareae* | NR_042607.1<br>NR_042451.1<br>NR_145562.1 | *Pseudomonas* sp. (Pseudomonadales) | 1 |
| CKT22 | CMN | MW012338 | 16S | Uncult. *Roseobacter* sp. C139300178<br>Uncult. *Roseobacter* sp. C139300006<br>*Phaeobacter arcticus* | JX528567.1<br>JX528395.1<br>NR_043888.1 | *Phaeobacter arcticus* (Rhodobacterales) | 1 |
| CKT23 | CMN | MW012339 | 16S | *Shewanella* sp.<br>*Shewanella* sp.<br>*Shewanella colwelliana* | MF045124.1<br>MF045122.1<br>KX756553.1 | *Shewanella* sp. (Alteromonadales) | 1 |
| CKT24 | CMN | MW012340 | 16S | *Arenibacter* sp.<br>*Arenibacter* sp.<br>*Arenibacter echinorum* | KU948154.1<br>KF273918.1<br>KF911336.1 | *Arenibacter echinorum* (Flavobacteriales) | 1 |
| CKT25 | TSB3+10 | MW012341 | 16S | *Vibrio aestuarianus*<br>*Vibrio* sp.<br>*Vibrio* sp. | NR_113780.1<br>HQ449463.1<br>HM012774.1 | *Vibrio aestuarianus* (Vibrionales) | 1 |
| CKT28 | WSP30 | MW017482 | ITS | *Cyphellophora reptans*<br>*Cyphellophora reptans*<br>*Phialophora reptans* | NR_121346.1<br>EU514699.1<br>AB190380.1 | *Cyphellophora reptans* (Chaetothyriales) | 1 |
| CKT29 | MA | MW012342 | 16S | *Marinobacter* sp.<br>Uncult. bacterium AB-4<br>*Marinobacter litoralis* | KY770365.1<br>KX651417.1<br>KY926903.1 | *Marinobacter litoralis* (Alteromonadales) | 1 |
| CKT30* | MA | MW012343 | 16S | Uncult. Bacteroidetes bacterium D<br>*Salegentibacter* sp.<br>*Salinimicrobium marinum* | KC169760.1<br>AY576719.1<br>GQ866113.1 | *Salinimicrobium* sp. (Flavobacteriales) | 1 |
| CKT32 | TSB3+10 | MW012344 | 16S | *Pseudomonas* sp.<br>*Pseudomonas* sp.<br>*Pseudomonas peli* | MH815093.1<br>MH814721.1<br>MG581693.1 | *Pseudomonas* sp. (Pseudomonadales) | 1 |

| Strain | Medium | Acc. no. | Amplicon | Closest related species (Blast) | Acc. no. closest related species | Lowest taxonomic classification (order) | RG |
|---|---|---|---|---|---|---|---|
| CKT33 | CMN | MW012345 | 16S | *Vibrio splendidus*<br>*Vibrio* sp.<br>*Vibrio anguillarum* | MH010050.1<br>MG788349.1<br>CP023433.1 | *Vibrio* sp. (Vibrionales) | 2 |
| CKT34 | CMN | MW012346 | 16S | *Arthrobacter* sp.<br>*Arthrobacter* sp.<br>*Arthrobacter citreus* | MH018914.1<br>JN006271.1<br>GQ149484.1 | *Arthrobacter* sp. (Micrococcales) | 1 |
| CKT35 | WSP30 | MW017483 | ITS | *Penicillium bialowiezense*<br>*Penicillium brevicompactum*<br>*Penicillium biourgeianum* | MH854996.1<br>MH481701.1<br>KX067821.1 | *Penicillium* sp. (Eurotiales) | 1 |
| CKT36 | WSP30 | MW012347 | 16S | *Bacillus pumilus*<br>*Bacillus pumilus*<br>*Bacillus pumilus* | MF077157.1<br>MH045994.1<br>MH045860.1 | *Bacillus* sp. (Bacillales) | 1 |
| CKT37 | TSB3+10 | MW012348 | 16S | *Vibrio aestuarianus*<br>*Vibrio aestuarianus*<br>*Vibrio aestuarianus* | AJ845015.1<br>AJ845014.1<br>AJ845012.1 | *Vibrio aestuarianus* (Vibrionales) | 1 |
| CKT38 | TSB3+10 | MW012349 | 16S | *Bacillus mycoides*<br>*Bacillus* sp.<br>*Bacillus* sp. | MH169305.1<br>MF948894.1<br>MH096031.1 | *Bacillus* sp. (Bacillales) | 1 |
| CKT39 | CMN | MW012350 | 16S | *Streptomyces* sp.<br>*Streptomyces* sp.<br>*Streptomyces badius* | MK292047.1<br>MK271721.1<br>MK156399.1 | *Streptomyces* sp. (Streptomycetales) | 1 |
| CKT41 | WSP30 | MW012351 | 16S | *Bacillus amyloliquefaciens*<br>*Bacillus halotolerans*<br>*Bacillus amyloliquefaciens* | MH236415.1<br>MH236414.1<br>MH236413.1 | *Bacillus* sp. (Bacillales) | 1 |
| CKT43 | WSP30 | MW012352 | 16S | *Streptomyces sampsonii*<br>*Streptomyces* sp.<br>*Streptomyces* sp. | MK878388.1<br>MK129408.1<br>MK129407.1 | *Streptomyces* sp. (Streptomycetales) | 1 |
| CKT48 | WSP30 | MW012353 | 16S | *Bacillus pumilus*<br>*Bacillus pumilus*<br>*Bacillus pumilus* | MF077157.1<br>MH045994.1<br>MH045860.1 | *Bacillus* sp. (Bacillales) | 1 |
| CKT49 | WSP30 | MW017484 | ITS | *Penicillium brasilianum*<br>*Penicillium brasilianum*<br>*Penicillium brasilianum* | KY469061.1<br>KY469042.1<br>LT558939.1 | *Penicillium brasilianum* (Eurotiales) | 1 |
| CKT50 | WSP30 | MW012354 | 16S | Uncult. bacterium f6h4<br>Uncult. bacterium f4s2<br>*Yokenella regensburgei* | DQ068814.1<br>DQ068792.1<br>KJ397957.1 | Enterobacteriaceae unclassified (Enterobacterales) | 2 |

| Strain | Medium | Acc. no. | Amplicon | Closest related species (Blast) | Acc. no. closest related species | Lowest taxonomic classification (order) | RG |
|---|---|---|---|---|---|---|---|
| CKT51-I | WSP30 | MW012355 | 16S | *Shewanella putrefaciens*<br>*Shewanella hafniensis*<br>*Shewanella putrefaciens* | CP028435.1<br>MF612155.1<br>KX271690.1 | *Shewanella* sp. (Alteromonadales) | 2 |
| CKT51-II | WSP30 | MW012356 | 16S | *Bacillus* sp.<br>*Bacillus* sp.<br>*Bacillus megaterium* | MF418041.1<br>MF418038.1<br>MH179091.1 | *Bacillus* sp. (Bacillales) | 1 |
| CKT52 | MA | MW012357 | 16S | *Bizionia* sp.<br>*Bizionia* sp.<br>*Bizionia fulviae* | KX066849.1<br>HF912806.2<br>NR_137258.1 | *Bizionia fulviae* (Flavobacteriales) | 1 |
| CKT54 | TSB3+10 | MW017485 | ITS | *Fusarium* sp.<br>*Fusarium oxysporum*<br>*Fusarium oxysporum* | KU556574.1<br>HQ603748.1<br>KY949601.1 | *Fusarium* sp. (Hypocreales) | 2 |
| CKT55 | WSP30 | MW017486 | ITS | *Boeremia exigua*<br>*Boeremia exigua*<br>*Boeremia exigua* | KY949620.1<br>KY419536.1<br>MF925487.1 | *Boeremia exigua* (Pleosporales) | 1 |
| CKT56 | MA | MW012358 | 16S | *Ruegeria* sp.<br>Uncult. bacterium APY15<br>*Ruegeria atlantica* | MH023307.1<br>JQ347396.1<br>HE584803.1 | *Ruegeria atlantica* (Rhodobacterales) | 1 |
| CKT57 | MA | MW012359 | 16S | *Pseudomonas* sp.<br>Uncult. bacterium HJ-38<br>*Pseudomonas peli* | MH392634.1<br>KJ643969.1<br>MF077147.1 | *Pseudomonas* sp. (Pseudomonadales) | 1 |
| CKT58 | CMN | MW017487 | ITS | *Penicillium bialowiezense*<br>Fungal sp. PdlM07-12<br>*Penicillium biourgeianum* | MH854996.1<br>MG923832.1<br>KX067821.1 | *Penicillium* sp. (Eurotiales) | 1 |
| CKT59 | CMN | MW012360 | 16S | *Pseudomonas* sp.<br>Uncult. bacterium HJ-38<br>*Pseudomonas peli* | MH392634.1<br>KJ643969.1<br>MF077147.1 | *Pseudomonas* sp. (Pseudomonadales) | 1 |
| CKT60 | CMN | MW012361 | 16S | *Kiloniella laminariae*<br>*Kiloniella* sp.<br>Uncult. alpha proteobacterium MERTZ_0CM_263 | NR_042646.1<br>KM101108.2<br>AF425762.1 | *Kiloniella laminariae* (Kiloniellales) | 1 |
| CKT61 | CMB | MW012362 | 16S | *Pseudomonas guineae*<br>*Pseudomonas peli*<br>*Pseudomonas glareae* | NR_042607.1<br>NR_042451.1<br>NR_145562.1 | *Pseudomonas* sp. (Pseudomonadales) | 1 |

| Strain | Medium | Acc. no. | Amplicon | Closest related species (Blast) | Acc. no. closest related species | Lowest taxonomic classification (order) | RG |
|---|---|---|---|---|---|---|---|
| CKT62 | CMB | MW012363 | 16S | *Vibrio aestuarianus*<br>*Vibrio* sp.<br>*Vibrio* sp. | NR_113780.1<br>HQ449463.1<br>HM012774.1 | *Vibrio aestuarianus* (Vibrionales) | 1 |
| CKT65 | MA | MW012364 | 16S | *Salegentibacter* sp.<br>Uncult. bacterium BF2009_Sep_21m_E5<br>*Salegentibacter salarius* | FR772274.1<br>JX864700.1<br>NR_044244.1 | *Salegentibacter* sp. (Flavobacteriales) | 1 |
| CKT67 | TSB3+10 | MW012365 | 16S | *Serinicoccus* sp.<br>*Serinicoccus* sp.<br>*Serinicoccus chungangensis* | CP014989.1<br>DQ985074.1<br>NR_117788.1 | *Serinicoccus* sp. (Micrococcales) | 1 |
| CKT68 | WSP30 | MW012366 | 16S | *Paracoccus aquimaris*<br>*Paracoccus sp.*<br>*Paracoccus aquimaris* | NR_148324.1<br>LC094992.1<br>KP716798.1 | *Paracoccus* sp. (Rhodobacterales) | 2 |
| CKT74 | CMB | MW012367 | 16S | *Pseudomonas* sp.<br>Uncult. bacterium HJ-38<br>*Pseudomonas peli* | MH392634.1<br>KJ643969.1<br>MF077147.1 | *Pseudomonas* sp. (Pseudomonadales) | 1 |
| CKT75 | CMN | MW012368 | 16S | *Mycobacterium* sp.<br>*Mycobacterium* sp.<br>*Mycobacterium lutetiense* | MG835594.1<br>MG835593.1<br>NR_151953.1 | *Mycobacterium* sp. (Corynebacteriales) | 2 |
| CKT76 | TSB3+10 | MW012369 | 16S | *Vibrio aestuarianus*<br>*Vibrio* sp.<br>*Vibrio* sp. | NR_113780.1<br>HQ449463.1<br>HM012774.1 | *Vibrio* sp. (Vibrionales) | 1 |
| CKT77 | CMN | MW012370 | 16S | *Streptomyces* sp.<br>*Streptomyces* sp.<br>*Streptomyces badius* | MK292047.1<br>MK271721.1<br>MK156399.1 | *Streptomyces* sp. (Streptomycetales) | 1 |
| CKT78 | CMB | MW017488 | ITS | *Penicillium crustosum*<br>*Penicillium crustosum*<br>*Penicillium crustosum* | MG975627.1<br>MG596635.1<br>KT876714.1 | *Penicillium crustosum* (Eurotiales) | 1 |
| CKT79 | CMB | MW012378 | 18S | *Pseudallescheria ellipsoidea*<br>*Pseudallescheria ellipsoidea*<br>*Pseudallescheria boydii* | NG_063099.1<br>U43911.1<br>U43915.1 | *Pseudallescheria* sp. (Microascales) | 2 |
| CKT80 | MA | MW012371 | 16S | *Streptomyces* sp.<br>*Streptomyces* sp.<br>*Streptomyces badius* | MK292047.1<br>MK271721.1<br>MK156399.1 | *Streptomyces* sp. (Streptomycetales) | 1 |

| Strain | Medium | Acc. no. | Amplicon | Closest related species (Blast) | Acc. no. closest related species | Lowest taxonomic classification (order) | RG |
|---|---|---|---|---|---|---|---|
| CKT81 | CMB | MW017489 | ITS | *Pithomyces chartarum*<br>Fungal sp. strain A210A<br>*Pithomyces chartarum* | MH860227.1<br>KU837820.1<br>KX664331.1 | *Pithomyces chartarum* (Pleosporales) | 1 |
| CKT84 | CMN | MW017490 | ITS | *Fusarium graminearum*<br>*Fusarium graminearum*<br>*Fusarium graminearum* | MK212898.1<br>MK212894.1<br>MK212893.1 | *Fusarium* sp. (Hypocreales) | 1 |
| CKT85 | PDA | MW017491 | ITS | *Cadophora luteo-olivacea*<br>*Cadophora luteo-olivacea*<br>*Cadophora luteo-olivacea* | MH859460.1<br>MG944391.1<br>MG944390.1 | *Cadophora luteo-olivacea* (Helotiales) | 1 |
| CKT86 | TSB3+10 | MW017492 | ITS | *Plectosphaerella cucumerina*<br>*Plectosphaerella cucumerina*<br>*Plectosphaerella cucumerina* | KT596812.1<br>KU204705.1<br>MH791266.1 | *Plectosphaerella cucumerina* (Glomerellales) | 1 |
| CKT90 | PDA | MW017493 | ITS | *Pichia* sp.<br>Uncult. ascomycete BF-OTU252<br>*Wickerhamomyces onychis* | EU877913.1<br>AM901934.1<br>KT207216.1 | *Wickerhamomyces* sp. (Saccharomycetales) | 1 |
| CKT91 | WSP30 | MW017494 | ITS | *Phoma* sp.<br>*Phoma* sp.<br>*Boeremia exigua* | MH550515.1<br>MH550514.1<br>MH859059.1 | *Boeremia exigua* (Pleosporales) | 1 |
| HW2 | MA | MW013337 | 16S | *Vibrio owensii*<br>*Vibrio owensii*<br>*Vibrio owensii* | LC369696.1<br>MG896198.1<br>MG896189.1 | *Vibrio* sp. (Vibrionales) | 1 |
| HW3 | MA | MW013338 | 16S | *Vibrio splendidus*<br>*Vibrio* sp.<br>*Vibrio anguillarum* | MH010050.1<br>MG788349.1<br>CP023433.1 | *Vibrio* sp. (Vibrionales) | 2 |
| HW4 | MA | MW013339 | 16S | *Vibrio comitans*<br>*Vibrio comitans*<br>*Vibrio comitans* | KR347260.1<br>AB681692.1<br>DQ922917.1 | *Vibrio comitans* (Vibrionales) | 1 |
| HW5 | MA | MW013340 | 16S | *Pseudoalteromonas carrageenovora*<br>*Pseudoalteromonas carrageenovora*<br>*Pseudoalteromonas arctica* | LT965929.1<br>LT965928.1<br>MG681184.1 | *Pseudoalteromonas* sp. (Alteromonadales) | 1 |
| HW6 | MA | MW013341 | 16S | *Vibrio alginolyticus*<br>*Vibrio* sp.<br>*Vibrio* sp. | CP017916.1<br>KX453212.1<br>KX453210.1 | *Vibrio* sp. (Vibrionales) | 2 |

| Strain | Medium | Acc. no. | Amplicon | Closest related species (Blast) | Acc. no. closest related species | Lowest taxonomic classification (order) | RG |
|---|---|---|---|---|---|---|---|
| HW8 | MA | MW013342 | 16S | *Vibrio* sp.<br>*Vibrio breoganii*<br>*Vibrio* sp. | MH807583.1<br>CP016177.1<br>KX197382.1 | *Vibrio* sp. (Vibrionales) | 1 |
| HW9 | MA | MW013343 | 16S | Marine bacterium I4017<br>*Vibrio pectenicida*<br>*Vibrio pectenicida* | KJ469389.1<br>NR_118241.1<br>NR_029344.1 | *Vibrio pectenicida* (Vibrionales) | 1 |
| HW10 | TSB3+10 | MW013344 | 16S | *Kocuria* sp.<br>*Kocuria palustris*<br>*Kocuria palustris* | KY296995.1<br>MF319775.1<br>KY933468.1 | *Kocuria palustris* (Micrococcales) | 1 |
| HW11 | TSB3+10 | MW013345 | 16S | *Knoellia* sp.<br>*Knoellia subterranea*<br>*Knoellia* sp. | DQ812538.1<br>NR_028932.1<br>KP191088.1 | *Knoellia subterranea* (Micrococcales) | 1 |
| HW12 | TSB3+10 | MW013346 | 16S | *Paracoccus* sp.<br>Uncult. bacterium isolate RA2-73<br>*Paracoccus alkenifer* | KU163256.1<br>KT834758.1<br>LT221244.1 | *Paracoccus alkenifer* (Rhodobacterales) | 1 |
| HW13 | TSB3+10 | MW013347 | 16S | Uncult. *Vibrio*<br>Uncult. *Vibrio*<br>*Vibrio owensii* | MG554543.1<br>MG554505.1<br>CP025797.1 | *Vibrio* sp. (Vibrionales) | 1 |
| HW14 | CMN | MW013348 | 16S | *Vibrio splendidus*<br>*Cellulophaga* sp.<br>*Cellulophaga* sp. | CP031055.1<br>JX435328.1<br>JX435323.1 | *Vibrio* sp. (Vibrionales) | 2 |
| HW15 | CMN | MW013349 | 16S | *Chryseomicrobium imtechense*<br>*Chryseomicrobium palamuruense*<br>*Chryseomicrobium* sp. | MH643668.1<br>MG461542.1<br>KX889925.1 | *Chryseomicrobium* sp. (Bacillales) | 1 |
| HW16 | CMN | MW013350 | 16S | Uncult. bacterium Shelves_A_113<br>Uncult. bacterium Shelves_A_62<br>*Corynebacterium casei* | MF092438.1<br>MF092420.1<br>KP790025.1 | *Corynebacterium casei* (Corynebacteriales) | 1 |
| HW18 | CMN | MW013351 | 16S | *Cellulophaga fucicola*<br>*Cellulophaga fucicola*<br>*Cellulophaga* sp. | KX453201.1<br>KX453191.1<br>LN881203.1 | *Cellulophaga fucicola* (Flavobacteriales) | 1 |
| HW23 | WSP30 | MW013352 | 16S | *Idiomarina* sp.<br>*Idiomarina* sp.<br>*Idiomarina loihiensis* | EF409425.1<br>EF409424.1<br>KM407721.1 | *Idiomarina loihiensis* (Alteromonadales) | 1 |

| Strain | Medium | Acc. no. | Amplicon | Closest related species (Blast) | Acc. no. closest related species | Lowest taxonomic classification (order) | RG |
|---|---|---|---|---|---|---|---|
| HW25 | TSB3+10 | MW013353 | 16S | *Staphylococcus pasteuri*<br>*Staphylococcus pasteuri*<br>*Staphylococcus pasteuri* | MG815139.1<br>MG757632.1<br>MG680735.1 | *Staphylococcus pasteuri* (Bacillales) | 2 |
| HW27 | MA | MW013354 | 16S | *Vibrio ostreicida*<br>*Vibrio ostreicida*<br>*Vibrio ostreicida* | NR_133887.1<br>EU652412.2<br>KX130913.1 | *Vibrio* sp. (Vibrionales) | 1 |
| HW28 | WSP30 | MW013355 | 16S | *Epibacterium mobile*<br>*Epibacterium mobile*<br>Bacterium strain InAD-034 | MK493584.1<br>MK493561.1<br>MF401241.1 | Rhodobacteraceae unclassified (Rhodobacterales) | 1 |
| HW30 | TSB3+10 | MW012380 | ITS | *Cryptococcus magnus*<br>*Cryptococcus* sp.<br>*Cryptococcus magnus* | JQ425367.1<br>HQ426594.1<br>EU871517.1 | *Cryptococcus magnus* (Filobasidiales) | 1 |
| HW32 | TSB3+10 | MW013356 | 16S | *Sphingomonas* sp.<br>Uncult. bacterium YD200-16<br>*Sphingomonas aquatilis* | KJ606800.1<br>JX441481.1<br>NR_024997.1 | *Sphingomonas* sp. (Sphingomonadales) | 1 |
| HW33 | TSB3+10 | MW013357 | 16S | *Staphylococcus capitis*<br>*Staphylococcus capitis*<br>Uncult. bacterium 16s_M.Zamir | MF033474.1<br>MG557816.1<br>MG461572.1 | *Staphylococcus* sp. (Bacillales) | 1 |
| HW35 | CMN | MW013358 | 16S | *Palleronia abyssalis*<br>*Palleronia abyssalis*<br>*Palleronia abyssalis* | KJ638255.1<br>MG383388.1<br>KJ638254.1 | *Palleronia abyssalis* (Rhodobacterales) | 1 |
| HW36 | CMN | MW013359 | 16S | *Erythrobacter* sp.<br>*Erythrobacter citreus*<br>*Erythrobacter citreus* | KT185356.1<br>AB012062.1<br>LN846110.1 | *Erythrobacter* sp. (Sphingomonadales) | 1 |
| HW37 | TSB3+10 | MW013360 | 16S | *Vibrio aestuarianus*<br>*Vibrio* sp.<br>*Vibrio* sp. | NR_113780.1<br>HQ449463.1<br>HM012774.1 | *Vibrio aestuarianus* (Vibrionales) | 1 |
| HW38 | PDA | MW012381, MW014884 | ITS, 18S | Uncult. fungus C2_EH11<br>Melanized limestone ascomycete CR-2004<br>*Cladophialophora chaetospira,*<br>*Cladophialophora boppii*<br>*Exophiala lecanii-corni*<br>*Fonsecaea nubica* | JX042985.1<br>HM239803.1<br>EU035403.1,<br>NG_062637.1<br>CP034379.1<br>GU197483.1 | Herpotrichiellaceae unclassified (Chaetothyriales) | 2 |

| Strain | Medium | Acc. no. | Amplicon | Closest related species (Blast) | Acc. no. closest related species | Lowest taxonomic classification (order) | RG |
|---|---|---|---|---|---|---|---|
| HW40 | WSP30 | MW012382,<br>MW014885,<br>MW017498 | ITS,<br>18S,<br>28S | *Capnodiales* sp.<br>*Capnodiales* sp.<br>*Extremus antarcticus*,<br>*Capnodiales* sp. CCFEE 5271<br>*Capnodiales* sp. CCFEE 5389<br>*Extremus antarcticus* CCFEE 451,<br>*Saxophila tyrrhenica*<br>*Saxophila tyrrhenica*<br>*Capnodiales* sp. CCFEE 5551 | KC315866.1<br>GU250338.1<br>NG_064939.1,<br>KC315866.1<br>GU250338.1<br>NG_064939.1,<br>NG_059571.1<br>KR781051.1<br>KC315879.1 | Capnodiales unclassified | 1 |
| HW41 | TSB3+10 | MW013361 | 16S | *Methylobacterium* sp.<br>*Methylobacterium* sp.<br>*Methylobacterium oryzae* | FN868948.1<br>MG807376.1<br>MF692767.1 | *Methylobacterium* sp. (Rhizobiales) | 1 |
| HW42 | TSB3+10 | MW013362 | 16S | *Vibrio owensii*<br>*Vibrio campbellii*<br>*Vibrio* sp. | MG896198.1<br>CP026321.1<br>KY655411.1 | *Vibrio* sp. (Vibrionales) | 1 |
| HW43 | TSB3+10 | MW013363 | 16S | *Rhodococcus cerastii*<br>Unidentified microorganism edSeq16_20-D3<br>Unidentified microorganism edSeq14_4-D1 | MG645219.1<br>MG271100.1<br>MG270621.1 | *Rhodococcus* sp. (Corynebacteriales) | 1 |
| HW44 | PDA | MW012383,<br>MW014886,<br>MW017499 | ITS,<br>18S,<br>28S | Uncult. fungus C2_EH11<br>Melanized limestone ascomycete CR-2004<br>*Cladophialophora chaetospira,*<br>*Cladophialophora boppii*<br>*Cladophialophora boppii*<br>*Fonsecaea nubica,*<br>*Phialophora verrucosa*<br>*Phialophora verrucosa*<br>*Phialophora verrucosa* | JX042985.1<br>AY559331.1<br>EU035403.1,<br>NG_062637.1<br>AJ232946.1<br>GU197483.1,<br>AB550778.1<br>AB550777.1<br>AB550776.1 | Herpotrichiellaceae unclassified (Chaetothyriales) | 2 |
| HW45 | PDA | MW013364 | 16S | *Methylobacterium* sp.<br>*Methylobacterium* sp.<br>*Methylobacterium oryzae* | KP128697.1<br>KF441619.1<br>MF692767.1 | *Methylobacterium* sp. (Rhizobiales) | 1 |

| Strain | Medium | Acc. no. | Amplicon | Closest related species (Blast) | Acc. no. closest related species | Lowest taxonomic classification (order) | RG |
|---|---|---|---|---|---|---|---|
| HW47 | CMN | MW012384 | ITS | *Purpureocillium lilacinum*<br>*Purpureocillium lilacinum*<br>*Purpureocillium lilacinum* | MH865347.1<br>MH865301.1<br>MH865154.1 | *Purpureocillium lilacinum* (Hypocreales) | 2 |
| HW48 | CMN | MW013365 | 16S | Uncult. bacterium A1_27<br>Uncult. bacterium A1_27<br>*Psychrobacter glacincola* | HG795730.1<br>HG795728.1<br>KU579265.1 | *Psychrobacter* sp. (Pseudomonadales) | 1 |
| KW1 | MA | MW013366 | 16S | *Psychrobacter* sp.<br>*Psychrobacter* sp.<br>*Psychrobacter maritimus* | MF537176.1<br>MF537175.1<br>MH368410.1 | *Psychrobacter* sp. (Pseudomonadales) | 1 |
| KW2 | MA | MW013367 | 16S | *Pseudoalteromonas tunicata*<br>*Pseudoalteromonas tunicata*<br>*Pseudoalteromonas* sp. | KY319053.1<br>CP011032.1<br>KX755364.1 | *Pseudoalteromonas tunicata* (Alteromonadales) | 1 |
| KW3 | MA | MW013368 | 16S | *Shewanella* sp.<br>*Shewanella* sp.<br>*Shewanella colwelliana* | MF594130.1<br>MF045123.1<br>KX756553.1 | *Shewanella colwelliana* (Alteromonadales) | 1 |
| KW4 | MA | MW013369 | 16S | *Pseudoalteromonas tunicata*<br>*Pseudoalteromonas tunicata*<br>*Pseudoalteromonas* sp. | KY319053.1<br>CP011032.1<br>KX755364.1 | *Pseudoalteromonas tunicata* (Alteromonadales) | 1 |
| KW5 | MA | MW013370 | 16S | *Pseudoalteromonas* sp.<br>*Pseudoalteromonas* sp.<br>*Pseudoalteromonas ulvae* | KU647930.1<br>MK743964.1<br>KF472191.1 | *Pseudoalteromonas* sp. (Alteromonadales) | 1 |
| KW6 | MA | MW013371 | 16S | *Lentibacter* sp. strain HYO3<br>Uncult. bacterium SEM1C041<br>*Lentibacter algarum* | KX755376.1<br>KJ094194.1<br>NR_108333.1 | *Lentibacter algarum* (Rhodobacterales) | 1 |
| KW7 | MA | MW013372 | 16S | *Pseudoalteromonas carrageenovora*<br>*Pseudoalteromonas* sp.<br>*Pseudoalteromonas* sp. | MH362718.1<br>MH333259.1<br>MF401566.1 | *Pseudoalteromonas* sp. (Alteromonadales) | 1 |
| KW8 | MA | MW013373 | 16S | *Psychrobacter glaciei*<br>*Psychrobacter fjordensis*<br>*Psychrobacter cryohalolentis* | NR_148850.1<br>NR_148330.1<br>NR_075055.1 | *Psychrobacter* sp. (Pseudomonadales) | 2 |
| KW9* | MA | MW013374 | 16S | Uncult. Flavobacteriaceae bacterium C114Chl024<br>*Mesonia algae*<br>*Mesonia algae* | JX525404.1<br>LT601221.2<br>LT601219.2 | *Mesonia* sp. (Flavobacteriales) | 1 |

| Strain | Medium | Acc. no. | Amplicon | Closest related species (Blast) | Acc. no. closest related species | Lowest taxonomic classification (order) | RG |
|---|---|---|---|---|---|---|---|
| KW10 | MA | MW013375 | 16S | *Vibrio anguillarum*<br>Uncult. bacterium 2010ECS-StA#54<br>Uncult. bacterium SanDiego_a6617 | CP011460.1<br>KM471344.1<br>KF799860.1 | Vibrio *anguillarum* (Vibrionales) | 2 |
| KW11* | CMB | MW013376 | 16S | *Cobetia marina*<br>*Halomonas* sp.<br>*Cobetia amphilecti* | MH169273.1<br>CP028367.1<br>KX418494.1 | *Cobetia* sp. (Oceanospirillales) | 1 |
| KW12 | CMB | MW013377 | 16S | *Pseudoalteromonas tunicata*<br>*Pseudoalteromonas tunicata*<br>*Pseudoalteromonas* sp. | KY319053.1<br>CP011032.1<br>KX755364.1 | *Pseudoalteromonas tunicata* (Alteromonadales) | 1 |
| KW13 | CMB | MW013378 | 16S | *Erythrobacter* sp.<br>*Erythrobacter* sp.<br>*Erythrobacter vulgaris* | MG953322.1<br>MG833278.1<br>LK391640.1 | *Erythrobacter* sp. (Sphingomonadales) | 1 |
| KW14 | CMB | MW013379 | 16S | *Arthrobacter* sp.<br>*Glutamicibacter protophormiae*<br>*Arthrobacter protophormiae* | MF801344.1<br>KX768287.1<br>KT261110.1 | *Glutamicibacter protophormiae* (Micrococcales) | 1 |
| KW15 | CMB | MW013380 | 16S | *Paracoccus* sp.<br>Uncult. bacterium ncd2046h03c1<br>*Paracoccus zhejiangensis* | FJ267566.1<br>JF168258.1<br>CP025430.1 | *Paracoccus* sp. (Rhodobacterales) | 1 |
| KW16 | CMB | MW013381 | 16S | *Erythrobacter* sp.<br>*Erythrobacter citreus*<br>*Erythrobacter citreus* | MN435731.1<br>MK254653.1<br>MK254652.1 | *Erythrobacter* sp. (Sphingomonadales) | 1 |
| KW18 | CMB | MW013382 | 16S | *Pseudoalteromonas* sp.<br>*Pseudoalteromonas* sp.<br>*Pseudoalteromonas undina* | MG388120.1<br>MF289546.1<br>KU588389.1 | *Pseudoalteromonas* sp. (Alteromonadales) | 1 |
| KW19 | CMB | MW013383 | 16S | *Psychrobacter nivimaris*<br>*Psychrobacter sp.*<br>*Psychrobacter proteolyticus* | MH978646.1<br>MG309426.1<br>LS483016.1 | *Psychrobacter* sp. (Pseudomonadales) | 1 |
| KW21 | CMN | MW013384 | 16S | *Olleya marilimosa*<br>*Olleya marilimosa*<br>*Olleya algicola* | JN175350.2<br>NR_104945.2<br>KY341922.1 | *Olleya marilimosa* (Flavobacteriales) | 1 |
| KW22 | CMN | MW013385 | 16S | *Aurantimonas coralicida*<br>*Aurantimonas manganoxydans*<br>*Aurantimonas coralicida* | NR_042319.1<br>NR_114936.1<br>NR_115134.1 | *Aurantimonas* sp. (Rhizobiales) | 1 |

| Strain | Medium | Acc. no. | Amplicon | Closest related species (Blast) | Acc. no. closest related species | Lowest taxonomic classification (order) | RG |
|---|---|---|---|---|---|---|---|
| KW23 | CMN | MW013386 | 16S | *Pseudoalteromonas carrageenovora*<br>*Pseudoalteromonas* sp.<br>*Pseudoalteromonas* sp. | MH362718.1<br>MH333259.1<br>MF401566.1 | *Pseudoalteromonas* sp. (Alteromonadales) | 1 |
| KW24 | CMN | MW013387 | 16S | *Pseudomonas* sp.<br>*Pseudomonas* sp.<br>*Pseudomonas stutzeri* | KR012328.1<br>KR012234.1<br>AJ270454.1 | *Pseudomonas stutzeri* (Pseudomonadales) | 1 |
| KW25* | CMN | MW013388 | 16S | *Mesonia algae*<br>*Mesonia algae*<br>*Mesonia algae* | LT601221.2<br>LT601219.2<br>LT601217.2 | *Mesonia* sp. (Flavobacteriales) | 1 |
| KW26 | CMN | MW013389 | 16S | *Bacillus* sp.<br>*Bacillus* sp.<br>*Bacillus pumilus* | MH411221.1<br>MH411112.1<br>MF079375.1 | *Bacillus* sp. (Bacillales) | 1 |
| KW27 | CMN | MW013390 | 16S | *Pseudoalteromonas* sp.<br>*Pseudoalteromonas marina*<br>*Pseudoalteromonas marina* | MF537048.1<br>MH362719.1<br>MH362716.1 | *Pseudoalteromonas* sp. (Alteromonadales) | 1 |
| KW28 | CMN | MW013391 | 16S | *Agrococcus* sp.<br>*Agrococcus* sp.<br>*Agrococcus baldri* | KM362887.1<br>KY476554.1<br>HF913436.1 | *Agrococcus baldri* (Micrococcales) | 1 |
| KW29 | CMN | MW013392 | 16S | Uncult. alpha proteobacterium SGSH999<br>*Sphingopyxis baekryungensis*<br>Uncult. bacterium CFL_Lb37 | GQ347702.1<br>NR_043014.1<br>KJ365389.1 | *Sphingopyxis baekryungensis* (Sphingomonadales) | 1 |
| KW30 | WSP30 | MW013393 | 16S | Uncult. bacterium Shelves_A_110<br>*Psychrobacter* sp.<br>*Psychrobacter nivimaris* | MF092435.1<br>KY382827.1<br>MH478336.1 | *Psychrobacter nivimaris* (Pseudomonadales) | 1 |
| KW31 | TSB3+10 | MW013394 | 16S | *Serinicoccus* sp.<br>*Serinicoccus* sp.<br>*Serinicoccus chungangensis* | DQ985074.1<br>KM886154.1<br>NR_117788.1 | *Serinicoccus* sp. (Micrococcales) | 1 |
| KW33 | TSB3+10 | MW013395 | 16S | *Shewanella algicola*<br>*Shewanella gelidii*<br>*Shewanella arctica* | NR_149298.1<br>NR_151921.1<br>NR_117528.1 | *Shewanella* sp. (Alteromonadales) | 2 |
| KW34 | TSB3+10 | MW013396 | 16S | *Pseudomonas* sp.<br>*Pseudomonas* sp.<br>*Pseudomonas peli* | MH109498.1<br>MH109497.1<br>MF077147.1 | *Pseudomonas* sp. (Pseudomonadales) | 1 |

| Strain | Medium | Acc. no. | Amplicon | Closest related species (Blast) | Acc. no. closest related species | Lowest taxonomic classification (order) | RG |
|---|---|---|---|---|---|---|---|
| KW36 | TSB3+10 | MW013397 | 16S | *Shewanella putrefaciens*<br>*Shewanella hafniensis*<br>*Shewanella hafniensis* | MH304320.1<br>KX271693.1<br>KX271692.1 | *Shewanella* sp. (Alteromonadales) | 2 |
| KW37 | TSB3+10 | MW013398 | 16S | *Pseudoalteromonas ulvae*<br>*Pseudoalteromonas tunicata*<br>*Pseudoalteromonas piscicida* | NR_025032.1<br>NR_029365.1<br>NR_114583.1 | *Pseudoalteromonas* sp. (Alteromonadales) | 1 |
| KW38 | TSB3+10 | MW013399 | 16S | *Arthrobacter echini*<br>*Arthrobacter echini*<br>*Arthrobacter* sp. | NR_148833.1<br>KJ789956.1<br>FR693359.1 | *Arthrobacter echini* (Micrococcales) | 2 |
| KW39* | WSP30 | MW013400 | 16S | *Cobetia* sp.<br>*Cobetia* sp.<br>*Cobetia litoralis* | LN881270.1<br>LN881234.1<br>AB646235.1 | *Cobetia* sp. (Oceanospirillales) | 1 |
| KW40 | MA | MW013401 | 16S | *Vibrio* sp.<br>*Vibrio* sp.<br>*Vibrio anguillarum* | MF537054.1<br>MF537053.1<br>CP022468.1 | *Vibrio* sp. (Vibrionales) | 2 |
| KW41 | MA | MW013402 | 16S | *Pseudoalteromonas agarivorans*<br>*Pseudoalteromonas prydzensis*<br>*Pseudoalteromonas carrageenovora* | MH362723.1<br>MH362721.1<br>MH362718.1 | *Pseudoalteromonas* sp. (Alteromonadales) | 1 |
| KW42 | TSB3+10 | MW013403 | 16S | *Planococcus* sp.<br>*Planococcus maritimus*<br>*Planococcus maritimus* | KX645673.1<br>MF405217.1<br>MF276799.1 | *Planococcus* sp. (Bacillales) | 1 |
| KW43 | WSP30 | MW013404 | 16S | *Arthrobacter echini*<br>*Arthrobacter echini*<br>*Arthrobacter* sp. | NR_148833.1<br>KJ789956.1<br>FR693359.1 | *Arthrobacter echini* (Micrococcales) | 2 |
| KW44 | WSP30 | MW013405 | 16S | *Exiguobacterium* sp.<br>*Exiguobacterium* sp.<br>*Exiguobacterium aurantiacum* | MF537096.1<br>MF537083.1<br>KY196514.1 | *Exiguobacterium* sp. (Bacillales) | 1 |
| KW45 | WSP30 | MW013406 | 16S | *Sulfitobacter dubius*<br>*Sulfitobacter dubius*<br>*Sulfitobacter* sp. | MH725548.1<br>MH725547.1<br>MG210570.1 | *Sulfitobacter dubius* (Rhodobacterales) | 1 |
| KW46 | WSP30 | MW013407 | 16S | *Rhodococcus* sp.<br>*Rhodococcus* sp.<br>*Rhodococcus cerastii* | MH173295.1<br>KY405925.2<br>MG645219.1 | *Rhodococcus* sp. (Corynebacteriales) | 1 |

| Strain | Medium | Acc. no. | Amplicon | Closest related species (Blast) | Acc. no. closest related species | Lowest taxonomic classification (order) | RG |
|---|---|---|---|---|---|---|---|
| KW47 | WSP30 | MW013408 | 16S | *Shewanella baltica*<br>*Shewanella baltica*<br>*Shewanella putrefaciens* | MH304331.1<br>MH304326.1<br>MH304324.1 | *Shewanella* sp. (Alteromonadales) | 2 |
| KW48 | CMN | MW013409 | 16S | *Pseudoalteromonas tunicata*<br>*Pseudoalteromonas tunicata*<br>*Pseudoalteromonas* sp. | CP031961.1<br>KY319053.1<br>CP011032.1 | *Pseudoalteromonas tunicata* (Alteromonadales) | 1 |
| KW49 | WSP30 | MW012385 | ITS | *Candida sequanensis*<br>*Candida sequanensis*<br>*Candida sequanensis* | FM178365.1<br>NR_111302.1<br>KM435341.1 | *Candida sequanensis* (Saccharomycetales) | 2 |
| KW50* | WSP30 | MW013410 | 16S | *Cobetia* sp.<br>*Cobetia* sp.<br>*Cobetia litoralis* | LN881270.1<br>LN881234.1<br>AB646235.1 | *Cobetia* sp. (Oceanospirillales) | 1 |
| KW51 | WSP30 | MW013411 | 16S | *Rhodococcus* sp.<br>*Rhodococcus* sp.<br>*Rhodococcus cerastii* | MH236179.1<br>KY405925.2<br>MG645219.1 | *Rhodococcus* sp. (Corynebacteriales) | 1 |
| KW52 | WSP30 | MW013412 | 16S | *Halomonas sulfidaeris*<br>*Halomonas titanicae*<br>*Halomonas titanicae* | NR_027185.1<br>NR_116997.1<br>NR_117300.1 | *Halomonas sulfidaeris* (Oceanospirillales) | 1 |
| KW53 | WSP30 | MW013413 | 16S | *Vibrio porteresiae*<br>*Vibrio porteresiae*<br>Uncult. *Vibrio* sp. 12L_112 | HM749744.1<br>NR_044248.1<br>KP183078.1 | *Vibrio porteresiae* (Vibrionales) | 1 |
| KW54 | WSP30 | MW013414 | 16S | *Alteromonas* sp.<br>Uncult. bacterium SS-23C02<br>*Alteromonas stellipolaris* | MF443678.1<br>KX177874.1<br>CP015345.1 | *Alteromonas* sp. (Alteromonadales) | 1 |
| KW55 | CMB | MW013415 | 16S | *Pseudoalteromonas tunicata*<br>*Pseudoalteromonas tunicata*<br>*Pseudoalteromonas* sp. | KY319053.1<br>CP011032.1<br>KX755364.1 | *Pseudoalteromonas tunicata* (Alteromonadales) | 1 |
| KW56 | CMN | MW013416 | 16S | Uncult. bacterium CFL_Lb37<br>*Sphingopyxis baekryungensis*<br>*Sphingopyxis baekryungensis* | KJ365389.1<br>HF913434.1<br>HE800827.1 | *Sphingopyxis baekryungensis* (Sphingomonadales) | 1 |
| KW57 | TSB3+10 | MW013417 | 16S | *Okibacterium* sp.<br>*Okibacterium* sp.<br>*Mycetocola zhadangensis* | KU507611.1<br>HM224472.1<br>NR_109597.1 | Microbacteriaceae unclassified (Micrococcales) | 1 |
| KW58 | TSB3+10 | MW013418 | 16S | *Microbacterium phyllosphaerae*<br>*Microbacterium foliorum*<br>*Microbacterium* sp. | MF541529.1<br>MG195155.1<br>MF458881.1 | *Microbacterium* sp. (Micrococcales) | 1 |

| Strain | Medium | Acc. no. | Amplicon | Closest related species (Blast) | Acc. no. closest related species | Lowest taxonomic classification (order) | RG |
|---|---|---|---|---|---|---|---|
| KW60 | CMN | MW013419 | 16S | *Vibrio cortegadensis*<br>*Vibrio cyclitrophicus*<br>*Vibrio cyclitrophicus* | NR_148247.1<br>NR_115806.1<br>NR_042467.1 | *Vibrio* sp. (Vibrionales) | 1 |
| KW61 | WSP30 | MW012386 | ITS | *Penicillium* sp.<br>*Penicillium* sp.<br>*Penicillium chrysogenum* | MK268129.1<br>MK267794.1<br>MH048884.1 | *Penicillium* sp. (Eurotiales) | 1 |
| KW63 | MA | MW013420 | 16S | *Shewanella* sp.<br>*Shewanella* sp.<br>*Shewanella colwelliana* | MF045122.1<br>MF045121.1<br>KX756553.1 | *Shewanella colwelliana* (Alteromonadales) | 1 |
| KW65 | CMN | MW013421 | 16S | *Serinicoccus* sp.<br>*Serinicoccus* sp.<br>*Serinicoccus chungangensis* | KP872112.1<br>KM886155.1<br>NR_117788.1 | *Serinicoccus* sp. (Micrococcales) | 1 |
| KW66 | CMN | MW013422 | 16S | *Erythrobacter* sp.<br>*Erythrobacter* sp.<br>*Erythrobacter litoralis* | KX989363.1<br>KX989361.1<br>KY047411.1 | *Erythrobacter* sp. (Sphingomonadales) | 1 |
| KW67 | CMN | MW013423 | 16S | *Arthrobacter agilis*<br>*Arthrobacter agilis*<br>*Arthrobacter* sp. | LT984721.1<br>CP024915.1<br>MG860335.1 | *Arthrobacter* sp. (Micrococcales) | 1 |
| KW68 | CMN | MW013424 | 16S | *Psychrobacter* sp.<br>*Psychrobacter* sp.<br>*Psychrobacter nivimaris* | MG309426.1<br>MH707184.1<br>MH478336.1 | *Psychrobacter nivimaris* (Pseudomonadales) | 1 |
| KW69 | TSB3+10 | MW013425 | 16S | *Arthrobacter echini*<br>*Arthrobacter echini*<br>*Arthrobacter* sp. | NR_148833.1<br>KJ789956.1<br>FR693359.1 | *Arthrobacter echini* (Micrococcales) | 2 |
| KW71 | TSB3+10 | MW013426 | 16S | *Rhodococcus* sp.<br>*Rhodococcus* sp.<br>*Rhodococcus cerastii* | MH236179.1<br>KY405925.2<br>MG645219.1 | *Rhodococcus* sp. (Corynebacteriales) | 1 |
| KW72 | WSP30 | MW013427 | 16S | *Aurantimonas coralicida*<br>*Aurantimonas litoralis*<br>*Aurantimonas manganoxydans* | MH725320.1<br>KR140222.1<br>LC066380.1 | *Aurantimonas* sp. (Rhizobiales) | 1 |
| KW74 | WSP30 | MW012387 | ITS | *Dendrophoma cytisporoides*<br>*Dendrophoma cytisporoides*<br>Uncult. fungus OTU_F382_R420 | NR_153978.1<br>JQ889273.1<br>MF976669.1 | *Dendrophoma cytisporoides* (Chaetosphaeriales) | 1 |
| KW75 | WSP30 | MW014887 | 18S | *Sakaguchia lamellibrachiae*<br>*Sakaguchia lamellibrachiae*<br>*Symmetrospora marina* | AB126646.1<br>AB263120.1<br>KJ806313.1 | *Sakaguchia lamellibrachiae* (Sakaguchiales) | 2 |

| Strain | Medium | Acc. no. | Amplicon | Closest related species (Blast) | Acc. no. closest related species | Lowest taxonomic classification (order) | RG |
|---|---|---|---|---|---|---|---|
| KW76 | WSP30 | MW013428 | 16S | *Pseudoalteromonas agarivorans*<br>*Pseudoalteromonas carrageenovora*<br>*Pseudoalteromonas* sp. | MH362723.1<br>MH362718.1<br>MH333259.1 | *Pseudoalteromonas* sp. (Alteromonadales) | 1 |

**Table S3. Bioactivity (%) of crude extracts derived from tunic-associated microbial strains at** a **test concentration of 100 µg/mL.** Values are given as average values of the two biological replicates, which were tested twice (technical replicate). Test organisms and cell lines are abbreviated as follows: MRSA: Methicillin-resistant *Staphylococcus aureus*, Efm: *Enterococcus faecium*, Ca: *Candida albicans*, Cn: *Cryptococcus neoformans*, A375: Malignant melanoma, A549: Lung carcinoma, HCT116: Colon cancer, MB231: Breast cancer. Several bacterial strains did not grow on GYM medium and hence, were only cultivated on MB medium. "-": Inhibition values ≤ 20%. Bold: Inhibition values ≥ 80%.

| Strain | Lowest taxonomic classification | Medium | MRSA | Efm | Ca | Cn | A375 | A549 | HCT116 | MB231 |
|---|---|---|---|---|---|---|---|---|---|---|
| CHT3 | *Leisingera aquimarina* | GYM | - | - | - | 21 | - | - | - | 22 |
| | | MB | **80** | - | - | - | - | - | - | - |
| CHT5 | *Shewanella* sp. | MB | 36 | - | - | - | - | - | - | - |
| CHT6 | *Vibrio gigantis* | MB | 60 | - | - | - | - | - | - | - |
| CHT7 | *Ruegeria faecimaris* | MB | 35 | - | - | - | - | - | - | - |
| CHT9 | *Vibrio* sp. | MB | - | - | - | - | - | - | - | - |
| CHT10 | *Kangiella* sp. | MB | **100** | **99** | - | - | - | - | - | - |
| CHT13 | *Aurantimonas* sp. | GYM | 46 | - | - | - | - | - | - | - |
| | | MB | **85** | - | - | - | - | - | - | - |
| CHT15 | *Bacillus* sp. | GYM | 54 | 44 | - | 48 | - | - | - | - |
| | | MB | 61 | - | - | - | - | - | - | - |
| CHT17 | *Vibrio* sp. | MB | 70 | - | - | - | - | - | - | - |
| CHT22a | *Marixanthomonas ophiurae* | MB | 52 | - | - | - | - | - | - | - |
| CHT22b | *Vibrio* sp. | MB | **100** | - | - | - | - | - | - | - |
| CHT23 | *Amphritea spongicola* | MB | **91** | **100** | - | - | - | - | - | - |
| CHT25 | *Pseudorhodobacter aquimaris* | MB | 74 | - | - | - | - | - | - | - |
| CHT27 | *Bacillus* sp. | GYM | 42 | 39 | - | 37 | - | - | - | - |
| | | MB | 44 | - | - | - | - | - | - | - |
| CHT29 | *Arenibacter* sp. | MB | - | - | - | - | - | - | - | - |
| CHT30 | *Micrococcus* sp. | GYM | **100** | **100** | - | 32 | - | - | - | - |
| | | MB | 42 | - | - | 37 | 20 | - | - | - |
| CHT35 | *Cladosporium* sp. | CAG | **92** | 38 | - | - | - | - | - | - |
| | | PDA | **85** | - | - | - | - | - | - | - |
| CHT37 | *Emericellopsis maritima* | CAG | - | - | - | - | 28 | 45 | - | - |
| | | PDA | - | - | - | - | 56 | 57 | 72 | **81** |
| CHT40 | *Pseudochaetosphaeronema larense* | CAG | - | - | - | - | - | - | - | - |
| | | PDA | 50 | - | - | - | 63 | - | 40 | 59 |
| CHT41 | *Mycolicibacterium* sp. | MB | - | - | - | - | - | - | - | - |
| CHT42 | *Ruegeria* sp. | MB | 23 | - | - | 29 | 59 | - | 30 | - |

| Strain | Lowest taxonomic classification | Medium | MRSA | Efm | Ca | Cn | A375 | A549 | HCT116 | MB231 |
|---|---|---|---|---|---|---|---|---|---|---|
| CHT43 | *Primorskyibacter* sp. | MB | 66 | - | - | - | - | - | - | - |
| CHT46 | *Litoreibacter* sp. | MB | 75 | - | - | - | - | - | - | - |
| CHT47 | *Roseovarius arcticus* | MB | 66 | - | - | - | - | - | - | - |
| CHT48 | *Vibrio* sp. | MB | 52 | - | - | - | - | - | - | - |
| CHT49 | *Ruegeria atlantica* | MB | 60 | - | - | - | - | - | - | - |
| CHT53 | *Ochrobactrum pseudogrignonense* | GYM | 60 | - | - | - | - | - | - | - |
| | | MB | 76 | - | - | - | - | - | - | - |
| CHT54 | *Bacillus* sp. | GYM | 33 | 67 | - | 38 | - | - | - | - |
| | | MB | 61 | - | - | - | - | - | - | - |
| CHT55 | *Ochrobactrum grignonense* | GYM | **86** | - | - | - | - | - | - | - |
| | | MB | 58 | - | - | - | - | - | - | - |
| CHT56 | *Pseudogymnoascus destructans* | CAG | **100** | **100** | - | 31 | 30 | - | - | **81** |
| | | PDA | **88** | - | - | - | - | - | - | - |
| CHT58 | *Pyrenochaeta* sp. | CAG | **99** | 43 | 36 | 44 | - | - | 48 | - |
| | | PDA | **100** | **99** | **99** | **87** | 23 | 33 | 62 | 36 |
| CKT1 | *Pseudomonas* sp. | MB | 49 | - | - | - | - | - | - | - |
| CKT2 | *Litoreibacter* sp. | MB | 59 | - | - | - | - | - | - | - |
| CKT3 | *Pelagicola* sp. | MB | 47 | - | - | - | - | - | - | - |
| CKT4 | *Neptunomonas concharum* | MB | **85** | 72 | - | - | - | - | - | - |
| CKT6 | *Flaviramulus ichthyoenteri* | MB | - | - | - | - | - | - | - | - |
| CKT10 | *Pseudomonas* sp. | MB | **99** | **97** | - | - | - | - | - | - |
| CKT11 | *Vibrio aestuarianus* | MB | 59 | 27 | - | - | - | - | - | - |
| CKT12 | *Pseudomonas* sp. | GYM | 79 | - | - | - | - | - | - | - |
| | | MB | 48 | - | - | - | - | - | - | - |
| CKT16 | *Hydrogenophaga crassostreae* | GYM | **100** | **100** | - | 33 | - | - | - | - |
| | | MB | **100** | **100** | - | - | - | - | - | - |
| CKT17 | *Lysobacter spongiicola* | GYM | **100** | **100** | - | 26 | - | - | - | - |
| | | MB | **100** | **100** | - | - | - | - | - | - |
| CKT20 | *Bacillus* sp. | GYM | 46 | 46 | - | 30 | - | - | - | - |
| | | MB | 27 | - | - | - | - | - | - | - |
| CKT21 | *Pseudomonas* sp. | GYM | 74 | - | - | - | 41 | 23 | 34 | 39 |
| | | MB | 74 | - | - | - | - | - | - | - |
| CKT22 | *Phaeobacter arcticus* | MB | 73 | - | - | - | - | - | - | - |
| CKT23 | *Shewanella* sp. | MB | **100** | **100** | - | - | - | - | - | - |
| CKT24 | *Arenibacter echinorum* | MB | - | - | - | - | - | - | - | - |
| CKT28 | *Cyphellophora reptans* | CAG | **98** | - | - | - | - | - | - | - |

| Strain | Lowest taxonomic classification | Medium | MRSA | Efm | Ca | Cn | A375 | A549 | HCT116 | MB231 |
|---|---|---|---|---|---|---|---|---|---|---|
| | | PDA | 70 | - | - | - | - | - | - | - |
| CKT29 | *Marinobacter litoralis* | MB | **97** | **100** | - | - | - | - | - | - |
| CKT30 | *Salinimicrobium* sp. | MB | - | - | - | - | - | - | - | - |
| CKT34 | *Arthrobacter* sp. | GYM | **90** | **100** | - | - | - | - | 25 | - |
| | | MB | - | - | - | - | - | 30 | - | - |
| CKT35 | *Penicillium* sp. | CAG | **98** | 51 | **100** | 76 | 42 | - | 33 | 22 |
| | | PDA | **99** | **100** | **100** | 76 | 34 | - | 29 | - |
| CKT38 | *Bacillus* sp. | GYM | **88** | **100** | - | 70 | - | - | - | 25 |
| | | MB | 55 | 50 | - | - | - | - | - | - |
| CKT39 | *Streptomyces* sp. | GYM | **100** | **100** | - | 31 | - | - | - | - |
| | | MB | **95** | 45 | - | 25 | 55 | - | 33 | - |
| CKT41 | *Bacillus* sp. | GYM | **100** | 35 | - | - | - | - | - | - |
| | | MB | **99** | **92** | - | - | - | - | - | - |
| CKT43 | *Streptomyces* sp. | GYM | **100** | **100** | **91** | **100** | **98** | **89** | **97** | **95** |
| | | MB | **100** | **99** | 56 | **100** | 43 | 51 | 49 | 49 |
| CKT49 | *Penicillium brasilianum* | CAG | 46 | - | - | - | 55 | 45 | 53 | 50 |
| | | PDA | **99** | **100** | **98** | 47 | 41 | 20 | 44 | 36 |
| CKT51-II | *Bacillus* sp. | GYM | - | - | - | 41 | - | - | - | - |
| | | MB | 66 | 38 | - | - | - | - | - | - |
| CKT52 | *Bizionia fulviae* | GYM | 71 | 72 | - | - | - | - | 25 | - |
| | | MB | **95** | **100** | - | - | - | - | - | - |
| CKT56 | *Ruegeria atlantica* | MB | 49 | - | - | - | - | - | - | - |
| CKT60 | *Kiloniella laminariae* | MB | **85** | **86** | - | - | - | - | 38 | - |
| CKT65 | *Salegentibacter* sp. | MB | - | - | 22 | - | - | - | - | - |
| CKT67 | *Serinicoccus* sp. | GYM | **99** | **97** | - | 49 | - | - | - | - |
| | | MB | - | - | - | 23 | 20 | - | - | - |
| CKT78 | *Penicillium crustosum* | CAG | - | - | - | - | - | - | 21 | 27 |
| | | PDA | - | - | - | - | - | - | - | 21 |
| CKT81 | *Pithomyces chartarum* | CAG | **85** | **93** | **99** | 65 | 23 | 27 | 24 | 49 |
| | | PDA | **100** | **97** | **100** | 65 | 43 | 48 | 35 | 73 |
| CKT84 | *Fusarium* sp. | CAG | **98** | **97** | **88** | 52 | 45 | 55 | 30 | 35 |
| | | PDA | **100** | **98** | **100** | 75 | 51 | 65 | 42 | 47 |
| CKT85 | *Cadophora luteo-olivacea* | CAG | - | 25 | - | - | **90** | 78 | **87** | **84** |
| | | PDA | - | - | - | - | 35 | - | 21 | 21 |
| CKT86 | *Plectosphaerella cucumerina* | CAG | **100** | **100** | - | - | 34 | 21 | 32 | 20 |
| | | PDA | 70 | - | - | - | 37 | 22 | - | 33 |

| Strain | Lowest taxonomic classification | Medium | MRSA | Efm | Ca | Cn | A375 | A549 | HCT116 | MB231 |
|---|---|---|---|---|---|---|---|---|---|---|
| CKT90 | *Wickerhamomyces* sp. | CAG | **83** | **98** | - | - | - | - | - | - |
| | | PDA | **100** | 44 | - | - | - | - | - | - |
| CKT91 | *Boeremia exigua* | CAG | - | - | - | - | 62 | **82** | 63 | 78 |
| | | PDA | **100** | 77 | 25 | 22 | **85** | **86** | **78** | **86** |

**Table S4. Bioactivity-based selection criterion for the prioritization of extracts for in-depth chemical analyses.** A high bioactivity threshold was applied, i.e. extracts are considered active against a test strain or cancer cell line if ≥ 80% inhibitory activity at a test concentration of 100 µg/mL was observed. For each possible combination of observed bioactivities, it is indicated, whether this combination led to the selection of an extract (yes) or not (no). Activities are defined as follows: antibacterial: activity against MRSA and *E. faecium*; antifungal: activity against at least one pathogenic yeast (*C. albicans* or *C. neoformans*); antimicrobial: antibacterial + antifungal activity (as defined before); anticancer: activity against at least one of the four cancer cell lines (A375, A549, HCT116, MB231).

| **Observed bioactivities** | | | **Selected?** |
|---|---|---|---|
| **Antimicrobial** | | **Anticancer** | |
| **Antibacterial** | **Antifungal** | | |
| Yes | Yes | Yes | Yes (antimicrobial + anticancer) |
| Yes | Yes | No | Yes (antimicrobial) |
| Yes | No | No | No |
| Yes | No | Yes | Yes (anticancer) |
| No | Yes | Yes | Yes (anticancer) |
| No | Yes | No | No |
| No | No | Yes | Yes (anticancer) |
| No | No | No | No |

**Table S5. ANOSIM comparison of chemically different extracts.** ANOSIM (Euclidean distance) was computed to statistically verify differential clustering of the extracts CHT58-CAG (*Pyrenochaeta* sp.), CKT35-PDA (*Penicillium* sp.), CKT91-CAG and CKT91-PDA (*Boeremia exigua*). Remaining: extracts CHT37-PDA (*Emericellopsis maritima*), CHT56-CAG (*Pseudogymnoascus destructans*), CKT49-PDA (*Penicillium brasilianum*), CKT81-CAG and CKT81-PDA (*Pithomyces chartarum*), CKT84-CAG and CKT84-PDA (*Fusarium* sp.) and CKT85-CAG (*Cadophora luteo-olivacea*).

| **Compared groups** | **R value** | **p value** |
|---|---|---|
| All | 0.7849 | 0.0001 |
| CHT58-PDA x remaining | 0.9824 | 0.0069 |
| CKT35-PDA x remaining | 0.8843 | 0.0064 |
| CKT91-CAG and CKT91-PDA x remaining | 0.5588 | 0.0071 |

**Table S6. Putative annotation of metabolites detected in the crude extract of *Pyrenochaeta* sp. strain CHT58 cultivated on PDA medium.** Each detected compound is given with the experimentally determined *m/z* value and the predicted putative molecular formula. Putative identifications were based on the accurate mass, predicted putative molecular formulae, the retention time ($R_t$ in min), the fragmentation pattern and biological origin. *Only putative molecular formula with best ppm shown (more than 1 molecular formula possible). $^{\Delta}$Different isomers with same *m/z* value and molecular formula, which cannot be differentiated based on MS/MS data. IC: Identification confidence level after Sumner et al. 2007 [3]. Nf: No fragmentation pattern detected or fragmentation below noise threshold of $5e^1$. n.a. = putatively novel compound (known NPs do not match). Ref = reference.

| No. | *m/z* value | $R_t$ (min) | Adduct | Putative molecular formula | IC | ppm | Fragmentation pattern | Putative identification | Chemical family | Biological origin | Ref |
|---|---|---|---|---|---|---|---|---|---|---|---|
| 1 | 377.1941 | 3.17 | $[M+Na]^+$ | $C_{19}H_{30}O_6$ | 4 | 0.3 | Nf | n.a. | | | |
| 2 | 309.0978 | 3.24 | $[M+H]^+$ | $C_{15}H_{16}O_7$ | 3 | 1.3 | 291.0882, 273.0760, 255.0653, 245.0816, 227.0710, 221.0426, 181.0141, 93.0706 | Ascolactone A or B | Phthalide derivative | *Ascochyta salicorniae* (fungus) | [4] |
| 3 | 290.1008 | 3.36 | $[M+H]^+$ | $C_{11}H_{11}N_7O_3$ | 4 | 2.1 | 272.0908, 245.0808 | n.a. | | | |
| 4 | 377.1938 | 3.47 | $[M+Na]^+$ | $C_{19}H_{30}O_6$ | 4 | -0.5 | Nf | n.a. | | | |
| 5 | 373.1993 | 3.62 | $[M+Na]^+$ | $C_{20}H_{30}O_5$ | 3 | 0.6 | Nf | Aphidicolin A9 | Diterpenoid | *Botryotinia fuckeliana* (fungus) | [5] |
| 6 | 321.0981 | 3.85 | $[M+H]^+$ | $C_{16}H_{16}O_7$ | 3 | 2.2 | 303.0897, 289.0728, 271.0616, 243.0652, 254.0514, 243.0652, 229.0875, 227.0695, 217.0504, 207.0651, 151.0392 | 10-deoxybostrycin | Anthraquinone derivative | *Nigrospora* sp. (fungus) | [6] |
| 7 | 335.2215 | 3.96 | $[M+H]^+$ | $C_{20}H_{30}O_4$ | 3 | -2.1 | Nf | Aphidicolin A58 | Diterpenoid | *Botryotinia fuckeliana* (fungus) | [7] |
| 8 | 333.2066 | 4.04, 4.46, 5.7, 6.3, 7.78$^{\Delta}$ | $[M+H]^+$ | $C_{20}H_{28}O_4$ | 3 | 0 | 315.2002, 297.1773, 269.1871, 243.1402, 225.1272 | Aphidicolin A63 | Diterpenoid | *Botryotinia fuckeliana* (fungus) | [8] |
| 9 | 335.2215 | 4.23 | $[M+H]^+$ | $C_{20}H_{30}O_4$ | 3 | -2.1 | Nf | Aphidicolin A33 | Diterpenoid | *Botryotinia fuckeliana* (fungus) | [5] |
| 10 | 335.2227 | 4.31 | $[M+H]^+$ | $C_{20}H_{30}O_4$ | 3 | 1.5 | Nf | Aphidicolin A38 | Diterpenoid | *Botryotinia fuckeliana* (fungus) | [5] |
| 11 | 359.1836 | 4.58 | $[M+H]^+$ | $C_{17}H_{22}N_6O_3$ | 4 | 1.1 | 317.1724 | n.a. | | | |
| 12 | 387.2141 | 4.73 | $[M+Na]^+$ | $C_{21}H_{32}O_5$ | 3 | -1.5 | Nf | Aphidicolin A70 | Diterpenoid | *Botryotinia fuckeliana* (fungus) | [5] |
| 13 | 359.2191 | 5 | $[M+Na]^+$ | $C_{20}H_{32}O_4$ | 3 | -1.9 | Nf | Aphidicolin A35 | Diterpenoid | *Botryotinia fuckeliana* (fungus) | [5] |
| 14 | 373.1984 | 5 | $[M+Na]^+$ | $C_{20}H_{30}O_5$ | 3 | -1.9 | Nf | Aphidicolin A11 | Diterpenoid | *Botryotinia fuckeliana* (fungus) | [5] |
| 15 | 303.2327 | 5.16 | $[M+H]^+$ | $C_{20}H_{30}O_2$ | 3 | 1 | 285.2225, 267.2115, 173.1342, 161.1338, 159.1189, 149.1324, 145.0996, 133.1012, 121.1028, 119.0856, 109.1018 | Wentinoid C or D | Diterpenoid | *Aspergillus wentii* (fungus) | [7] |

| No. | *m/z* value | $R_t$ (min) | Adduct | Putative molecular formula | IC | ppm | Fragmentation pattern | Putative identification | Chemical family | Biological origin | Ref |
|---|---|---|---|---|---|---|---|---|---|---|---|
| 16 | 357.2038 | 5.24 | $[M+Na]^+$ | $C_{20}H_{30}O_4$ | 3 | -1.1 | Nf | Aphidicolin A54 | Diterpenoid | *Botryotinia fuckeliana* (fungus) | [5] |
| 17 | 359.2196 | 5.32 | $[M+Na]^+$ | $C_{20}H_{32}O_4$ | 3 | -0.6 | Nf | Aphidicolin A46 | Diterpenoid | *Botryotinia fuckeliana* (fungus) | [5] |
| 18 | 335.222 | 5.32 | $[M+H]^+$ | $C_{20}H_{30}O_4$ | 3 | -0.6 | 299.1965, 289.2251, 271.2039, 257.1883, 253.1990, 229.1680, 227.1432, 219.1374, 213.1630, 205.1536, 203.1424, 199.1478, 189.1649, 185.1324, 175.1089, 173.1331, 171.1170, 167.1059, 161.1303, 159.1160, 157.1013, 149.0942, 147.1154, 145.1038, 139.1182, 137.0944, 135.1199, 133.1020, 131.0872, 123.1195, 121.1002, 119.0865, 111.0840, 109.1014, 107.0857, 93.0710 | Aphidicolin A37 | Diterpenoid | *Botryotinia fuckeliana* (fungus) | [5] |
| 19 | 319.2271 | 5.47 | $[M+H]^+$ | $C_{20}H_{30}O_3$ | 3 | -0.6 | 275.0874, 233.0437, 189.1269, 167.0352, 149.0978, 93.0702 | Wentinoid F | Diterpenoid | *Aspergillus wentii* (fungus) | [7] |
| 20 | 335.2227 | 5.55 | $[M+H]^+$ | $C_{20}H_{30}O_4$ | 3 | 0.6 | Nf | Aphidicolin A41 | Diterpenoid | *Botryotinia fuckeliana* (fungus) | [5] |
| 21 | 317.2123 | 5.62 | $[M+H]^+$ | $C_{20}H_{28}O_3$ | 3 | 1.9 | Nf | n.a. | | | |
| 22 | 374.2335 | 5.63 | $[M+H]^+$ | $C_{22}H_{31}NO_4$* | 4 | 1.1 | 356.2203, 338.2126, 224.1295, 222.1142, 133.1017 | n.a. | | | |
| 23 | 376.2492 | 5.78 | $[M+H]^+$ | $C_{22}H_{33}NO_4$ | 2 | 1.1 | 358.2390, 340.2285, 314.2489, 253.1965, 241.1965 | Periconiasin I | Cytochalasan | *Periconia* sp. (fungus) | [7] |
| 24 | 373.1996 | 5.92 | $[M+Na]^+$ | $C_{20}H_{30}O_5$ | 3 | 1.3 | 358.2399, 340.2212, 314.2465, 260.1649, 241.1958 | Aphidicolin A61 | Diterpenoid | *Botryotinia fuckeliana* (fungus) | [5] |
| 25 | 403.2103 | 6.15 | $[M+Na]^+$ | $C_{21}H_{32}O_6$ | 3 | 1.5 | Nf | n.a. | | | |
| 26 | 331.1918 | 6.15 | $[M+H]^+$ | $C_{20}H_{26}O_4$ | 3 | 2.7 | 285.1854, 271.1707, 255.1385, 253.1597, 246.1269, 243.1754, 227.1445, 191.0710, 185.0972, 175.0763, 163.0763 | Hawaiinolide B | Diterpenoid | *Paraconiothyrium hawaiiense* (fungus) | [9] |
| 27 | 359.2206 | 6.22 | $[M+Na]^+$ | $C_{20}H_{32}O_4$ | 3 | 2.2 | Nf | Aphidicolin A48 | Diterpenoid | *Botryotinia fuckeliana* (fungus) | [5] |
| 28 | 583.1831 | 6.22 | $[M+H]^+$ | $C_{30}H_{30}O_{12}$ | 3 | 2.6 | 511.1395, 493.1300, 465.1280, 423.1118, 405.1028, 389.1071, 361.1062, 345.1097, 333.1063, 301.1031, 283.0966, 273.0782, 269.0845, 257.0845, 255.0650, 245.0871, 235.0582, 231.0632, 229.0855, 227.0734, 219.0664, 191.0723, 167.0353, 161.0597, 151.0392, 123.0456 | Talarodilactone B | Macrolide | *Talaromyces rugulosus* (fungus) | [10] |
| 29 | 335.2208 | 6.54 | $[M+H]^+$ | $C_{20}H_{30}O_4$ | 3 | -4.2 | Nf | Aphidicolin A64 | Diterpenoid | *Botryotinia fuckeliana* (fungus) | [5] |
| 30 | 402.2286 | 6.54 | $[M+H]^+$ | $C_{23}H_{31}NO_5$ | 3 | 1.5 | 384.2193, 374.2318, 356.2223, 338.2108, 241.1943 | CJ-16,264 | Pyrrolizidine alkaloid | Unknown fungus | [11] |
| 31 | 387.2153 | 6.69 | $[M+Na]^+$ | $C_{21}H_{32}O_5$ | 3 | 1.5 | Nf | Brassicicene F | Diterpenoid | *Alternaria brassicicola* (fungus) | [12] |

| No. | *m/z* value | $R_t$ (min) | Adduct | Putative molecular formula | IC | ppm | Fragmentation pattern | Putative identification | Chemical family | Biological origin | Ref |
|---|---|---|---|---|---|---|---|---|---|---|---|
| 32 | 319.2279 | 6.8 | $[M+H]^+$ | $C_{20}H_{30}O_3$ | 3 | 1.9 | 301.2173, 291.2337, 283.2069, 273.2217, 255.2120, 211.1494, 193.1600, 189.1641, 185.1329, 175.1488, 173.1328, 171.1180, 165.0913, 163.1123, 161.1332, 159.1173, 157.1016, 149.1327, 147.1174, 145.1021, 137.1330, 135.1169, 133.1013, 123.1166, 121.1021, 119.0860, 109.1019, 107.0860, 105.0706, 93.0700 | Aphidicolin A57 | Diterpenoid | *Botryotinia fuckeliana* (fungus) | [5] |
| 33 | 317.2125 | 6.95 | $[M+H]^+$ | $C_{20}H_{28}O_3$ | 3 | 2.5 | 317.2117, 300.2907, 282.2806, 270.2800, 243.1767, 201.1281, 189.1275, 173.1335, 159.1172, 145.1019, 133.1013, 119.0860, 95.0864 | n.a. | | | |
| 34 | 351.2173 | 7.11 | $[M+H]^+$ | $C_{20}H_{30}O_5$ | 3 | 0.6 | Nf | Aphidicolin A53 | Diterpenoid | *Botryotinia fuckeliana* (fungus) | [5] |
| 35 | 317.2119 | 7.17 | $[M+H]^+$ | $C_{20}H_{28}O_3$ | 3 | 0.6 | Nf | n.a. | | | |
| 36 | 235.1341 | 7.25 | $[M+H]^+$ | $C_{14}H_{18}O_3$ | 3 | 3 | 207.1382, 203.1069, 193.1229, 191.1428, 189.1278, 185.0968, 179.1072, 175.1121, 161.0964, 159.1173, 157.1014, 147.1168, 144.0936, 142.0781, 139.0398, 133.1015, 129.0697, 119.0860, 105.0702 | n.a. | | | |
| 37 | 567.1875 | 7.32 | $[M+H]^+$ | $C_{30}H_{30}O_{11}$ | 3 | 1.6 | 531.1674, 513.1566, 495.1455, 485.1612, 467.1511, 391.1179, 389.1031, 373.1081, 371.0923, 363.1235, 275.0925, 273.0772, 267.0664, 259.0970, 257.0821, 241.0869, 229.0868, 217.0868, 167.0346, 153.0550, 151.0397, 123.0446 | n.a. | | | |
| 38 | 319.2287 | 7.51 | $[M+H]^+$ | $C_{20}H_{30}O_3$ | 3 | 4.4 | Nf | n.a. | | | |
| 39 | 317.2125 | 7.7 | $[M+H]^+$ | $C_{20}H_{28}O_3$ | 3 | 2.5 | 299.2018, 281.1901, 271.2071, 253.1964, 243.1376, 201.1267, 173.1336, 159.1179, 145.1019, 139.0778, 133.1020, 119.0862, 95.0862 | n.a. | | | |
| 40 | 317.2125 | 7.88 | $[M+H]^+$ | $C_{20}H_{28}O_3$ | 3 | 2.5 | Nf | n.a. | | | |
| 41 | 417.2249 | 8.16 | $[M+Na]^+$ | $C_{22}H_{34}O_6$ | 3 | -1 | Nf | Aphidicolin A32 | Diterpenoid | *Botryotinia fuckeliana* (fungus) | [5] |
| 42 | 331.1914 | 8.16 | $[M+H]^+$ | $C_{20}H_{26}O_4$ | 3 | 1.5 | 285.1862, 271.1705, 255.1378, 253.1606, 246.1281, 243.1753, 227.1440, 191.0714, 185.0970, 175.0758, 163.0769 | Hawaiinolide A | Diterpenoid | *Paraconiothyrium hawaiiense* (fungus) | [9] |
| 43 | 498.3797 | 8.35 | $[M+H]^+$ | $C_{28}H_{51}NO_6$* | 4 | 0.4 | 480.3681, 236.1501, 162.1126, 144.1023 | n.a. | | | |
| 44 | 498.3795 | 8.54 | $[M+H]^+$ | $C_{28}H_{51}NO_6$* | 4 | 0 | 480.3691, 436.3773, 236.1502, 162.1126, 144.1023 | n.a. | | | |
| 45 | 287.2377 | 8.7 | $[M+H]^+$ | $C_{20}H_{30}O$ | 3 | 0.7 | 269.2265, 241.1960, 227.1789, 215.1799, 213.1643, 199.1494, 185.1323, 175.1504, | Conidiogenone B | Diterpenoid | *Penicillium* sp. (fungus) | [13] |

| No. | *m/z* value | $R_t$ (min) | Adduct | Putative molecular formula | IC | ppm | Fragmentation pattern | Putative identification | Chemical family | Biological origin | Ref |
|---|---|---|---|---|---|---|---|---|---|---|---|
| | | | | | | | 173.1328, 171.1169, 161.1331, 159.1170, 157.1014, 151.1124, 147.1160, 145.1029, 133.1035, 131.0861, 119.0863 | | | | |
| 46 | 401.2299 | 8.78 | $[M+Na]^+$ | $C_{22}H_{34}O_5$ | 3 | -1.2 | Nf | a: aphidicolin A23, b: aphidicolin A50 | Diterpenoid | *Botryotinia fuckeliana* (fungus) | [5] |
| 47 | 347.222 | 8.78 | $[M+H]^+$ | $C_{21}H_{30}O_4$ | 3 | -0.6 | 329.2059, 269.1911, 251.1773 225.1639,159.1180, 145.1011 | Aspergillodiol | Hydropyrano-indeno derivative | *Aspergillus versicolor* (fungus) | [14] |
| 48 | 500.3948 | 9.06 | $[M+H]^+$ | $C_{28}H_{53}NO_6$* | 4 | -0.6 | 482.3836, 438.3966, 236.1499, 162.1135, 144.1021 | n.a. | | | |
| 49 | 551.1915 | 9.17 | $[M+H]^+$ | $C_{30}H_{30}O_{10}$ | 4 | -0.4 | 497.1592, 391.1183, 373.1078, 267.0645, 259.0966, 257.0809, 241.0867, 229.0860, 217.0868, 153.0541, 151.0395 | n.a. | | | |
| 50 | 301.2168 | 9.25 | $[M+H]^+$ | $C_{20}H_{28}O_2$ | 3 | 0 | 283.2092, 255.2111, 227.1792, 185.1351, 175.1486, 173.1334, 171.1149, 159.1779, 157.1020, 147.1188, 145.1016, 133.1026, 121.1039, 119.0863, 107.0864, 105.0708, 95.0862 | Harziandione | Diterpenoid | *Trichoderma atroviride* (fungus) | [15] |
| 51 | 303.2333 | 9.33 | $[M+H]^+$ | $C_{20}H_{30}O_2$ | 3 | 3 | 285.2242, 177.1627, 175.1490 151.9207, 149.0956, 147.1167, 139.0779, 123.1156, 121.1021, 109.1024, 107.0853, 105.0681, 95.0868, 93.0696, 81.0703, 79.0540 | Botrysphin B | Diterpenoid | *Botryosphaeria laricina* (fungus) | [16] |

**Table S7. Putative annotation of metabolites detected in the crude extract of *Pseudogymnoascus destructans* strain CHT56 cultivated on CAG medium.** Each detected compound is given with the experimentally determined *m/z* value and the predicted putative molecular formula. Putative identifications were based on the accurate mass, predicted putative molecular formulae, the retention time ($R_t$ in min), the fragmentation pattern and biological origin. *Only putative molecular formula with best ppm shown (more than 1 molecular formula possible). $^{\Delta}$Different isomers with same *m/z* value and molecular formula, which cannot be differentiated based on MS/MS data. IC: Identification confidence level after Sumner et al. 2007 [3]. Nf: No fragmentation pattern detected or fragmentation below noise threshold of $5e^1$. n.a. = putatively novel compound (known NPs do not match). Ref = reference.

| No. | *m/z* value | $R_t$ (min) | Adduct | Putative molecular formula | IC | ppm | Fragmentation pattern | Putative identification | Chemical family | Biological origin | Ref |
|---|---|---|---|---|---|---|---|---|---|---|---|
| 52 | 413.1804 | 3.91 | $[M-H_2O]^+$ | $C_{17}H_{22}N_{10}O_4$* | 4 | 1.5 | 249.118, 245.1402, 221.1146, 129.0537 | n.a. | | | |
| 53 | 267.1223 | 4.84 | $[M+H]^+$ | $C_{14}H_{18}O_5$ | 4 | -3.4 | 249.1100, 221.1175, 151.0368, 123.0437, 99.0797, 71.0863 | n.a. | | | |
| 54 | 291.1222 | 4.96 | $[M+H]^+$ | $C_{16}H_{18}O_5$ | 4 | -3.4 | 273.1115, 245.1171, 193.0531, 99.0804 | n.a. | | | |
| 55 | 281.1017 | 5.11 | $[M+H]^+$ | $C_{14}H_{16}O_6$ | 3 | -2.8 | 221.0442, 211.0230, 207.1018, 191.0332, 165.0531 | Corynechromone E or F | Chromone derivative | *Corynespora cassiicola* (fungus) | [17] |
| 56 | 253.1067 | 5.14 | $[M+H]^+$ | $C_{13}H_{16}O_5$ | 3 | -3.6 | 235.0970, 207.1010, 193.0438, 183.0279, 165.0173, 139.0372, 137.0223 | Acremostrictin | Sesquiterpenoid | *Acremonium strictum* (fungus) | [18] |
| 57 | 265.1065 | 5.34 | $[M+H]^+$ | $C_{14}H_{16}O_5$ | 4 | -4.1 | 247.0953, 237.1100, 219.1009, 209.0434, 201.0900, 177.0532, 165.0532 | n.a. | | | |
| 58 | 265.1069 | 5.48 | $[M+H]^+$ | $C_{14}H_{16}O_5$ | 4 | -2.6 | 247.0973, 219.1008, 209.0424, 201.0903, 177.0530, 165.0533 | n.a. | | | |
| 59 | 291.1227 | 5.59 | $[M+H]^+$ | $C_{16}H_{18}O_5$ | 4 | -1.7 | 273.1115, 245.1171, 193.0531, 99.0804 | n.a. | | | |
| 60 | 249.1116 | 5.82 | $[M+H]^+$ | $C_{14}H_{16}O_4$ | 3 | -4.4 | 221.1166 | Phialofurone | Benzofuran derivative | *Phialocephala* sp. (fungus) | [19] |
| 61 | 295.1172 | 6.05 | $[M+H]^+$ | $C_{15}H_{18}O_6$ | 3 | -3.4 | 267.1231, 251.1295, 235.0968, 221.0431, 217.0852, 207.1008, 197.0437, 165.0538 | 3,4-dihydro-6-methoxy-8-hydroxy-3,4,5-trimethyl-isocoumarin-7-carboxylic acid methyl ester | Isocoumarin derivative | Unknown fungus | [20] |
| 62 | 211.0596 | 6.28 | $[M+H]^+$ | $C_{10}H_{10}O_5$ | 3 | -4.7 | 179.0332, 151.0377, 128.9502 | Hypoxyphenone | Benzoic acid derivative | *Hypoxylon* sp. (fungus) | [21] |
| 63 | 525.3033 | 6.46 | $[M+H]^+$ | $C_{23}H_{44}N_2O_{11}$ | 4 | 1.9 | Nf | n.a. | | | |
| 64 | 467.2999 | 6.46 | $[M+H]^+$ | $C_{26}H_{42}O_7$ | 4 | -2.1 | 177.1277, 175.1137, 149.1330, 135.1166, 133.1003, 121.0978, 113.0946, 109.1005, 95.0862 | n.a. | | | |
| 65 | 529.2076 | 6.61 | $[M+H]^+$ | $C_{28}H_{32}O_{10}$ | 3 | 0.4 | Nf | Aspermeroterpene A | Meroterpenoid | *Aspergillus terreus* (fungus) | [22] |
| 66 | 357.2034 | 6.75 | $[M+H]^+$ | $C_{18}H_{24}N_6O_2$* | 4 | -1.4 | Nf | n.a. | | | |

| No. | *m/z* value | $R_t$ (min) | Adduct | Putative molecular formula | IC | ppm | Fragmentation pattern | Putative identification | Chemical family | Biological origin | Ref |
|---|---|---|---|---|---|---|---|---|---|---|---|
| 67 | 529.2073 | 6.87 | $[M+H]^+$ | $C_{28}H_{32}O_{10}$ | 3 | -0.2 | Nf | Aspermeroterpene B | Meroterpenoid | *Aspergillus terreus* (fungus) | [22] |
| 68 | 789.2764 | 7.06-7.18 | $[M+H]^+$ | $C_{42}H_{44}O_{15}$ | 4 | 0.8 | Nf | n.a. | | | |
| 69 | 1053.3766 | 7.09 | $[M+H]^+$ | $C_{52}H_{36}N_{28}$* | 4 | 0.9 | 971.3932, 735.2479, 435.2361, 247.0930 | n.a. | | | |
| 70 | 317.2102 | 7.41, $7.9^{\Delta}$ | $[M+H]^+$ | $C_{20}H_{28}O_3$ | 3 | -4.7 | 299.2006, 281.1910, 263.1822, 237.1586, 237.1611, 197.1319, 151.1088, 137.0890 | (9ξ,13α)-6,9-dihydroxypimara-5,8(14),15-trien-7-one | Pimarane diterpenoid | *Epicoccum* sp. (fungus) | [23] |
| 71 | 789.2755 | 7.41 | $[M+H]^+$ | $C_{42}H_{44}O_{15}$ | 4 | -0.4 | 555.1884, 408.3155 | n.a. | | | |
| 72 | 393.2422 | 8.2 | $[M+H]^+$ | $C_{26}H_{32}O_3$ | 4 | -2 | 315.1731, 293.2056, 273.1625, 249.1645, 243.1741, 235.1513, 225.1621, 173.1361, 171.1132, 159.1169, 133.0636, 105.0683 | n.a. | | | |
| 73 | 393.2424 | 9.07 | $[M+H]^+$ | $C_{26}H_{32}O_3$ | 4 | -1.5 | 375.2322, 243.1759, 225.1634, 223.1468, 195.1156, 183.1155, 169.1017, 159.1152, 157.0998, 145.1002. 133.0650, 131.0474 | n.a. | | | |
| 74 | 263.2363 | 10.47 | $[M+H]^+$ | $C_{18}H_{30}O$ | 4 | -4.6 | 245.2263, 175.1476, 149.1320, 133.1000, 123.1158, 121.1015, 109.0997, 81.0693 | n.a. | | | |
| 75 | 337.1665 | 10.47 | $[M+H]^+$ | $C_{14}H_{21}N_8Cl$ | 4 | 2.7 | 185.0091 | n.a. | | | |

**Table S8. Putative annotation of metabolites detected in the crude extract of *Penicillium* sp. strain CKT35 cultivated on PDA medium.** Each detected compound is given with the experimentally determined *m/z* value and the predicted putative molecular formula. Putative identifications were based on the accurate mass, predicted putative molecular formulae, the retention time ($R_t$ in min), the fragmentation pattern and biological origin. *Only putative molecular formula with best ppm shown (more than 1 molecular formula possible). $^{\Delta}$Different isomers with same *m/z* value and molecular formula, which cannot be differentiated based on MS/MS data. IC: Identification confidence level after Sumner et al. 2007 [3]. Nf: No fragmentation pattern detected or fragmentation below noise threshold of $5e^1$. n.a. = putatively novel compound (known NPs do not match). Ref = reference.

| No. | *m/z* value | $R_t$ (min) | Adduct | Putative molecular formula | IC | ppm | Fragmentation pattern | Putative identification | Chemical family | Biological origin | Ref |
|---|---|---|---|---|---|---|---|---|---|---|---|
| 76 | 207.0302 | 2.14, 2.64, 4.86$^{\Delta}$ | $[M+H]^+$ | $C_{10}H_6O_5$ | 3 | 4.3 | 179.0345, 165.0189, 161.0234, 137.0238 | Flaviolin | Naphthoquinone derivative | Several fungal taxa, e.g. *Aspergillus niger* | [24] |
| 77 | 209.0454 | 2.33 | $[M+H]^+$ | $C_{10}H_8O_5$ | 2 | 1.9 | 191.0349, 181.0501, 167.0342, 163.0397, 147.0449, 135.0445 | Penibenzone C | Phthalide derivative | *Penicillium purpurogenum* (fungus) | [25] |
| 78 | 195.0299 | 2.53 | $[M+H]^+$ | $C_9H_6O_5$ | 4 | 3.1 | 167.0345, 163.0031, 135.0089, 119.0136 | n.a. | | | |
| 79 | 193.0512 | 3.07 | $[M+H]^+$ | $C_{10}H_8O_4$ | 3 | 5.7 | 175.0393 | Penicifuran C | Benzofuran | *Penicillium* sp. (fungus) | [26] |
| 80 | 209.0459 | 3.23 | $[M+H]^+$ | $C_{10}H_8O_5$ | 2 | 4.3 | 163.0398 | Acetophthalidin | Phthalide derivative | *Penicillium* sp. (fungus) | [27] |
| 81 | 293.1273 | 3.77 | $[M+Na]^+$ | $C_{16}H_{18}N_2O_2$ | 3 | 2.4 | 214.0741 | Quinolactacin A | Quinolone alkaloid | *Penicillium* sp. (fungus) | [28] |
| 82 | 193.0509 | 4.31 | $[M+H]^+$ | $C_{10}H_8O_4$ | 3 | 4.1 | 175.0393, 149.0592, 121.0658 | Penicifuran D | Benzofuran | *Penicillium* sp. (fungus) | [26] |
| 83 | 337.1292 | 4.59 | $[M+H]^+$ | $C_{17}H_{20}O_7$ | 2 | 1.5 | Nf | 4'-hydroxy-mycophenolic acid | Meroterpenoid | *Penicillium* sp. (fungus) | [29] |
| 84 | 319.1176 | 5.1 | $[M+H]^+$ | $C_{17}H_{18}O_6$ | 2 | -1.9 | Nf | 4-hydroxy-6-methoxy-γ,7-dimethyl-3-oxo-5-phthalansorbic acid | Meroterpenoid | *Penicillium rugulosum* (fungus) | [30] |
| 85 | 321.1345 | 5.76 | $[M+H]^+$ | $C_{17}H_{20}O_6$ | 2 | 2.2 | 303.1235, 275.12987, 207.0662, 195.0657, 159.0446) | Mycophenolic acid | Meroterpenoid | *Penicillium* sp. (fungus) | [29] |
| 86 | 441.2284 | 6.47 | $[M+H]^+$ | $C_{26}H_{32}O_6$ | 3 | 1.6 | 363.1965, 211.1483, 173.1311, 171.1171, 151.0382 | Tropolactone C | Meroterpenoid | *Aspergillus* sp. (fungus) | [31] |
| 87 | 501.2503 | 6.47 | $[M+H]^+$ | $C_{28}H_{36}O_8$ | 3 | 0.2 | 409.2018, 391.1942, 381.2094, 363.1965, 335.2033, 221.1305, 217.1243, 213.1653, 211.1483, 203.1084, 187.1500, 183.1351, 177.0918, 171.1171, 157.1011, 151.0382 | Citreohybridonol | Meroterpenoid | *Penicillium atrovenetum* (fungus) | [32] |
| 88 | 471.2703 | 6.55 | $[M+H]^+$ | $C_{23}H_{38}N_2O_8$* | 4 | -0.6 | 439.2438, 261.1821, 232.0702 | n.a. | | | |
| 89 | 523.2224 | 6.91 | $[M+H]^+$ | $C_{32}H_{30}N_2O_5$ | 2 | -1.7 | 256.1335, 238.1228, 134.0960, 122.0595, 117.0701, 105.0338 | Asperphenamate B | Phenylalanine derivative | *Penicillium* spp. (fungus) | [33] |

| No. | *m/z* value | $R_t$ (min) | Adduct | Putative molecular formula | IC | ppm | Fragmentation pattern | Putative identification | Chemical family | Biological origin | Ref |
|---|---|---|---|---|---|---|---|---|---|---|---|
| 90 | 335.1501 | 7.14 | $[M+H]^+$ | $C_{18}H_{22}O_6$ | 2 | 1.8 | 303.1234, 285.1124, 275.1286, 207.0660, 195.0656, 159.0443 | Mycophenolic acid methyl ester | Meroterpenoid | *Penicillium* sp. | [34] |
| 91 | 629.0908 | 7.29 | $[M+H]^+$ | $C_{28}H_{16}N_6O_{12}$* | 4 | 0.6 | 593.0682, 552.0537, 534.0625, 335.0278, 253.0173 | n.a. | | | |
| 92 | 487.27 | 7.4 | $[M+H]^+$ | $C_{28}H_{38}O_7$ | 2 | 0.8 | 377.2110, 349.2166, 243.1750, 225.1644, 215.1801, 199.1489, 185.1326, 175.1487, 159.1171, 157.1014, 151.0391, 145.1013 | Andrastin A | Meroterpenoid | *Penicillium* sp. (fungus) | [35] |
| 93 | 473.254 | 7.52 | $[M+H]^+$ | $C_{27}H_{36}O_7$ | 3 | 0.2 | MS2 (many ions, only >200 noted): 353.2104, **343.2300**, **311.1978**, 293.1867, 283.2126, **237.0764**, 217.1185, 209.1330, 197.0464, 191.0735, 187.1497, 185.1344, 183.1164, 177.0534, 173.1350, **171.1151**, **159.1188**, 157.0966, 145.1013, 131.0849 | Citreohybridone C | Meroterpenoid | *Penicillium citreo-viride* (fungus) | [36] |
| 94 | 568.2222 | 7.76 | $[M+Na]^+$ | $C_{35}H_{27}N_7$* | 4 | -0.7 | 331.1059, 260.1011, 238.1237, 181.0751, 122.0594, 117.0715, 105.0346) | n.a. | | | |
| 95 | 581.3674 | 7.76 | $[M+H]^+$ | $C_{29}H_{44}N_{10}O_3$* | 4 | -0.3 | 367.2092, 237.1466 | n.a. | | | |
| 96 | 507.2307 | 8.03 | $[M+H]^+$ | $C_{33}H_{26}N_6$ | 2 | 2 | 256.1349, 238.1242, 224.1082, 122.0608, 117.0708, 105.0345 | Asperphenamate | Phenylalanine derivative | *Penicillium* spp. (fungus) | [33] |
| 97 | 376.2852 | 11.62 | $[M+H]^+$ | $C_{23}H_{37}NO_3$ | 4 | 0 | 138.0554, 120.0449, 92.0501 | n.a. | | | |

**Table S9. Putative annotation of metabolites detected in the crude extracts of *Boeremia exigua* strain CKT91 cultivated on CAG and PDA media.** Each detected compound is given with the experimentally determined *m/z* value and the predicted putative molecular formula. Putative identifications were based on the accurate mass, predicted putative molecular formulae, the retention time ($R_t$ in min), the fragmentation pattern and biological origin. $^{\Delta}$Different isomers with same *m/z* value and molecular formula, which cannot be differentiated based on MS/MS data. IC: Identification confidence level after Sumner et al. 2007 [3]. Nf: No fragmentation pattern detected or fragmentation below noise threshold of $5e^1$. n.a. = putatively novel compound (known NPs do not match). Ref = reference.

| No. | *m/z* value | $R_t$ (min) | Adduct | Putative molecular formula | IC | ppm | Fragmentation pattern | Putative identification | Chemical family | Biological origin | Medium | Ref |
|---|---|---|---|---|---|---|---|---|---|---|---|---|
| 98 | 303.1199 | 2.67 | $[M+H]^+$ | $C_{12}H_{18}N_2O_7$ | 4 | 2.3 | Nf | n.a. | | | PDA | |
| 99 | 600.2635 | 5.78 | $[M+H]^+$ | $C_{24}H_{37}N_7O_{11}$ | 4 | 1 | Nf | n.a. | | | CAG, PDA | |
| 100 | 480.2745 | 5.92 | $[M+H]^+$ | $C_{29}H_{37}NO_5$ | 2 | -1 | 462.2639, 444.2558, 426.2426, 416.2584, 398.2487, 378.2067, 278.1534, 264.1393, 252.1384, 240.1393, 209.1315, 187.1104, 172.0756, 159.1155 145.1000, 120.0790, 105.0688, 91.0529 | Cytochalasin B2 | Cytochalasan | *Phoma* sp. (fungus) | CAG, PDA | [37] |
| 101 | 464.2794 | 6.12, 7.6, 8.39, 8.5, 9.45, 9.74$^{\Delta}$ | $[M+H]^+$ | $C_{29}H_{37}NO_4$ | 3 | -1.5 | 447.2738, 446.2693, 429.2610, 428.2582, 418.2767, 410.2479, 400.2635, 281.1872 , 280.1693, 268.1689, 266.1530, 264.1843, 263.1788, 254.1529, 252.1370, 172.0747, 161.0949, 157.1004, 147.1151, 145.1000, 143.0845, 133.0997, 131.0843, 120.0798, 119.0840, 105.0687, 91.0533 | Deoxaphomin | Cytochalasan | *Phoma exigua* (fungus) | CAG, PDA | [38] |
| 102 | 480.2748 | 6.3 | $[M+H]^+$ | $C_{29}H_{37}NO_5$ | 2 | -0.4 | 462.2648, 444.2540, 416.2583, 282.1481, 264.1377, 120.0796 | Cytochalasin B | Cytochalasan | *Phoma exigua* (fungus) | CAG, PDA | [38] |
| 103 | 480.2743 | 6.41 | $[M+H]^+$ | $C_{29}H_{37}NO_5$ | 2 | -1.5 | 462.2609, 444.2491, 240.1376, 212.1434, 195.1154, 120.0796 | Cytochalasin B6 | Cytochalasan | *Phoma* sp. (fungus) | CAG, PDA | [39] |
| 104 | 480.2746 | 7 | $[M+H]^+$ | $C_{29}H_{37}NO_5$ | 2 | -0.8 | 462.2639, 444.2563, 398.2440, 278.1548, 264.1373, 240.1374, 226.1206, 200.1076, 186.1276, 159.1150, 145.1015, 133.1019, 120.0789, 91.0525 | Cytochalasin B4 | Cytochalasan | *Phoma* sp. (fungus) | CAG, PDA | [39] |
| 105 | 510.2865 | 7.09 | $[M+H]^+$ | $C_{30}H_{39}NO_6$ | 2 | 1.8 | 478.2596, 460.2494, 442.2400, 414.2450, 406.2306, 396.2238, 388.2231, 376.1968, 278.1477, 264.1334, 240.1387, 209.1319, 186.0871, 149.0972, 131.0830, 119.0824 | Cytochalasin Z11 | Cytochalasan | *Endothia gyrosa* (fungus) | PDA | [40] |
| 106 | 478.2589 | 7.24 | $[M+H]^+$ | $C_{29}H_{35}NO_5$ | 2 | -0.8 | 460.2487, 442.2378, 432.2536, 414.2420, 278.1541, 266.1538, 264.1380, 252.1379, 250.1227, 240.1375, 198.0917, 185.0943, 172.0744, 157.1004, 145.1001, 143.0848, 133.1000, 131.0834, 120.0796, 119.0846, 105.0692, 91.0534 | Cytochalasin B3 | Cytochalasan | *Phoma* sp. (fungus) | CAG, PDA | [39] |

| No. | *m/z* value | $R_t$ (min) | Adduct | Putative molecular formula | IC | ppm | Fragmentation pattern | Putative identification | Chemical family | Biological origin | Medium | Ref |
|---|---|---|---|---|---|---|---|---|---|---|---|---|
| 107 | 448.2843 | 8.7, 10.98$^{\Delta}$ | $[M+H]^+$ | $C_{29}H_{37}NO_3$ | 3 | -2 | 449.2923, 430.2736, 229.1962, 215.1794, 200.0701, 172.0741, 121.1012, 109.1021, 107.0821, 95.0857 | Deoxaphomin C | Cytochalasan | *Phoma* sp. (fungus) | CAG, PDA | [37] |
| 108 | 355.2838 | 9.78 | $[M+H]^+$ | $C_{21}H_{38}O_4$ | 4 | -2.8 | 337.2741, 263.2357, 245.2267, 213.9730, 175.1456, 163.1457, 149.1315, 135.1169, 123.1169, 121.0997, 116.0520, 109.1000, 97.0990, 81.0698 | n.a. | | | PDA | |
| 109 | 427.3204 | 10.2 | $[M+H]^+$ | $C_{28}H_{42}O_3$ | 2 | -1.9 | 409.3159, 391.3037, 283.1731, 125.1308, 69.0689 | Dankasterone B | Ergosterol | *Gymnascella dankaliensis* (fungus) | PDA | [41] |
| 110 | 432.2898 | 10.5 | $[M+H]^+$ | $C_{29}H_{37}NO_2$ | 2 | -1.2 | 241.1952, 217.1948, 200.0696, 172.0747, 147.1157, 121.1003, 109.0999 | Proxiphomin | Cytochalasan | *Phoma* sp. (fungus) | CAG, PDA | [39] |
| 111 | 395.3304 | 11.82 | $[M+H]^+$ | $C_{28}H_{42}O$ | 4 | -2.5 | 311.2373, 307.2408, 293.2247, 251.1765, 211.1485, 199.1459, 159.1105, 109.1014, 83.0846, 69.0697 | n.a. | | | PDA | |

**Table S10. Putative annotation of metabolites detected in the crude extracts of *Streptomyces* sp. strain CKT43 cultivated on GYM and MB media.** Each detected compound is given with the experimentally determined *m/z* value and the predicted putative molecular formula. Putative identifications were based on the accurate mass, predicted putative molecular formulae, the retention time ($R_t$ in min), the fragmentation pattern and biological origin. *Only putative molecular formula with best ppm shown (more than 1 molecular formula possible). IC: Identification confidence level after Sumner et al. 2007 [3]. Nf: No fragmentation pattern detected or fragmentation below noise threshold of $5e^1$. n.a. = putatively novel compound (known NPs do not match). Ref = reference.

| No. | *m/z* value | $R_t$ (min) | Adduct | Putative molecular formula | IC | ppm | Fragmentation pattern | Putative identification | Chemical family | Biological origin | Medium | Ref |
|---|---|---|---|---|---|---|---|---|---|---|---|---|
| 112 | 160.0767 | 1.45 | $[M+H]^+$ | $C_{10}H_9NO$ | 4 | 3.1 | 132.0818 | n.a. | | | MB | |
| 113 | 265.1423 | 3.72 | $[M+H]^+$ | $C_{11}H_{16}N_6O_2$ | 4 | 3.8 | Nf | n.a. | | | MB, GYM | |
| 114 | 263.1251 | 4.01 | $[M+H]^+$ | $C_{11}H_{14}N_6O_2$ | 4 | -1.9 | Nf | n.a. | | | MB, GYM | |
| 115 | 263.1249 | 4.12 | $[M+H]^+$ | $C_{11}H_{14}N_6O_2$ | 4 | -2.7 | Nf | n.a. | | | MB, GYM | |
| 116 | 235.1317 | 4.51 | $[M+H]^+$ | $C_{10}H_{14}N_6O$ | 4 | 4.3 | 195.1388, 177.1278 | n.a. | | | MB | |
| 117 | 235.1319 | 4.85 | $[M+H]^+$ | $C_{10}H_{14}N_6O$ | 4 | 5.1 | 195.1378, 177.1292 | n.a. | | | MB | |
| 118 | 743.4458 | 4.76 | $[M+H]^+$ | $C_{37}H_{58}N_8O_8$* | 4 | 0.3 | 341.2543, 298.2151, 284.1961, 276.1321, 157.0986, 86.0920 | n.a. | | | GYM | |
| 119 | 233.1158 | 5.08 | $[M+H]^+$ | $C_{10}H_{12}N_6O$ | 4 | 2.6 | 157.9664 | n.a. | | | MB | |
| 120 | 249.1475 | 5.19 | $[M+H]^+$ | $C_{11}H_{16}N_6O$ | 4 | 4.4 | 209.1536, 191.1433, 163.1482 | n.a. | | | MB, GYM | |
| 121 | 249.1472 | 5.3 | $[M+H]^+$ | $C_{11}H_{16}N_6O$ | 4 | 3.2 | 209.1569, 191.1430 | n.a. | | | GYM | |
| 122 | 221.191 | 5.38 | $[M+H]^+$ | $C_{15}H_{24}O$ | 4 | 2.3 | 203.1797, 147.1173, 141.9590, 97.9693 | n.a. | | | MB | |
| 123 | 225.1501 | 5.52 | $[M+H]^+$ | $C_{13}H_{20}O_3$ | 2 | 4.4 | 207.1390, 197.1548, 189.1286, 179.1438, 165.1284, 161.1334, 151.0760, 147.1176, 147.0811, 137.0605. 133.0656, 125.0604, 119.0864, 107.0862, 107.0500, 105.0706 | MKN-003A | Butenolide | *Streptomyces* sp. (bacterium) | MB, GYM | [42] |
| 124 | 227.1656 | 5.75 | $[M+H]^+$ | $C_{13}H_{22}O_3$ | 3 | 4 | Nf | MKN-003C | Butenolide | *Streptomyces* sp. (bacterium) | MB | [42] |
| 125 | 955.5981 | 5.75 | $[M+H]^+$ | $C_{48}H_{78}N_{10}O_{10}$ Or $C_{46}H_{66}N_{24}$* | 4 | 0.3 | 626.3671, 539.3618, 511.3620, 470.2799, 468.3213, 428.2272, 412.2930, 400.2314, 371.2073, 369.2507, 357.1931, 355.2360, 341.2551, 324.2289, 313.2219, 308.2020, 300.1698, 298.2097, 284.1980, 272.1780, 270.1830, 259.1266, 256.1674, 253.1931, 242.1522, 239.1759, 231.1698, 230.1304, 213.1609, 211.1805, 211.1448, 199.447, 197.1678, 187.0869, 185.1291, 171.1505, | n.a. | | | MB | |

| No. | *m/z* value | $R_t$ (min) | Adduct | Putative molecular formula | IC | ppm | Fragmentation pattern | Putative identification | Chemical family | Biological origin | Medium | Ref |
|---|---|---|---|---|---|---|---|---|---|---|---|---|
| | | | | | | | 171.1134, 159.0932, 157.0972, 143.1185, 86.0971 | | | | | |
| 126 | 898.6118 | 6.02 | $[M+H]^+$ | $C_{47}H_{79}N_9O_8$ | 2 | -1.3 | 785.5298, 615.4265, 445.2813, 412.2908, 397.2446, 332.1974, 298.2133, 284.1965, 261.1606, 253.1929, 233.1674, 228.1719, 213.1601, 199.1820, 197.1653, 185.1291, 157.1363, 155.1204, 129.1028, 86.0971, 84.0820 | Surugamide B | Cyclic octapeptide | *Streptomyces* sp. (bacterium) | MB, GYM | [43] |
| 127 | 898.6117 | 6.1 | $[M+H]^+$ | $C_{47}H_{79}N_9O_8$ | 2 | -1.4 | 686.4632, 615.4197, 554.3472, 397.2428, 369.2845, 298.2137, 284.1965, 261.1610, 233.1666, 227.1764, 213.1611, 197.1669, 185.1291, 171.1122, 129.1036, 86.0971, 84.0809 | Surugamide C | Cyclic octapeptide | *Streptomyces* sp. (bacterium) | MB, GYM | [43] |
| 128 | 898.6121 | 6.18 | $[M+H]^+$ | $C_{47}H_{79}N_9O_8$ | 2 | -1 | 785.5309, 601.4060, 502.3380, 445.2841, 431.2659, 397.2429, 374.2444, 369.2854, 360.2286, 332.1981, 261.1602, 253.1920, 242.1852, 239.1767, 233.1660, 199.1831,157.1340, 129.1031, 120.0808, 86.0979, 84.0811 | Surugamide D | Cyclic octapeptide | *Streptomyces* sp. (bacterium) | MB, GYM | [43] |
| 129 | 912.6266 | 6.29 | $[M+H]^+$ | $C_{48}H_{81}N_9O_8$ | 2 | -2.2 | 445.2818, 374.2439, 298.2127, 261.1596, 129.1012 | Surugamide A | Cyclic octapeptide | *Streptomyces* sp. (bacterium) | MB, GYM | [43] |
| 130 | 448.3065 | 6.61 | $[M+H]^+$ | $C_{26}H_{41}NO_5$* | 4 | 0.4 | 430.2956, 412.2844, 355.2642, 337.2538, 319.2439, 213.1642 | n.a. | | | MB | |
| 131 | 389.2693 | 6.79 | $[M+H]^+$ | $C_{24}H_{36}O_4$ | 4 | 0.3 | 371.2574, 353.2473, 335.2369, 325.2511, 317.2269, 239.1788, 229.1589, 177.1292, 109.0649, 97.0651 | n.a. | | | MB | |
| 132 | 1054.629 | 6.97 | $[M+H]^+$ | $C_{51}H_{87}N_7O_{16}$* | 4 | 0.2 | Nf | n.a. | | | MB | |
| 133 | 669.4541 | 7.43 | $[M+H]^+$ | $C_{32}H_{64}N_2O_{12}$* | 4 | 0.4 | 339.2649, 313.1871, 297.1458, 240.0987, 228.2325, 203.1395, 186.1132 | n.a. | | | MB | |
| 134 | 600.1503 | 7.51 | $[M+H]^+$ | $C_{30}H_{13}N_{15}O$* | 4 | -0.3 | 550.1147, 540.1298, 522.1205 | n.a. | | | GYM | |
| 135 | 683.4688 | 7.84 | $[M+H]^+$ | $C_{31}H_{54}N_{16}O_2$ Or $C_{33}H_{66}N_2O_{12}$* | 4 | -0.9 | 353.2797, 313.1877, 297.1457, 242.2488, 240.0988, 203.1399, 186.1134 | n.a. | | | MB | |
| 136 | 683.4688 | 7.9 | $[M+H]^+$ | $C_{31}H_{54}N_{16}O_2$ Or $C_{33}H_{66}N_2O_{12}$* | 4 | -0.9 | 353.2797, 313.1878, 297.1456, 242.2488, 240.0988, 203.1399, 186.1132 | n.a. | | | MB | |
| 137 | 697.4856 | 8.33 | $[M+H]^+$ | $C_{34}H_{68}N_2O_{12}$* | 4 | 0.7 | 313.1876, 297.1454, 256.2643, 240.0979, 203.1400, 186.1130 | n.a. | | | MB | |
| 138 | 315.2528 | 8.38 | $[M+H]^+$ | $C_{18}H_{34}O_4$ | 4 | -2.2 | 97.1003, 83.0846, 75.0433 | n.a. | | | MB, GYM | |
| 139 | 315.2523 | 8.46 | $[M+H]^+$ | $C_{18}H_{34}O_4$ | 4 | -3.8 | 111.1182, 97.1002, 83.0843, 75.0430 | n.a. | | | MB, GYM | |

| No. | *m/z* value | $R_t$ (min) | Adduct | Putative molecular formula | IC | ppm | Fragmentation pattern | Putative identification | Chemical family | Biological origin | Medium | Ref |
|---|---|---|---|---|---|---|---|---|---|---|---|---|
| 140 | 205.1964 | 8.71 | $[M+H]^+$ | $C_{15}H_{24}$ | 4 | 3.9 | 149.1333, 135.1174, 123.1174, 121.1017, 109.1018, 107.0859, 95.0865 | n.a. | | | MB, GYM | |
| 141 | 219.1741 | 9.03 | $[M+H]^+$ | $C_{15}H_{22}O$ | 3 | -3.7 | 177.1264, 163.1107, 149.0943 | Anaephene A | Alkylphenol derivative | *Hormoscilla* sp. (bacterium) | MB, GYM | [44] |
| 142 | 357.2634 | 9.32 | $[M+H]^+$ | $C_{20}H_{36}O_5$ | 4 | -2 | 117.0534, 83.0846 | n.a. | | | GYM | |
| 143 | 785.3582 | 9.32 | $[M+H]^+$ | $C_{33}H_{36}N_{24}O_*$ | 4 | 0 | Nf | n.a. | | | MB, GYM | |
| 144 | 799.3724 | 9.67 | $[M+H]^+$ | $C_{33}H_{42}N_{20}O_5$ Or $C_{35}H_{54}N_6O_{15*}$ | 4 | -0.1 | 711.3188 | n.a. | | | GYM | |
| 145 | 507.2712 | 9.79 | $[M+H]^+$ | $C_{26}H_{38}N_2O_8$ | 2 | 1.2 | 237.0884, 136.0401 | Deformylated antimycin A2a | Macrolide | *Streptomyces* sp. (bacterium) | MB | [45] |
| 146 | 521.2866 | 10.2 | $[M+H]^+$ | $C_{27}H_{40}N_2O_8$ | 2 | 0.6 | 237.0881, 136.0396 | Deformylated antimycin A1a | Macrolide | *Streptomyces* sp. (bacterium) | MB, GYM | [45] |
| 147 | 343.2843 | 10.24 | $[M+H]^+$ | $C_{20}H_{38}O_4$ | 4 | -1.5 | 325.2723, 251.2372, 233.2257, 163.1481, 149.1321, 135.1162, 121.1004, 109.1017, 107.0851, 97.0989, 95.0828, 81.0697 | n.a. | | | GYM | |
| 148 | 331.2845 | 10.31 | $[M+H]^+$ | $C_{19}H_{38}O_4$ | 4 | -0.9 | Nf | n.a. | | | GYM | |

**References**


1. Altschul, S.F.; Gish, W.; Miller, W.; Myers, E.W.; Lipman, D.J. Basic local alignment search tool. *J. Mol. Biol.* **1990**, *215*, 403-410, doi: 10.1016/S0022-2836(05)80360-2.
2. Wang, Q.; Garrity, G.M.; Tiedje, J.M.; Cole, J.R. Naive Bayesian classifier for rapid assignment of rRNA sequences into the new bacterial taxonomy. *Appl. Environ. Microbiol.* **2007**, *73*, 5261-5267, doi: 10.1128/AEM.00062-07.
3. Sumner, L.W.; Amberg, A.; Barrett, D.; Beale, M.H.; Beger, R.; Daykin, C.A.; Fan, T.W.; Fiehn, O.; Goodacre, R.; Griffin, J.L.*, et al.* Proposed minimum reporting standards for chemical analysis Chemical Analysis Working Group (CAWG) Metabolomics Standards Initiative (MSI). *Metabolomics* **2007**, *3*, 211-221, doi: 10.1007/s11306-007-0082-2.
4. Seibert, S.F.; Eguereva, E.; Krick, A.; Kehraus, S.; Voloshina, E.; Raabe, G.; Fleischhauer, J.; Leistner, E.; Wiese, M.; Prinz, H.*, et al.* Polyketides from the marine-derived fungus *Ascochyta salicorniae* and their potential to inhibit protein phosphatases. *Org. Biomol. Chem.* **2006**, *4*, 2233-2240, doi: 10.1039/b601386d.
5. Niu, S.; Xia, J.M.; Li, Z.; Yang, L.H.; Yi, Z.W.; Xie, C.L.; Peng, G.; Luo, Z.H.; Shao, Z.; Yang, X.W. Aphidicolin chemistry of the deep-sea-derived fungus *Botryotinia fuckeliana* MCCC 3A00494. *J. Nat. Prod.* **2019**, *82*, 2307-2331, doi: 10.1021/acs.jnatprod.9b00705.
6. Yang, K.L.; Wei, M.Y.; Shao, C.L.; Fu, X.M.; Guo, Z.Y.; Xu, R.F.; Zheng, C.J.; She, Z.G.; Lin, Y.C.; Wang, C.Y. Antibacterial anthraquinone derivatives from a sea anemone-derived fungus *Nigrospora* sp. *J. Nat. Prod.* **2012**, *75*, 935-941, doi: 10.1021/np300103w.
7. Li, X.; Li, X.-D.; Li, X.-M.; Xu, G.-M.; Liu, Y.; Wang, B.-G. Wentinoids A–F, six new isopimarane diterpenoids from *Aspergillus wentii* SD-310, a deep-sea sediment derived fungus. *RSC Adv.* **2017**, *7*, 4387-4394, doi: 10.1039/c6ra27209f.
8. Liu, J.; Zhang, D.; Zhang, M.; Liu, X.; Chen, R.; Zhao, J.; Li, L.; Wang, N.; Dai, J. Periconiasins I and J, two new cytochalasans from an endophytic fungus *Periconia* sp. *Tetrahedron Lett.* **2016**, *57*, 5794-5797, doi: 10.1016/j.tetlet.2016.11.038.
9. Chen, S.; Zhang, Y.; Niu, S.; Liu, X.; Che, Y. Cytotoxic cleistanthane and cassane diterpenoids from the entomogenous fungus *Paraconiothyrium hawaiiense*. *J. Nat. Prod.* **2014**, *77*, 1513-1518, doi: 10.1021/np500302e.
10. Kuppers, L.; Ebrahim, W.; El-Neketi, M.; Ozkaya, F.C.; Mandi, A.; Kurtan, T.; Orfali, R.S.; Muller, W.E.G.; Hartmann, R.; Lin, W.*, et al.* Lactones from the sponge-derived fungus *Talaromyces rugulosus*. *Mar. Drugs* **2017**, *15*, 359, doi: 10.3390/md15110359.
11. Sugie, Y.; Hirai, H.; Kachi-Tonai, H.; Kim, Y.J.; Kojima, Y.; Shiomi, Y.; Sugiura, A.; Sugiura, A.; Suzuki, Y.; Yoshikawa, N.*, et al.* New pyrrolizidinone antibiotics CJ-16,264 and CJ-16,367. *J. Antibiot. (Tokyo)* **2001**, *54*, 917-925, doi: 10.7164/antibiotics.54.917.
12. MacKinnon, S. Components from the phytotoxic extract of *Alternaria brassicicola*, a black spot pathogen of canola. *Phytochemistry* **1999**, *51*, 215-221, doi: 10.1016/s0031-9422(98)00732-8.
13. Du, L.; Li, D.; Zhu, T.; Cai, S.; Wang, F.; Xiao, X.; Gu, Q. New alkaloids and diterpenes from a deep ocean sediment derived fungus *Penicillium* sp. *Tetrahedron* **2009**, *65*, 1033-1039, doi: 10.1016/j.tet.2008.11.078.
14. Lin, W.H.; Li, J.; Fu, H.Z.; Proksch, P. Four novel hydropyranoindeno-derivatives from marine fungus *Aspergillus versicolor*. *Chin. Chem. Lett.* **2001**, *12*, 435-438.
15. Ghisalberti, E.L.; Hockless, D.C.; Rowland, C.; White, A.H. Harziandione, a new class of diterpene from *Trichoderma harzianum*. *J. Nat. Prod.* **1992**, *55*, 1690-1694, doi: 10.1021/np50089a023.
16. Zhang, P.-L.; Han, Y.; Zhang, L.-T.; Wang, X.-L.; Shen, T.; Ren, D.; Lou, H.; Wang, X.-N. Botrysphones A–C and botrysphins A–F, triketides and diterpenoids from the fungus *Botrysphaeria laricina*. *J. Nat. Prod.* **2017**, *80*, 1791-1797, doi: 10.1021/acs.jnatprod.6b01196.
17. Zhao, D.L.; Shao, C.L.; Gan, L.S.; Wang, M.; Wang, C.Y. Chromone derivatives from a sponge-derived strain of the fungus *Corynespora cassiicola*. *J. Nat. Prod.* **2015**, *78*, 286-293, doi: 10.1021/np5009152.
18. Julianti, E.; Oh, H.; Jang, K.H.; Lee, J.K.; Lee, S.K.; Oh, D.C.; Oh, K.B.; Shin, J. Acremostrictin, a highly oxygenated metabolite from the marine fungus *Acremonium strictum*. *J. Nat. Prod.* **2011**, *74*, 2592-2594, doi: 10.1021/np200707y.
19. Li, D.H.; Cai, S.X.; Zhu, T.J.; Wang, F.P.; Xiao, X.; Gu, Q.Q. New cytotoxic metabolites from a deep-sea-derived fungus, *Phialocephala* sp., strain FL30r. *Chem. Biodivers.* **2011**, *8*, 895-901, doi: 10.1002/cbdv.201000134.

20. Huang, Z.J.; Shao, C.L.; Chen, Y.G.; She, Z.G.; Lin, Y.C.; Zhou, S.N. A new isocoumarin from mangrove endophytic fungus (No. dz17) on the South China Sea coast. *Chem. Nat. Compd.* **2007**, *43*, 655-658, doi: 10.1007/s10600-007-0221-z.
21. Chen, Y.S.; Cheng, M.J.; Hsiao, Y.; Chan, H.Y.; Hsieh, S.Y.; Chang, C.W.; Liu, T.W.; Chang, H.S.; Chen, I.S. Chemical constituents of the endophytic fungus *Hypoxylon* sp. 12F 0687 isolated from Taiwanese *Ilex formosana*. *Helv. Chim. Acta* **2015**, *98*, 1167-1176, doi: 10.1002/hlca.201500048.
22. Tang, Y.; Liu, Y.; Ruan, Q.; Zhao, M.; Zhao, Z.; Cui, H. Aspermeroterpenes A-C: Three meroterpenoids from the marine-derived fungus *Aspergillus terreus* GZU-31-1. *Org. Lett.* **2020**, *22*, 1336-1339, doi: 10.1021/acs.orglett.9b04648.
23. Xia, X.; Zhang, J.; Zhang, Y.; Wei, F.; Liu, X.; Jia, A.; Liu, C.; Li, W.; She, Z.; Lin, Y. Pimarane diterpenes from the fungus *Epicoccum* sp. HS-1 associated with *Apostichopus japonicus*. *Bioorg. Med. Chem. Lett.* **2012**, *22*, 3017-3019, doi: 10.1016/j.bmcl.2012.01.055.
24. McGovern, E.P.; Bentley, R. Biosynthesis of flaviolin and 5,8-dihydroxy-2,7-dimethoxy-1,4-naphthoquinone. *Biochemistry* **1975**, *14*, 3138-3143, doi: 10.1021/bi00685a016.
25. Xia, G.Y.; Wang, L.Y.; Xia, H.; Wu, Y.Z.; Wang, Y.N.; Lin, P.C.; Lin, S. Three new polyketides from the endophytic fungus *Penicillium purpurogenum*. *J. Asian Nat. Prod. Res.* **2020**, *22*, 233-240, doi: 10.1080/10286020.2019.1699535.
26. Qi, J.; Shao, C.L.; Li, Z.Y.; Gan, L.S.; Fu, X.M.; Bian, W.T.; Zhao, H.Y.; Wang, C.Y. Isocoumarin derivatives and benzofurans from a sponge-derived *Penicillium* sp. fungus. *J. Nat. Prod.* **2013**, *76*, 571-579, doi: 10.1021/np3007556.
27. Cui, C.B.; Ubukata, M.; Kakeya, H.; Onose, R.; Okada, G.; Takahashi, I.; Isono, K.; Osada, H. Acetophthalidin, a novel inhibitor of mammalian cell cycle, produced by a fungus isolated from a sea sediment. *J. Antibiot. (Tokyo)* **1996**, *49*, 216-219, doi: 10.7164/antibiotics.49.216.
28. Kakinuma, N.; Iwai, H.; Takahashi, S.; Hamano, K.; Yanagisawa, T.; Nagai, K.; Tanaka, K.; Suzuki, K.; Kirikae, F.; Kirikae, T., *et al.* Quinolactacins A, B and C: novel quinolone compounds from *Penicillium* sp. EPF-6. I. Taxonomy, production, isolation and biological properties. *J. Antibiot. (Tokyo)* **2000**, *53*, 1247-1251, doi: 10.7164/antibiotics.53.1247.
29. Chen, Z.; Zheng, Z.; Huang, H.; Song, Y.; Zhang, X.; Ma, J.; Wang, B.; Zhang, C.; Ju, J. Penicacids A-C, three new mycophenolic acid derivatives and immunosuppressive activities from the marine-derived fungus *Penicillium* sp. SOF07. *Bioorg. Med. Chem. Lett.* **2012**, *22*, 3332-3335, doi: 10.1016/j.bmcl.2012.02.106.
30. Pettit, G.R.; Hogan, F.; Xu, J.P.; Tan, R.; Nogawa, T.; Cichacz, Z.; Pettit, R.K.; Du, J.; Ye, Q.H.; Cragg, G.M., *et al.* Antineoplastic agents. 536. New sources of naturally occurring cancer cell growth inhibitors from marine organisms, terrestrial plants, and microorganisms. *J. Nat. Prod.* **2008**, *71*, 438-444, doi: 10.1021/np700738k.
31. Cueto, M.; MacMillan, J.B.; Jensen, P.R.; Fenical, W. Tropolactones A–D, four meroterpenoids from a marine-derived fungus of the genus *Aspergillus*. *Phytochemistry* **2006**, *67*, 1826-1831, doi: 10.1016/j.phytochem.2006.01.008.
32. Ozkaya, F.C.; Ebrahim, W.; Klopotowski, M.; Liu, Z.; Janiak, C.; Proksch, P. Isolation and X-ray structure analysis of citreohybridonol from marine-derived *Penicillium atrovenetum*. *Nat. Prod. Res.* **2018**, *32*, 840-843, doi: 10.1080/14786419.2017.1311893.
33. Liu, Z.G.; Bao, L.; Liu, H.W.; Ren, J.W.; Wang, W.Z.; Wang, L.; Li, W.; Yin, W.B. Chemical diversity from the Tibetan Plateau fungi *Penicillium kongii* and *P. brasilianum*. *Mycology* **2018**, *9*, 10-19, doi: 10.1080/21501203.2017.1331937.
34. Xu, X.; Zhang, X.; Nong, X.; Wang, J.; Qi, S. Brevianamides and mycophenolic acid derivatives from the deep-sea-derived fungus *Penicillium brevicompactum* DFFSCS025. *Mar. Drugs* **2017**, *15*, 43, doi: 10.3390/md15020043.
35. Cheng, Z.; Xu, W.; Wang, Y.; Bai, S.; Liu, L.; Luo, Z.; Yuan, W.; Li, Q. Two new meroterpenoids and two new monoterpenoids from the deep sea-derived fungus *Penicillium* sp. YPGA11. *Fitoterapia* **2019**, *133*, 120-124, doi: 10.1016/j.fitote.2018.12.022.
36. Kosemura, S. Meroterpenoids from *Penicillium citreo-viride* B. IFO 4692 and 6200 hybrid. *Tetrahedron* **2003**, *59*, 5055-5072, doi: 10.1016/S0040-4020(03)00739-7.
37. Kim, E.L.; Li, J.L.; Dang, H.T.; Hong, J.; Lee, C.O.; Kim, D.K.; Yoon, W.D.; Kim, E.; Liu, Y.; Jung, J.H. Cytotoxic cytochalasins from the endozoic fungus *Phoma* sp. of the giant jellyfish *Nemopilema nomurai*. *Bioorg. Med. Chem. Lett.* **2012**, *22*, 3126-3129, doi: 10.1016/j.bmcl.2012.03.058.
38. Evidente, A.; Andolfi, A.; Vurro, M.; Zonno, M.C.; Motta, A. Cytochalasins Z4, Z5, and Z6, three new 24-Oxa[14]cytochalasans produced by *Phoma exigua* var. *heteromorpha*. *J. Nat. Prod.* **2003**, *66*, 1540-1544, doi: 10.1021/np030252o.

39. Kim, E.L.; Wang, H.; Park, J.H.; Hong, J.; Choi, J.S.; Im, D.S.; Chung, H.Y.; Jung, J.H. Cytochalasin derivatives from a jellyfish-derived fungus *Phoma* sp. *Bioorg. Med. Chem. Lett.* **2015**, *25*, 2096-2099, doi: 10.1016/j.bmcl.2015.03.080.
40. Xu, S.; Ge, H.M.; Song, Y.C.; Shen, Y.; Ding, H.; Tan, R.X. Cytotoxic cytochalasin metabolites of endophytic *Endothia gyrosa*. *Chem. Biodivers.* **2009**, *6*, 739-745, doi: 10.1002/cbdv.200800034.
41. Amagata, T.; Tanaka, M.; Yamada, T.; Doi, M.; Minoura, K.; Ohishi, H.; Yamori, T.; Numata, A. Variation in cytostatic constituents of a sponge-derived *Gymnascella dankaliensis* by manipulating the carbon source. *J. Nat. Prod.* **2007**, *70*, 1731-1740, doi: 10.1021/np070165m.
42. Cho, K.W.; Lee, H.S.; Rho, J.R.; Kim, T.S.; Mo, S.J.; Shin, J. New lactone-containing metabolites from a marine-derived bacterium of the genus *Streptomyces*. *J. Nat. Prod.* **2001**, *64*, 664-667, doi: 10.1021/np000599g.
43. Takada, K.; Ninomiya, A.; Naruse, M.; Sun, Y.; Miyazaki, M.; Nogi, Y.; Okada, S.; Matsunaga, S. Surugamides A-E, cyclic octapeptides with four D-amino acid residues, from a marine *Streptomyces* sp.: LC-MS-aided inspection of partial hydrolysates for the distinction of D- and L-amino acid residues in the sequence. *J. Org. Chem.* **2013**, *78*, 6746-6750, doi: 10.1021/jo400708u.
44. Brumley, D.; Spencer, K.A.; Gunasekera, S.P.; Sauvage, T.; Biggs, J.; Paul, V.J.; Luesch, H. Isolation and characterization of anaephenes A-C, alkylphenols from a filamentous cyanobacterium (*Hormoscilla* sp., Oscillatoriales). *J. Nat. Prod.* **2018**, *81*, 2716-2721, doi: 10.1021/acs.jnatprod.8b00650.
45. Zhang, W.; Che, Q.; Tan, H.; Qi, X.; Li, J.; Li, D.; Gu, Q.; Zhu, T.; Liu, M. Marine *Streptomyces* sp. derived antimycin analogues suppress HeLa cells via depletion HPV E6/E7 mediated by ROS-dependent ubiquitin-proteasome system. *Sci. Rep.* **2017**, *7*, 42180, doi: 10.1038/srep42180.
